# Pleistocene glaciation of Fenland, England, and its implications for evolution of the region

**DOI:** 10.1098/rsos.170736

**Published:** 2018-01-24

**Authors:** P. L. Gibbard, R. G. West, P. D. Hughes

**Affiliations:** 1 Cambridge Quaternary, Department of Geography, University of Cambridge, Cambridge CB2 3EN, UK; 2 Scott Polar Research Institute, University of Cambridge, Lensfield Road, Cambridge CB2 1ER, UK; 3 3A Woollards Lane, Great Shelford, Cambridge CB22 5LZ, UK; 4 Quaternary Environments and Geoarchaeology Research Group, Department of Geography, The University of Manchester, Manchester M13 9PL, UK

**Keywords:** Quaternary, Pleistocene, glaciation, stratigraphy, sedimentation, palaeoenvironment

## Abstract

Detailed investigation of landforms and their underlying deposits on the eastern margin of Fenland, East Anglia, demonstrated that they represent a series of glaciofluvial delta-fan and related sediments. Associated with these deposits are glacially dislocated sediments including tills, meltwater and pre-existing fluvial sediments. These ‘Skertchly Line’ deposits occur in the context of a substantial ice lobe that entered Fenland from the N to NE, dammed the streams entering the basin and caused glacial lakes to form in the valleys on the margins. Bulldozing by the ice lobe caused a series of ice-pushed ridges to form at the dynamic margin, especially at the ice maximum and during its retreat phases. Meltwater formed a series of marginal fans that coalesced into marginal accumulations in the SE of the basin. The ice lobe is named the Tottenhill glaciation. Further investigations of the Fenland margin have revealed the extent of the Tottenhill glaciation in the Fenland Basin, to the south and west, in sufficient detail to demonstrate the nature of the Tottenhill ice lobe and the landscape left on deglaciation. The ice lobe is likely to have been prone to surging. This is indicated by the low gradient of the ice lobe, the presence of underlying ductile Mesozoic clays, the evidence of ice-marginal flooding and the presence of arcuate glaciotectonic push moraines. Regional correlation, supported by independent numerical geochronology, indicates that the glaciation occurred *ca* 160 ka, i.e. during the late Middle Pleistocene, Marine Isotope Stage (MIS) 6, the Wolstonian Stage. Comparison and correlation across the southern North Sea Basin confirms that the glaciation is the equivalent of that during the Late Saalian Drenthe Stadial in The Netherlands. The implications of this correlation are presented. Before the glaciation occurred, the Fenland Basin did not exist. It appears to have been initiated by a subglacial tunnel valley system beneath the Anglian (=Elsterian, MIS 12) ice sheet. During the subsequent Hoxnian (=Holsteinian; approx. MIS 11) interglacial, the sea invaded the drainage system inherited following the glacial retreat. The evolution through the subsequent *ca* 200 ka Early to Middle Wolstonian substages, the interval between the Hoxnian (Holsteinian) temperate Stage and the Wolstonian glaciation, represents a period during which fluvial and periglacial activity modified the landscape under cold climates, and organic sediments were laid down during a warmer event. Palaeolithic humans were also periodically present during this interval, their artefacts having been reworked by the subsequent glaciation. The deglaciation was followed by re-establishment of the rivers associated with the deposition of Late Wolstonian (Warthe Stadial) gravels and sands, and later, deposits of the Ipswichian interglacial (=Eemian, approx. MIS 5e) including freshwater, then estuarine sediments. Subsequent evolution of the basin occurred during the Devensian Stage (=Weichselian, MIS 5d-2) under predominantly cold, periglacial conditions.

## 1. Introduction

The Fenland of East Anglia is a geological feature of remarkable interest, belying the apparent lack of striking geological features in the low-lying landscapes of the area. The Fenland receives rivers from the Chalk uplands to the east and south and from the Lower Cretaceous and Jurassic uplands to the west. The rivers discharge into the wide bay of the Wash, itself an important geological feature, and so drain to the North Sea. With extensive areas now slightly above and slightly below the present sea level, enclosing minor islands, the geology is sensitive to changes in sea level. These have affected the nature of sediments deposited in the Fenland in the last half million years, a period covering much sea-level change associated with the growth and decay of Middle and Late Pleistocene ice sheets.

The geology of the Holocene, and succeeding the last (Devensian) glaciation beginning at *ca* 11.7 ka, shows the accumulation of freshwater and brackish sediments in a well-marked basin in the Fenland. This complex sequence of peats and inorganic sediments has been described in detail by Godwin [1], Gallois [2], Waller [3] and Wheeler [4]. Notable is the archaeological sequence from the Mesolithic onwards associated with Fenland sediments, an indication of the favourable living conditions of the Fenland over Holocene time.

However, the earlier history and the origin of the Fenland have not been so analysed, apart from an early account by Skertchly [5]. It is now clear that the Fenland area has been invaded by ice on a number of occasions. The invasions are linked with the formation of the Wash, the forming of the Fenland Basin and the deposition of glacial sediments. There are also periods of interglacial and periglacial climates, periods of low sea level and periods of marine transgression leading to a complex history involving landscape evolution, climatic change and sea-level change.

The present contribution provides a synthesis of the history of the major basin forming the southern part of the Fenland from pre-Anglian to pre-Holocene times. The analysis of the large amount of data which has accumulated since Skertchly's times presents a considerable problem. We have chosen to base the analysis on the stratigraphical system propounded by the Geological Society's Special Report No. 4 on the correlation of Quaternary deposits in the British Isles [6], with a division of the Pleistocene into Stages. We have given primacy to reports giving particular detail to lithostratigraphy and biostratigraphy, and which refer to the geology of the locality of sites. We recognize that there are contentious issues involved, including the extent and sediments of the Wolstonian Stage, the origin of the ridge of gravels on the eastern margin of Fenland known as the Skertchly Line (discussed by [7]), problems arising from the presence of reworked fossil floras and faunas in Fenland gravels, making it necessary to recognize this situation and deal with the data with particular care, and, finally, problems arising from the too-ready correlation of sites with a Marine Isotope Stage (MIS, a global scale) and the reliability of numerical dating.

**Figure 1.**
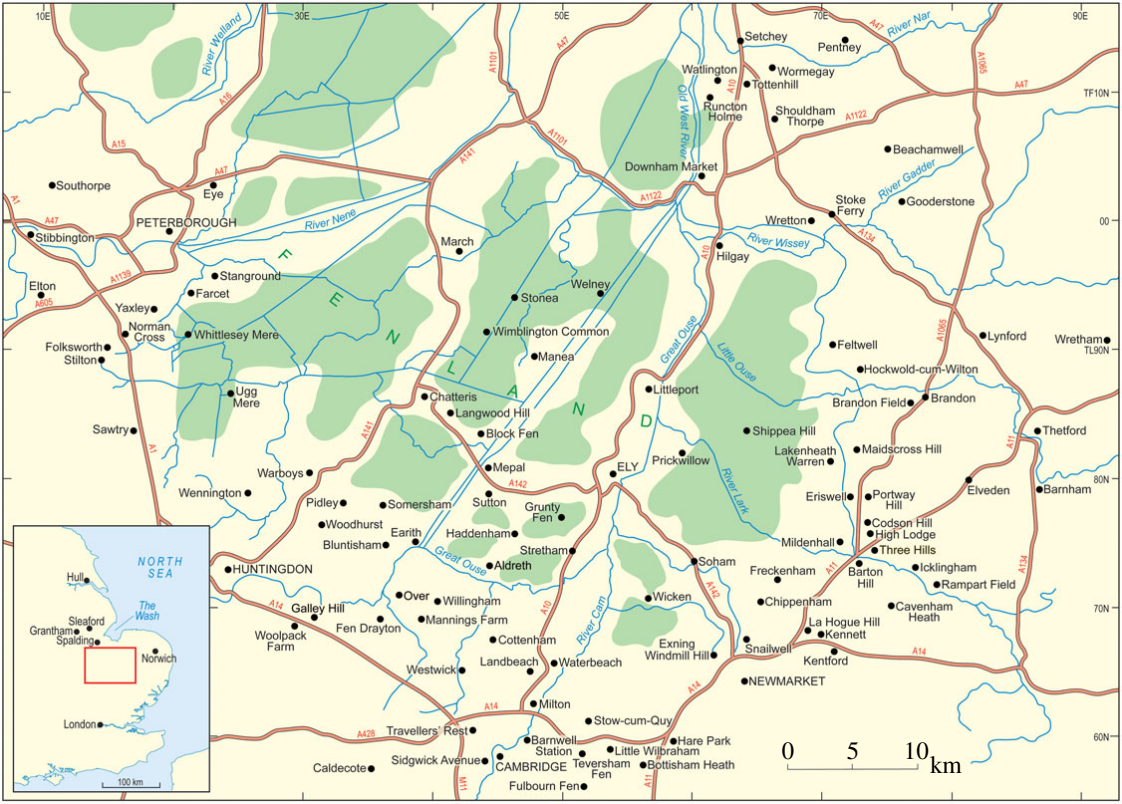
Location map showing the area studied and localities mentioned in the text. The green areas are those below sea level.

**Figure 2.**
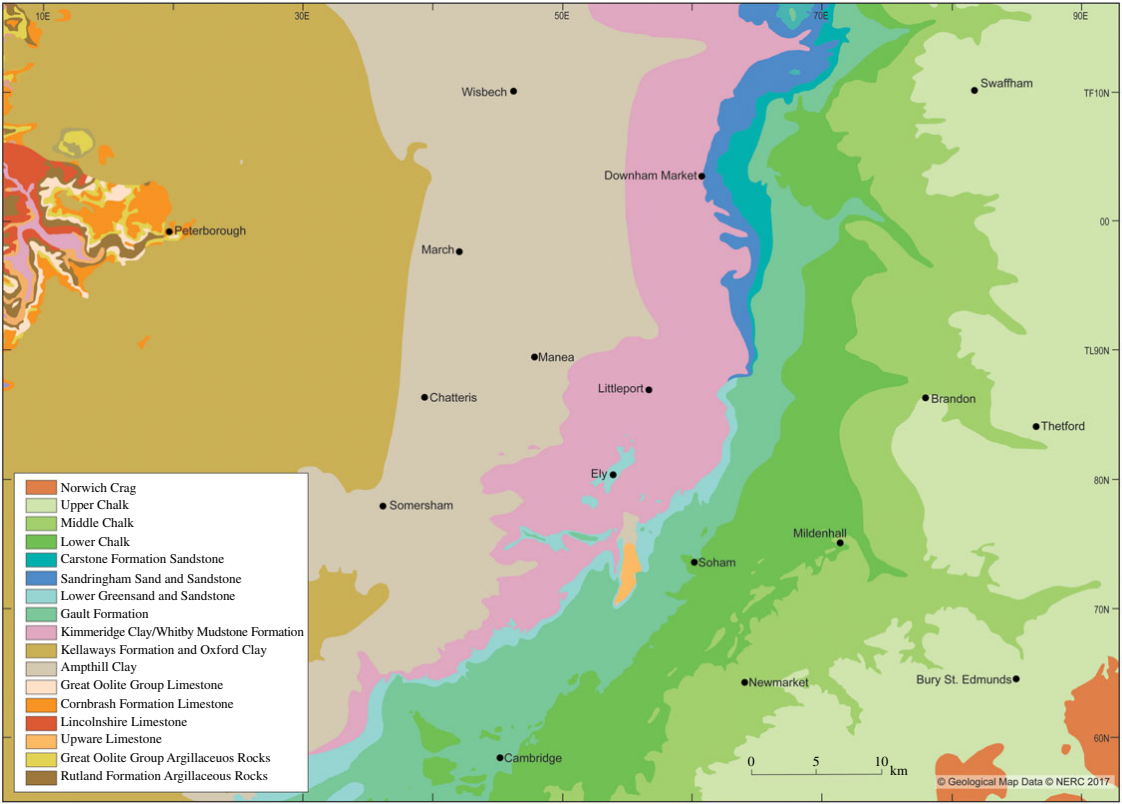
Bedrock geology modified from the British Geological Survey—source EDINA.

**Figure 3.**
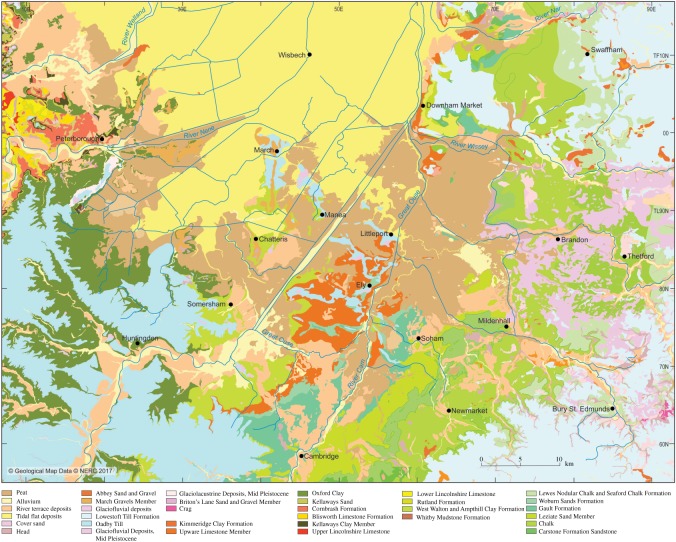
Quaternary and bedrock geological map of Fenland showing the ridges, depressions and gravel spreads. Map source EDINA. An enlargement of the area around Mildenhall is shown in figure 10.

Table 1 summarizes the stratigraphy of this period. The present study of Fenland covers the area shown in figure 1, while figure 2 shows the bedrock geology of the area and figure 3 the Quaternary geology.

**Table 1.** Geological timetable of events in the Fenland Basin and their correlation to the near Continent during the Middle to Late Pleistocene. The NW European chronostratigraphy is based on Litt *et al*. [8,9]. For further explanation see text.

British chronostratigraphy/climatostratigraphy		fluvial/events	marine/incursion events	glacial/aeolian events	climate	human occupation	continental chronostratigraphy/climatostratigraphy		approx. Marine Isotope (sub-) Stage (MIS)
Devensian	Late	Downcutting and aggradation of gravel and sand in river valleys		? Loess/cover sand deposition locally	periglacial	?	Weichselian		∼1–2
		Downcutting and aggradation of gravel and sand in river valleys and lacustrine sedimentation		Devensian glaciation *s.s.* lacustrine ponding of river valleys	periglacial/glacial				
	Middle	Downcutting and aggradation of gravel and sand in river valleys			periglacial/interstadial	*			3
		Deposition of interstadial deposits			interstadial				∼4–5d
	Early	Downcutting and aggradation of gravel and sand in river valleys			periglacial				
Ipswichian		Aggradation of temperate floodplain and channel sediments	estuarine sedimentation		temperate		Eemian		∼5e
Wolstonian	Late	Downcutting and aggradation of gravel and sand in river valleys		? Loess/cover sand deposition locally	periglacial		Warthe Stadial	Saalian	6
		*Non-deposition*							
		Downcutting and aggradation of gravel and sand in river valleys			periglacial				
		*Non-deposition*		Wolstonian glaciation *s.s.* lacustrine ponding of river valleys	periglacial/glacial		Drenthe Stadial		
		Downcutting and aggradation of gravel and sand in river valleys and lacustrine sedimentation							
		Deposition of High Lodge silt deposits			temperate	*	?Schöningen Interstadial/Interglacial		∼7
Wolstonian	Early/Middle	Downcutting and aggradation of gravel and sand in river valleys			periglacial	*		Saalian	8 to ∼11b
		*Non-deposition*							
		Downcutting and aggradation of temperate floodplain and channel sediments ? and lacustrine sediments			periglacial/?temperate	*	?Bantega Interstadial		
		*Non-deposition*							
		Downcutting and aggradation of gravel and sand in river valleys and lake basin infill with fine-grained sedimentation in river valleys, etc. during interglacial events			interstadial/temperate	?	?Wacken/Dömnitz/Reinsdorf/Hoogeveen Interstadial/Interglacial		
				? Loess deposition locally	periglacial	?	Fuhne Stadial		
Hoxnian		Infill of lake basins, incoherent river system	estuarine sedimentation		temperate	*	Holsteinian		∼11c
Anglian		Tunnel valley formation, glaciofluvial and glaciolacustrine deposition		Glaciation of lowland eastern England	periglacial/glacial		Elsterian		12
pre-Anglian		*Non-deposition*					pre-Elsterian		pre-12
		Downcutting and aggradation of gravel and sand in river valleys			periglacial

## 2. Glaciation and interglacials of Fenland

In lowland south and eastern Britain, two major glaciations have been identified during the Middle Pleistocene, the earliest and most extensive of which occurred during the Anglian Stage (= Elsterian). The second occurred during the late Middle Pleistocene, intermediate between the Hoxnian (=Holsteinian) and Ipswichian (=Eemian) interglacial Stages. This Wolstonian glaciation was originally recognized in the English Midlands, though the deposits were also thought by some to date from the Anglian Stage (e.g. [10]). Further, a younger Middle Pleistocene glacigenic sequence is known from the Midlands, Yorkshire and northern East Anglia [11].

Recent studies have demonstrated a series of gravel and sand accumulations that underlie characteristic landforms occurring along the margin of Fenland in eastern England [12–18]. These presented evidence for late Middle Pleistocene glaciation in Fenland (figure 1) and showed that the setting, morphology and internal architecture of these landforms represent glacio-marginal successions. The accumulations mark a distinct glacial maximum limit, named the Skertchly Line, which formed where an ice lobe, flowing from the north, terminated at the basin margin. This advance dammed a series of streams draining into the Fenland to form proglacial lakes in contact with the ice front. Meltwater discharges from portals in the ice margin formed a series of ice-contact, fan-like deltaic and related accumulations. This was termed the Tottenhill glaciation, originally described from the site of the same name [13], has been shown to be of Late Wolstonian age (i.e. Late Saalian) [11–15,17,19,20]. The deposits have been assigned to the Feltwell Formation [15,17] (appendix A).

The identification of this glaciation holds significant implications for the Pleistocene stratigraphy and palaeogeographical evolution of the Fenland region. In particular, the development of the Fenland Basin before, during and following the glaciation will be analysed.

The Wolstonian Stage is a critical interval in the landscape evolution of eastern England, during which the modern drainage system was established following both the Anglian glaciation and the immediately following Hoxnian Stage interglacial [18,21]. Subsequent Ipswichian Stage (Eemian) interglacial sequences, occurring at or close to modern floodplain level in river valleys throughout the region, confirm that the present drainage system was established by this time. The occurrence of the Tottenhill glaciation in the Fenland, postdating the temperate Hoxnian temperate Stage and various cold-stage fluvial and related accumulations, and the High Lodge interstadial/interglacial event [17] indicates that the Fenland Basin was subjected to a series of evolutionary phases through this late Middle Pleistocene interval (*ca* 260 ka overall). Subsequent valley evolution, incision and accompanying fluvial sediment deposition occur throughout this time in the region.

On the basis of the occurrence of artefact assemblages, it appears that Palaeolithic humans were also present through the pre-Wolstonian glaciation episode. Assemblages recovered from the majority of the sites described occurred in the chalk-rich delta-fan gravels, indicating that they were reworked, together with other clastic material, from local sources by glacial action (appendix B).

The subsequent post-glaciation evolution of the regional landscape region occurred predominantly under a periglacial regime during which gravel-capped hills remained while the surrounding chalk surfaces were degraded. Severe permafrost conditions are indicated by the occurrence of ice-wedge casts in the sediments, fluvial incision and deposition by streams and the burial of the deposits beneath a stratum of aeolian cover sand [14,22]. These events occurred in the latest Wolstonian period during the interval following the glaciation and before the climatic amelioration of the Ipswichian interglacial (i.e. *ca* 20 ka), and during the last cold stage (Devensian, Weichselian).

The geology of the eastern and southeastern margins of the Fenland is treated first, followed by descriptions of the margin to the south against the Chalk escarpment and to the west against the Mesozoic claylands. The geology of areas within Fenland, particularly the gravels and tills, are considered in separate detail.

## 3. The eastern margin of Fenland: the Skertchly Line

### 3.1. The Skertchly Line and related sites

Before describing the Skertchly Line sites in detail, it should be emphasized that an alternative explanation for the origin of the Skertchly Line gravels has interpreted them as fluviatile gravels of a former, pre-Anglian age, river which flowed down the east margin of the Fenland Basin, and survived overriding by ice of the Anglian Glaciation [23–26]. The arguments for and against this alternative explanation have been widely discussed in recent publications (e.g. [14–16,20,24,27]).

The investigations of a series of sites on the eastern and southeastern Fenland margin (figure 1) clearly demonstrate a range of characteristics in common, namely morphology, internal stratigraphy and lithology, summarized below. The individual sites have been described in detail by Gibbard *et al.* [12–16] and West *et al*. [17] to which reference should be made for complete site details. The site descriptions include facies codes (modified from [28,29]). The sites have been studied using field and laboratory techniques supported by ground-penetrating radar investigation.

#### 3.1.1. Morphology

A key characteristic of the landforms described is their overall form. They can be divided into two broad, yet closely related groups. Most commonly they occur as low hills, with W–NW-facing slopes markedly steep in contrast to those to E, S and NE which are relatively gentle. This form is typically seen at Codson Hill, Maidscross Hill, Feltwell, Tottenhill, Shouldham, Barton Hill and La Hogue Hill. Portway Hill, with the neighbouring Foxhole Heath, has a subdued, complex ridge-like morphology aligned broadly N–S, but like neighbouring Codson Hill it is asymmetric in the W–E cross section.

The second type show a more elongated form, often comprising a series E–W elongate ridge complexes again showing the asymmetrical long axis. An example of the latter is the hills east of Hockwold-cum-Wilton. By contrast, Snailwell village is sited on an elongate ridge aligned ENE–WSW but which again shows a broadly N–S asymmetry of surface slope, a feature it shares with the adjacent Windmill Hill, Exning, which is an elongate ridge aligned NNE–SSW. Apparently transitional between the two landform types is the Warren Hill–Three Hills Ridge. It is elongated N–S, has an E–W profile that is generally smoothly convex, but again asymmetric, with a shallower eastern-facing slope. Examination of the topography both in the field and on air photographs indicates a distinct sharp break of slope on the west-facing slope of the ridge.

A second characteristic these features share is their maximum altitude of their uppermost surfaces, repeatedly reaching a level of 30–35 m O.D. Exceptionally they are lower, e.g. Tottenhill reaches only 12 m O.D., while nearby Shouldham Thorpe reaches 35 m O.D. and Kentford 42 m O.D. [30].

#### 3.1.2. Internal stratigraphy

The internal sequences of deposits forming these hill and ridge-like forms are variable, but again several characteristic similarities can be identified that unify their succession. The deposits have been defined as the Feltwell Formation ([15,16]; appendix A).

**Figure 4.**
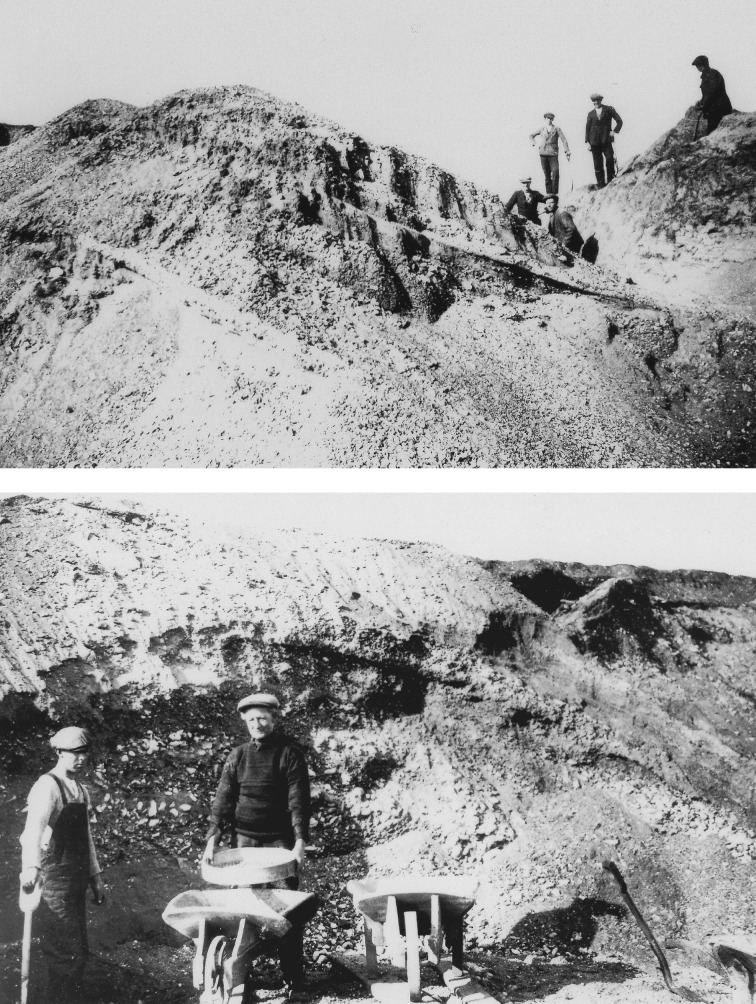
Photographs of the sections exposed at Three Hills (Warren Hill), Suffolk, showing the steeply inclined bedding planes in chalk-rich gravels and sands noted by previous authors. See text for details. (Reproduced with permission from the British Geological Survey.)

The principal characteristic of these features was originally described by Solomon [31] and Paterson [32] as typically including gravels with a high proportion of Chalk clasts, disposed into steeply inclined bedding planes (figure 4). This observation was confirmed by Wymer *et al*. [33] who noted the gravels at Three Hills (Warren Hill) were ‘highly variable and characterized by “large scale” foreset beds of coarse, clast-supported, open framework gravel’. This was also recorded by Gibbard and co-workers [12–17] and extended to many of the sites in the Skertchly Line ridges. In addition, these authors noted the consistently repeated pattern of sediment facies distribution across the localities, the deposits occurring as fan-like accumulations in planform. In the W, NW and in the south, N (proximal)-sides the sequences predominantly comprise laminated silts at the base, overlain by horizontally bedded sands that pass sharply upwards into the substantial foreset-bedded coarse gravel already mentioned. This gravel may be locally very coarse, including boulders of chalk, such as at Feltwell. Towards the distal E, S, SE and NE landform sides, this sequence continues with the silts thickening, grading into stratified sands and the overlying gravel fines markedly in the down-current direction, as indicated by the palaeocurrent evidence. The present ground surfaces decline in the same direction. This systematic eastwards or southwards overall fining of the sedimentary assemblage implies derivation from single-point sources to the NW–N of the sites. These deposits have been interpreted as glaciofluvial delta-fan complexes.

Associated with the sorted sediments are glacial diamicton (till) beds, and mass-flow deposits of varying lithology. This diamicton frequently occurs on the proximal, i.e. W- to NW-facing slopes, and either beneath, as at High Lodge and Codson Hill, or above as at Feltwell, Hockwold and Kentford, the fan-delta sediments. For example, Paterson [32] saw thick till on the proximal side of the fan-delta sediments at Codson Hill. Where they have been observed, these diamictons are predominantly of lodgement facies. This conclusion is based on their high compaction, the abundance of striated and streaked-out chalk clasts, the occurrence of local lithologies, shearing and the basal incorporation of underlying sediment, e.g. at Feltwell. At High Lodge and Codson Hill the diamicton exceeds 2 m in thickness. Other diamicton facies also occur and include units interstratified with the sorted sediments, e.g. at Hockwold, Feltwell and Kentford. Here their massive structure, sand matrix and relationship to adjacent strata suggest that they represent mass flows (flow-till facies) on foreset surfaces. Likewise, the Mildenhall ‘upper diamicton’ at High Lodge appears to have a similar origin [34,35].

The diamicton varies in colour, texture and clast content across the area, these properties apparently reflecting incorporation of local materials. However, the diamicton is predominently yellow-brown to red-brown in colour, possibly derived by reworking of local Lower Cretaceous and Jurassic rocks, particularly the ‘Red Chalk’, occurring immediately to the north of the area at Codson Hill, Hockwold and Feltwell. This red-brown coloration contrasts with the typical grey to blue-grey hues of the more widespread, Anglian-age Lowestoft Formation tills.

At several sites the sedimentary sequence also includes deformation structures, mainly thrusts and folds. At High Lodge, where these features were best exposed and described by Lewis [34] and Francis [35], the structures represented lateral compression of pre-existing sediments. Here they indicate foreshortening directed from the W to SW. Similar, glaciotectonic structures have also been recorded from several sites, including Hockwold, Codson Hill and in particular at Three Hills [14,15]. At the last site, low-angle thrusts also occur in the upper 4 m of the underlying Chalk bedrock, while at the first two sites the structures apparently displace diamicton. At Three Hills thrusting also affects the diamicton and associated delta-fan sediments [14]. Together this structural evidence consistently indicates repeated glacial pushing and to some extent overriding by ice advancing from the direction N, NW and locally SW, throughout the area, producing push-moraine-type landforms.

Finally, periglacial stuctures and solution-collapse features also affect the sediment sequences. At Feltwell substantial, penecontemporaneous collapse structures were exposed in excavations. The infill of these features comprised material let down from above, either as coherent sediment units or more commonly as a disturbed, mixed mass of sand and pebbly sediments. These doline-like enclosed hollows indicate that solutional collapse of Chalk bedrock is frequent throughout the district. Similar features have been identified elsewhere, especially beneath the Warren Hill ridge at High Lodge, where they predate glacial deposition [17] (cf. below), implying there are several generations of these forms.

The occurrence of cryoturbation of the uppermost 1–2 m of sediments, ice-wedge casts in the sediments and the burial of the deposits beneath a widespread aeolian cover sand is recorded at several localities, e.g. Methwold Hythe, Feltwell. At Barton Hill a reactivation surface occurs at a 40 cm depth below modern ground surface, showing a possible palaeo-land surface. Finally, a disrupted relict palaeosol is also developed on the surface of the Tottenhill and Shouldham sediments, which represents a single phase of temperate weathering in addition to that of the Holocene [19].

#### 3.1.3. Lithology

The lithology of the gravel clasts has been determined from the 8–16 mm fraction from each site ([12–16] and [17] for details).

The gravels of the eastern Fenland Basin margin are generally dominated by angular flint, exotic material (mostly quartz, quartzite and volcanic/metamorphic) and chalk. Although the lithological assemblages from the fan-complex gravels are variable in the pebble grades, in general, their materials are of local origin, either derived from local Mesozoic bedrock or from the underlying Lowestoft Formation deposits. Many of the samples represent a mixture of two main components, and some an admixture of three, because many samples include a substantial exotic component. Chalk is the major non-durable component of the bedload during water transport, and an elevated proportion of chalk clasts in gravels indicates a local source of eroding chalk bedrock. At Methwold Hythe, gravel almost totally composed of chalk clasts was present. Flint-rich gravels, by contrast, are usually interpreted as being mostly far-travelled, being sourced locally but having lost any non-durable chalk component.

Exotic lithologies including quartz, quartzite and volcanic/metamorphic rocks represent components transported by river from the Midlands and/or by glaciation from northern Britain. A further possible source of exotic pebbles is the Lower Greensand in the region [36–39]. A small proportion of lithologies are derived from various Lower Cretaceous and Jurassic strata that occur within 20 km of the eastern Fenland Basin margin.

The occurrence of quartz and quartzite pebbles in these assemblages, particularly at the sites between Barton Hill and Shouldham where they represent 30% of the total, is significant. These counts correspond to earlier determinations and have been particularly associated with eastward transport of material from the East Midlands by a preglacial river system (e.g. [14,25,40–45]).

Interpretation of the fan sediments as glacio-marginal deltaic deposits implies that the quartzitic pebbles must have been derived either directly from the Midlands' source Triassic rocks or reworked locally, together with the other components of these fan-gravel assemblages. This quartz-rich material must have been incorporated by the Fenland Tottenhill ice lobe during its advance, together with locally derived flint and chalk. This is confirmed by the occurrence of these lithologies in the diamicton associated with the meltwater deposits. The restricted distribution and abundance of the quartzitic pebbles implies that the original locality from which they were derived was up-glacier a few kilometres to the NW–WNW, i.e. north of Ely and Littleport [15,16]. The absence of the quartz and quartzite component from the Tottenhill gravel [13] contrasts with its presence at Shouldham Thorpe, only 3.6 km to the south. This implies that the quartz-bearing deposits were potentially derived from preglacial fluvial sediments capping high ground forming a WNW to W-ward extension of the ridge that today forms the interfluve between the Nar and Wissey valleys, as well as possibly material occurring in the Wash [15,16]. This derivation is confirmed by heavy-mineral analyses conducted by Solomon [31].

#### 3.1.4. Interpretation of the Skertchly Line sediments

Overall, the majority of the sequences indicate subaquatic deposition in a series of Gilbert-type delta-like fans that originated from an ice front immediately in the eastern Fenland Basin to the W or NW. The fan-like planforms of the units are aligned towards the E–SSE or S. The gravel aggradations occur immediately east to south of the ridge crests, while the central and particularly the western areas at lower elevations received only sands and silts. The general absence of horizontally bedded topset facies may indicate rapid retreat of the source, i.e. the glacial ice lobe, falling water levels in the proglacial lake basins or the subsequent erosion of the sediments.

Although the sedimentary architecture, landform morphology and associated evidence together demonstrate that the sediments at almost all the Skertchly Line sites are of deltaic and related origin, there is, however, one exception. The sequence at Shouldham has been interpreted as a subaerial fan of similar origin to the other features [15,16].

#### 3.1.5. Rampart Field

In addition to the Skertchly Line sites, that at Rampart Field Town Pit (NGR TL 790715) in the Lark Valley at Icklingham, Suffolk, requires description. Excavations on the north side of the River Lark have been known since the mid-nineteenth century. The exposures were reported by Prestwich ([46]; [30,47]), associated with finds of Palaeolithic hand-axes. The sequence is no longer exposed but was described by Paterson [32] and most recently reconsidered by West [7]. The ground surface occurs at approximately 10 m above the local river valley floodplain. Several excavations were observed by Paterson in the 1930s as consisting of ‘loose, coarsely current-bedded, and sometimes steeply foreset, chalky gravels and sands with subangular sand grains and very many Bunter pebbles’. As West [7] notes, this description is important because it stresses the link and similarity with the sequences described from nearby sites including Three Hills (figure 4), Barton Hill, Codson Hill, Maidscross Hill and Brandon Field. Glacial diamicton (till) has been described from this exposure interstratified with the gravel and sands (BGS 1 : 10000 sheet TL 77 SE).

New research at this site was carried out in 2015 using ground-penetrating radar, the results indicating that the area is underlain by a complex where deltaic deposits abut Chalk bedrock.

These observations imply that the Rampart Field sequence represents deposition comparable to that observed at the Skertchly Line sites to the west and north, including Barton Hill, Three Hills, Codson Hill, etc. In other words, the sequence represents a subaquatic delta-fan laid down at an ice front standing in the Lark Valley glacial lake. A series of low till-cored hills occur further towards the SW and mark a possible extension of the ice margin of approximately 6 km.

### 3.2. Proglacial lakes and drainage

Stratified fine to medium sands are present in the valleys and associated low landscapes currently draining westwards into the Fenland. They are interpreted as deposits of proglacial lakes ponded by the Fenland glaciation.

#### 3.2.1. Nar Valley

At the mouth of the Nar Valley at Tottenhill, stratified sands at a height of a few metres O.D. were recorded by Gibbard *et al*. [13]. They were associated with a subaquatic fan at the ice front. Eastwards up the valley, in the area of Narborough Field, spreads of sand, up to 1.0 m thick, occur at heights of approximately 20–25 m O.D., associated with patterns related to ground-ice formation [22].

#### 3.2.2. Wissey valleys

In the area around Beachamwell, north of Gooderstone, the Wissey tributary valleys show an extensive cover of sand [22]. In certain areas, the sand is much thicker. At Caldecote it is over 2 m thick, resting on disturbed Chalk; here it is stratified and shows couplets of sand and pale silty sand, at a height of 11–13 m O.D. Near Gooderstone, in the tributary Gadder Valley, stratified sands are present at a height of 18 m O.D., with evidence of a shoreline. Sand depths in this area reach over 0.5 m at heights around 19 m O.D. The distribution and height of the sand suggest the presence of a proglacial lake, reaching a height of approximately 20 m O.D., named the Shingham Lake. To the south, in the main Wissey Valley at Lynford, near Ickburgh, Lewis [48] has described the Plantation Sands, a bed on the southern flank of the valley with up to approximately 6 m depth of fine to medium sands, banked against a rising slope of chalky diamicton/Chalk. The sands reach a maximum height of approximately 20 m O.D. Two optically stimulated luminescence (OSL) dates from the sands, 169 and 176 ka, support their correlation with the time of the Tottenhill glaciation [49].

#### 3.2.3. Little Ouse Valley

This valley contains a significant body of sand, the Lopham Sands, deposited in a lake, the Little Ouse Lake, ponded by the ice. The lake extended to the source of the Little Ouse at Lopham Ford, across the divide at Lopham Ford at approximately 25 m O.D. and overflowed along the Waveney Valley, through an overflow channel at Brockdish, to the North Sea. The thickness is very variable, reaching over 10 m in places. The sands are massive, showing fine stratification, fine to medium in grade, with occasional fine gravel strings and dropstones. A detailed description of the Lopham Sands is given by West [50].

#### 3.2.4. Lakenheath Warren and the area between the Little Ouse and the Lark valleys

In his classic studies of Breckland soils, Watt [51] describes and illustrates soil profiles on Lakenheath Warren, which lies in an extensive dry valley breaching the Chalk escarpment 5 km south of the Little Ouse Valley. The most mature soils (podsols, grasslands E, F and G, area NGR TL 762813) showed a parent material of bedded sands with occasional bands of flints and rounded pebbles to depths of 2 m to over 3 m. These appear similar to the Lopham Sands, and are likely to be related to the extensive proglacial lake which occupied the area. Watt [52] noted that the most heavily podsolized soils of the Breckland area he studied appeared to be confined to the low ground under 50 ft (approx. 15 m) O.D., a distribution which is understandable in relation to the ponded lake.

**Figure 5.**
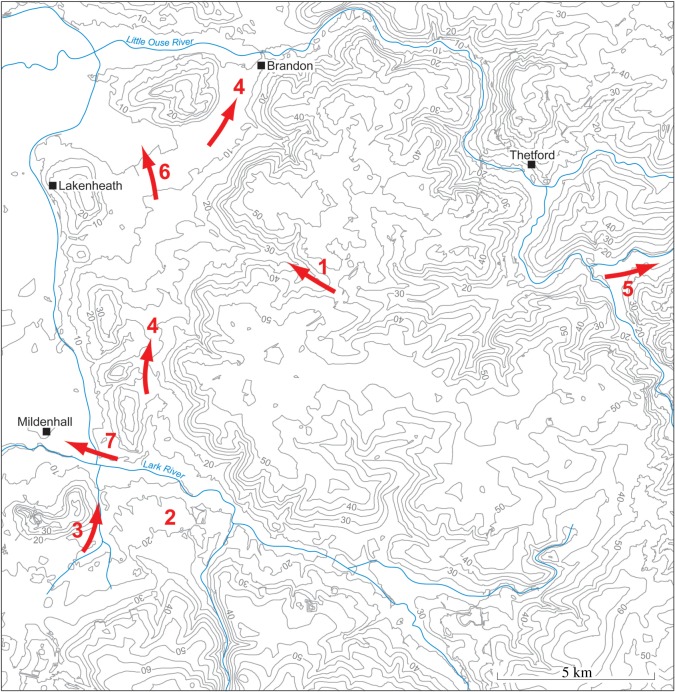
Redrawn map of Breckland by Paterson [32] annotated to indicate drainage of meltwaters at the eastern and southeastern margin of the Tottenhill glaciation. The contours clearly illustrate the isolated hills of the Skertchly Line and their relation to the Anglian till plateau to the east. (1) Former line of the Little Ouse River before capture by the Thet River. (2) Paterson's ‘alluvial cone’ at Cavenham, formed during discharge of meltwaters from the ice front into Lake Paterson, also fed by the Lark River. (3) Ice-marginal drainage south of Barton Hill, from the Kentford area and the Chalk escarpment to the west. (4) Ice-marginal drainage to the east of the Skertchly Line north to the Little Ouse Valley. (5) The exit from Lake Paterson to the North Sea, via the Little Ouse and Waveney valleys. (6) A later stage of drainage to the northwest, following ice retreat, and as Lake Paterson drains. (7) Course of the Lark River on its re-establishment after ice retreat, with dissection of the Cavenham ‘alluvial cone’. The map is described in more detail by West [7].

Paterson's map of the Breckland ([32], described and reproduced by West [7], shows clearly the main features of the landscape beween the Little Ouse and Lark valleys. Figure 5 outlines these features and also shows directions of drainage related to the ice front. The map shows the line of isolated hills above the 100 ft (30.5 m) contour at the eastern and southeastern margins of the Fenland. These features relate to outwash sand and gravels of the Skertchly Line described by Gibbard *et al.* [14–16], the margin of the Wolstonian Tottenhill glaciation of the Fenland, dated to *ca* 160 ka (cf. below). The hills are separated by valleys which increase in size to the north.

To the east of the line of isolated hills is a shallow valley, widening and deepening northwards. The valley separates the hills from the main Chalk escarpment, which rises to a plateau reaching approximately 52 m O.D. There are exits from the valley to the west, between the isolated hills. The exit of the valley to the north is twofold. A wide western branch takes the valley northwest to a downstream part of the Little Ouse Valley in Fenland. A narrower eastern branch takes the valley to an upstream part of the Little Ouse Valley at Brandon.

The course of these valleys suggests that they are related to the overflow history of Lake Paterson, impounded by the glaciation (figure 5). The lake first drained eastwards to the North Sea via the Little Ouse and Waveney valleys [14,50,53]. The narrower eastern branch at the north end of the north–south valley probably formed at this time. With deglaciation, the marginal drainage systems evolved, the eastward direction replaced by a northerly system and a resumption of the Little Ouse catchment and drainage to the west. The enlargement of the chalkland valleys appears to have been greater northwards, towards the Little Ouse Valley, where drainage waters were strengthened by discharge from the lake impounded in the Little Ouse Valley and from its catchment waters.

#### 3.2.5. Lark Valley

The map (figure 5) suggests the presence of an abandoned valley across the chalkland plateau between Barnham and Lakenheath, via the Elveden area. This feature may be related to an original course of the Little Ouse River (with which it lines up) before it was captured by the Thet River. Such a capture was discussed by West [50]. The map shows clearly the abrupt northwards bend of the Little Ouse at Barnham, with the widening of the valley towards Thetford, before the river traverses the Little Ouse ‘gorge’ towards Brandon.

Paterson [32] described an alluvial fan at Cavenham in the Lark Valley (figure 5), shown up clearly by the contours. It has suffered incision by the Lark, by the Tuddenham Mill Stream to the west and by the Cavenham Mill Stream to the east. Its size appears hardly compatible with a sole origin of sediment from the nearby Cavenham Mill Stream Valley. It shows connection to neighbouring extensive gravelly areas, including to the southwest towards Kentford, along a valley now occupied by an under-fit stream, suggesting the presence of marginal drainage of the Fenland ice sheet in the area. Paterson connects the alluvial cone (delta) to an ice-marginal lake with an estimated height of 21 m O.D. The cone appears roughly opposite to the valley described above, indicating a likely connection between the two, with the valley a course of the overflow of Lake Paterson towards the Little Ouse Valley. Later, the drainage altered direction to the northwest following deglaciation, with a course west of the isolated hills.

Apart from Paterson's alluvial fan, lake sediments in the area have been recorded in a few sections. On the south flank of Warren Hill, between Mildenhall and Three Hills, the Beech Clump Pit adjacent to the A11 road showed up to 1.3 m of horizontally bedded sands and interbedded silts [17]. The beds varied in thickness from a few millimetres to 3 cm. These beds indicate the presence of a ponded lake on the southwest flank of Warren Hill. Elsewhere in the Lark Valley, the presence of fine sediments overlying stratified gravels was recorded by Paterson [32] at West Stow School, southeast of Cavenham (Paterson's Site no. 53) and at Rampart Field Gravel Pit (Paterson's site no. 59, Weatherhill Farm Pit). At West Stow School finely bedded yellow sand with clay bands, thickness 12 ft (3.7 m), was recorded above the gravel at approximately 24 m O.D. At Rampart Field Gravel Pit, Weatherhill Farm, lenses of finely laminated clayey silt were recorded above the gravel at approximately 20 m O.D. They were distorted, interpreted as a result of forces associated with the deposition of glacial diamicton above. These sediments may be associated with Lake Paterson, the sequences showing a rise in water level following proglacial gravel deposition in the Lark Valley as ponding developed with the advance of the ice.

#### 3.2.6. Relationships of the lakes

Taken as a whole, these records show similarities in constitution and height of the sands in each area. Whether the lakes are of the same age or related to different episodes of glaciation has yet to be determined.

The Nar Valley is separated from the Wissey catchment by a ridge of chalk, but there appears to be an overflow at Shouldham (18 m O.D.) [16]. The Shingham Lake is in the Wissey catchment, at approximately 20 m O.D. The Wissey catchment is divided from the Little Ouse catchment by a low ridge of chalk, from Feltwell to Wretham. There is no evidence of a marked connection of the Shingham Lake with the Little Ouse Lake and Lake Paterson. The height of the Little Ouse Lake, based on the height of the Lopham Sands, must have reached over approximately 25 m O.D., possibly 30 m when overflow started.

#### 3.2.7. Post-lake drainage

Assuming the interpretation of ice-front recession is correct, it is important to consider the potential role and source of drainage from further west and southwest. Any ice lobe occupying the southern Fenland Basin would not only have dammed the local rivers on the eastern and southeastern basin margins, but also those further to the west, including the Cam, the Great Ouse and the rivers of the Peterborough district. This was already noted by Gibbard *et al*. [15,16]. Assuming those rivers continued to flow throughout the period the Tottenhill glaciation occupied the basin, deglaciation would have been expected to see the rivers reorganizing their courses on the newly deglaciated basin terrain. Initial discharge along or parallel to the ice front is likely to have contributed to and possibly explains the large fan-like accumulations north of Newmarket to the Cavenham fan. However, once the ice had retreated sufficiently to the north, the rivers would be expected to have migrated towards the basin centre. Such a process might explain the substantial distribution of NE-trending gravel and sands to the north and the gravel spread northeast of Fenland (cf. §6.1).

#### 3.2.8. Origin and fate of the proglacial lake sands

The sands are thought to be derived from the Lower Cretaceous Sandringham Sands and Carstone. These beds outcrop at the eastern margin of the Fenland, west of the valleys in which the lakes were impounded by the ice. They are marine in origin, sandy in content [54,55], and are the obvious source of the sand, as suggested by Perrin [56] in his studies of Breckland soils. Worssam & Taylor [57] report a similar relation between the Sandringham Sands and Breckland sand in terms of grain-size analysis. The ice margin in this area indicates that ice passed over the outcrop as it entered the great embayment of the eastern Fenland south of Downham Market, and in doing so set in motion the distribution of sand to the east in the Breckland, via deposition in the proglacial areas, and subsequent redistribution by fluvial and aeolian (katabatic winds) processes into the lakes and the wider landscape. The redistribution in the landscape, under periglacial and aeolian processes, continued in Late Wolstonian and Devensian times to give the cover sands which characterize the Breckland today.

## 4. The southern margin of Fenland

### 4.1. Along the Chalk escarpment

This is likely to have been largely controlled by the height of the Chalk escarpment in the area between the Lark Valley and that of the Cam Valley (figure 1). To the east, the Lark Valley shows ice-marginal accumulations a distance up the valley, as already discussed. To the west, at a distance of 30 km, the limit is reached in the area where the escarpment is replaced by the Gault. Small streams run off the escarpment, draining at the present time into the Cam and Lark rivers. There is a divide on the escarpment formed by a ridge of Chalk projecting towards the Fenland at Hare Park, southwest of Newmarket. To the east, drainage must have been northeastwards towards the Snail and Kennet valleys, tributaries of the Lark. To the west, drainage was to the Cam Valley. Sedimentation at the time of the glacial maximum would arise from deposition of fluvial sediments of these streams, as well as from marginal drainage of meltwaters.

**Figure 6.**
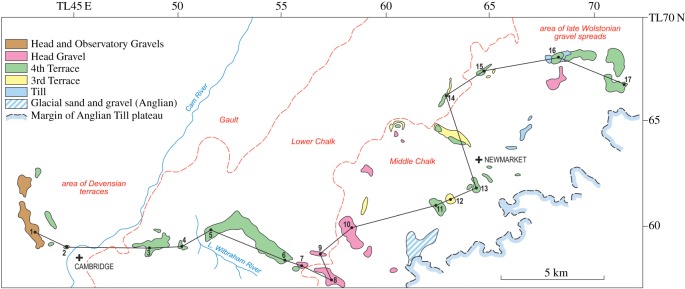
Outline map of the distribution of superficial sediments along the Chalk escarpment between Cambridge and Kentford. The sediments include till, the Observatory and Head Gravels, Head Gravel and 4th and 3rd Terrace gravels. The line of a profile linking sites and heights along the escarpment is shown, the sites numbered as in figure 7. (Based on BGS 1 : 50 000 Sheets 188, 189.)

A variety of superficial sediments, including head gravel and gravels, with a series of terraces of the escarpment streams, are mapped in this area (BGS Sheets 188, 189) (figure 6). Hare Park is a key site for their interpretation and for a recognition of the glacial limit along the escarpment. The geology of this area has been recently reassessed by West [58]. There is an extensive spread of a diamicton, reaching a thickness of nearly 3 m, which is a part of a widespread diamicton of variable composition described by Hodge & Seale [59] in their soil survey of the Cambridge region, and named the Moulton Head after its relationship to the Moulton Complex of soils. The head is a product of periglacial disturbance of the regolith overlying Chalk.

**Figure 7.**
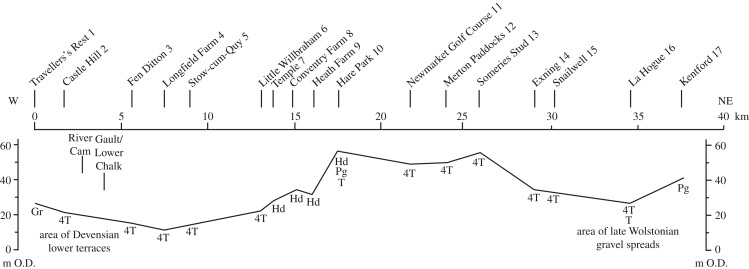
Heights of superficial sediments on the Chalk escarpment between Cambridge and Kentford. The sediments include the Travellers' Rest Pit gravels (Gr), Head Gravel (Hd) and 4th Terrace gravel spreads (4T) on interfluves, proglacial gravels (Pg) and till (T).

An extensive spread of sand and gravel occurs on the summit of the Hare Park Ridge, mapped as ‘Head Gravel’, at a height of 48–58 m O.D. This contains a range of erratics and also remnants of ‘yellowish-brown chalky clay reminiscent of Boulder clay’ (i.e. glacial diamicton) [57]. This spread occurs at a lower elevation than the widespread Anglian Lowestoft Formation till sheet and associated glacial sand and gravel capping the Chalk escarpment a few kilometres to the southeast at approximately 75–90 m O.D. Within the head there are clear horizons of yellow-brown or creamy-brown clay in a number of boreholes in the northern part of the ridge, associated with flint and chalk gravels, which are interpreted as the result of the deposition of melt-out sediments derived from ice, and so associated with structures arising from the abutment of an ice margin against the rising Chalk escarpment at the site. The distribution of boreholes with clay suggests that the ice margin lay close to the west and north of the Hare Park Ridge.

Figure 6 shows the wider distribution of head gravel and gravels along the escarpment, from Kentford in the east to the former Travellers' Rest Pit at Girton in Cambridge to the west. Patches of head gravel are mapped on interfluves both east and west of Hare Park, as are spreads of the 4th Terrace, while the younger terraces follow the streams (BGS Sheet 188). Figure 7 shows the heights of gravel, proglacial gravel, till, head gravel and the 4th Terrace along the escarpment.

The terrace gravels are described in the BGS memoir for the district [57] as flint/chalk gravels, with a variable content of sandy matrix. The 4th Terrace is shown forming extensive spreads of gravels to the east and west of Hare Park. To the east of Hare Park are patches at heights from 45 to 55 m O.D., giving the impression that they are the dissected remnants of a wider sheet, possibly related to the glacial limit. They occur at lower levels west of Hare Park. They contrast with the 3rd and lower terraces which follow the course of the streams draining the escarpment.

Small patches of head gravel and 4th Terrace at 30–45 m O.D. are seen further north of those described above, suggesting a later retreat of the ice front, as indicated by the gravels to the northeast of Newmarket (see below). The two lines are joined by a significant spread of sand and gravel, labelled 3rd and 4th Terraces, associated with the Snail Valley, in the northwest area of Newmarket (NGR TL 630645), which indicates a course of deposition in the Snail Valley, indicating a line of drainage from the higher to the lower line.

The most informative area east of Hare Park is the Kennet Valley and around Kentford, described in detail by Wymer [30], who included in his description the archaeological work of R.J. MacRae. In this area, Wymer notes that palaeoliths ‘have been found in considerable numbers over a wide area and there is evidence that some of them have been swept into glacial outwash gravels, and even more important, this outwash gravel covers a surface on which are palaeoliths little removed, if at all, from their primary context’. This situation parallels that at High Lodge, Mildenhall, where proglacial sediments overlie fine sediments with an *in situ* industry related to a doline [17]. The outwash gravel is at a height of approximately 42 m O.D., distinct from the lower terrace gravels of the area and mapped as 4th Terrace. It overlies in one area approximately 1 m of yellow sandy silt, containing palaeoliths, which is on Chalk. Possibly the silt is associated with a doline, because Wymer [30] described a large infilled depression within the outwash gravels, which he thought might be a kettle hole, but may also have been the result of solution of Chalk below. These observations show the ice margin extended into the Kennet Valley at a height between 40 and 50 m O.D. In doing so, it forced a drainage way between the Kennet and Lark valleys, to the northeast along a channel south of Barton Hill at Herringswell, linking with the area of Cavenham Heath and Paterson's alluvial fan on the south side of the Lark Valley. The proglacial deposits at Barton Hill (Windmill Farm), part of the Skertchly Line [14], lie on the north side of the overflow valley, and so are linked to the ice-marginal drainage system.

To the west of Hare Park, patches of head gravel and 4th Terrace fall in level as the Cam Valley is approached, oriented in part parallel to the Little Wilbraham stream. Head gravel occurs on interfluves near Bottisham at just over 30 m O.D., and an important patch of 4th Terrace forms the ridge of sand and gravel stretching from Little Wilbraham to Stow-cum-Quy. This has a sharp westward bend midway along it, which appears to cause diversion to the west of the Little Wilbraham river, thus enclosing Fulbourn and Teversham Fens. McKenny Hughes [60] noted about this feature that ‘Following the Newmarket Road, we find that the ground rises from the bridge over the stream from Quy Water and a different gravel comes on which is very variable in character consisting in one part of very coarse gravel, and close by of sand or even largely made up of marl and loam’. This feature is complex and is interpreted partly as a drainage way for the stream, and partly for proglacial waters, with the turn west resulting from an ice-marginal position which diverted the stream to the west. The ridge is continued west to 4th Terrace at Fen Ditton and across the Cam to 4th Terrace at Castle Hill in Cambridge. To the north, the 4th Terrace continues east of the Huntingdon Road. Here, and parallel to it and higher on the ridge, are the Travellers' Rest Gravels [61]. These are interpreted as deposits of the River Cam diverted west by the ice at the time of maximum extent of glaciation, in a channel contained to the west by Gault Clay, which was then at a level subsequently lowered by periglacial thermal erosion (see below).

Subsequent to the deposition of the Travellers' Rest Gravels, deposition of 4th Terrace gravels took place as the river course moved eastwards as ice retreat took place. Later erosion of the Gault in the area of the Washpit Brook resulted in the formation of the slope of the Travellers' Rest Pit Ridge, on which the Observatory Gravels were formed (see below).

An important property of the gravels discussed above is their content of reworked palaeoliths, as with the ice-marginal deposits of the Skertchly Line on the eastern side of the Fenland. Thus they are recorded at Kentford, Hare Park and the Travellers' Rest Pit. They are also recorded as scattered finds in areas where patches of gravels are present, as at Bottisham Heath and Kennet [30].

### 4.2. The Cam Valley

**Figure 8.**
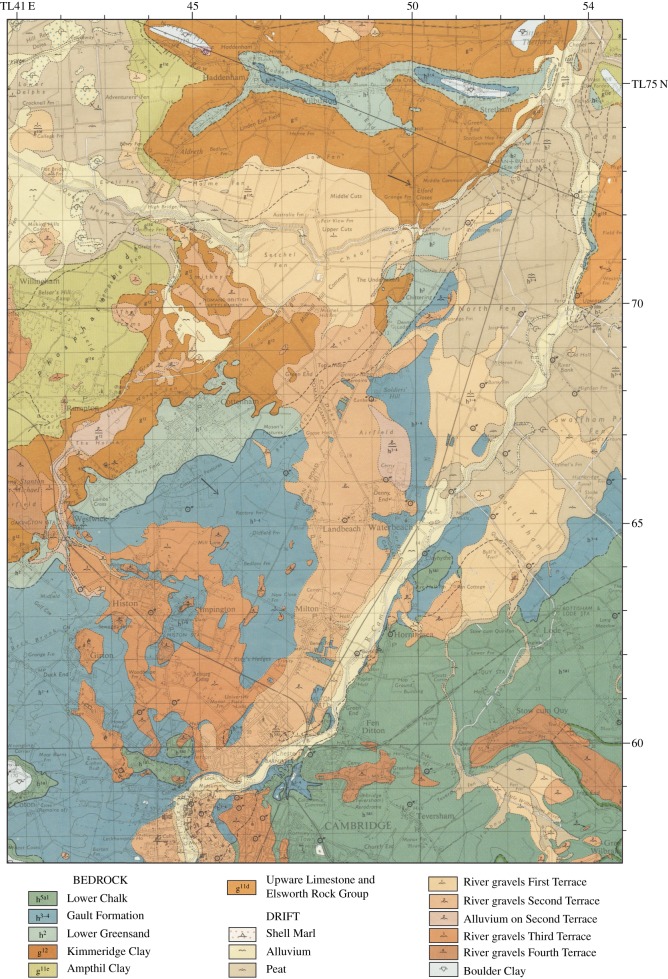
Map of the terraces of the River Cam from Cambridge to the Haddenham ridge, based on British Geological Survey 1 : 50 000 sheet 188 (Cambridge).

The ‘valley gravels’ of the Cam have been described by Worssam & Taylor [57] (figure 8). They are mapped in the BGS Sheet 188 as terraces (4th, 3rd, 2nd and 1st) of descending altitude. Slightly higher than the 4th Terrace are the Travellers' Rest Gravels, mapped as Head Gravel and Observatory Gravels. These, together with the 4th and 3rd Terraces, pass through north Cambridge and continue northwest towards Willingham, to join the River Great Ouse and southern Fenland [62]. The Observatory Gravels occur on sloping ground west of the Travellers' Rest Gravels, and are of a later age. The Travellers' Rest Gravels must have abutted against the Gault Clay bedrock to the east, the level of which has been greatly eroded since by a periglacial stream related to stream drainage to the north to the Great Ouse [61].

The connection with the southern limit of glaciation relies on relating McKenny Hughes' [60] Ridge at Stow-cum-Quy to the 4th Terrace at Fen Ditton and across the Cam to Terrace 4 at Castle Hill in Cambridge (figure 6) [62]. To the north, Terrace 4 continues east of the Huntingdon Road. Here, and parallel to it and higher on the ridge, are the Travellers' Rest Gravels. These are interpreted as deposits of the River Cam diverted west by the ice at the time of maximum extent of glaciation, in a channel contained to the west by Gault Clay, which was then at a level subsequently lowered by periglacial thermal erosion.

These post-Anglian fluvial deposits have been grouped into the Cam Valley Formation. The earliest deposits are Travellers' Rest Gravels and those of the 4th Terrace or Huntingdon Road Member that have yielded mollusc and vertebrate fossils of cool-climate affinities [63,64]. Following a phase of incision, the 3rd Terrace deposits are internally complex including Early Devensian deposits overlying Ipswichian interglacial fine-grained alluvial sediments [65]. At Mannings Farm the interglacial sequence overlies cold-climate gravels of Late Wolstonian age [62]. The Cam 3rd Terrace thus developed in the Ipswichian and the preceding and succeeding cold stages. Significant downcutting followed accumulation of this sequence during the Early Devensian Substage. The 2nd and 1st Terraces developed from the late Early Devensian onwards. Upstream of Huntingdon, the area mapped as Great Ouse 2nd Terrace is internally complex. Subsequent avulsion caused the River Cam to be redirected to the east, towards low-lying areas, to join the River Great Ouse to the north, forming approximately 1 km wide braidplain to the north, identified as the 2nd Terrace west of the present river from Milton to Landbeach and Waterbeach turning NW towards Aldreth from Denny Abbey to join the Great Ouse near Mepal. The 2nd Terrace deposits (Sidgwick Avenue Member) are assigned to the Middle Devensian Substage [66]. At Sidgwick Avenue plant fossils and molluscs from silty and sandy bands within gravel indicate cool to cold-climatic conditions. A radiocarbon date obtained from a *Bison priscus* skull at this site gave an uncalibrated radiocarbon age of 37 746 ± 420 years BP [64].

The younger, River Cam 1st Terrace deposits are of Late Devensian age. This spread occupies a discrete more easterly course than that of the 2nd Terrace. This course was adopted by the river being captured a second time to the east at *ca* 14 ka cal BP, the course breaching a west–east trending ridge. The course took the Cam to the east of Ely and into southern Fenland via the Stretham gap. The 1st Terrace was dated from the Barnwell Station locality, Cambridge, from which Marr & Gardner [67] recovered plant, mollusc and vertebrate remains of cool/cold, full-glacial climate affinities. An uncalibrated radiocarbon age from plant remains here gave 19 500 ± 650 years BP [68] or 23 375 a cal BP, placing the 1st Terrace in the Late Devensian. The Holocene deposits flooring the valley have been classified as part of the Fenland Formation.

### 4.3. The Great Ouse Valley

The Great Ouse terrace deposits' stratigraphy is reminiscent of that from its southern neighbour, the River Cam. The Great Ouse Valley is incised into the glacial deposits underlying the adjacent interfluves to form a series of terrace sequences, grouped as the Ouse Valley Formation [69]. In the lower Great Ouse, four terraces are found. The oldest, the 4th Terrace, is represented by a fragmentary series of poorly preserved gravel patches, By contrast, the lower 3rd Terrace deposits (Biddenham Member) are well-preserved. They consist of gravels and sands, up to 4 m thick, and in places they include temperate molluscan remains in fine-grained strata. These gravels have also yielded Acheulian artefacts [46,70]. Gravelly deposits from the base of gravel aggregations at Galley Hill [71] and Woolpack Farm [72] south of Huntingdon contain fossils suggesting interglacial conditions, but may have been subject to reworking and incorporation in the diamictons and silty layers concerned. More recently, a thick sequence of fine-grained deposits, termed the Mannings Farm Beds, was described within the 3rd Terrace near Willingham, Cambridgeshire [62].

The occurrence of Ipswichian, or reworked Ipswichian deposits in the 3rd Terrace suggests the development of an extensive floodplain on the valley floor, similar to the present floodplains of the River Great Ouse and the Cam. The lower Great Ouse and Cam valleys are areas where Ipswichian deposits have been reported from the Great Ouse 3rd Terrace since the nineteenth century [57,64,65,72,73–80]. With their fragmentary nature these deposits have more recently been placed within either MIS 7 or 9 on the basis of apparently characteristic pollen assemblages, plant macro-remains and faunal indicators (e.g. [64,81]). However, the lack of reliable numerical dating for these periods prevents independent testing of these assertions. Moreover, the reliability of the biostratigraphical indicators employed to reach these interpretations remains questionable in the absence of independent confirmation of their stratigraphical validity. Until these matters have been resolved, the river terrace chronologies continue to provide a firm basis on which to reconstruct the geological evolution of the region.

The altitudinal separation of the Great Ouse 1st and 2nd Terraces is almost impossible, the two accumulations occurring at almost the same height, such that at many places, the two terraces cannot be separated. Instead they are mapped together as the combined 1st–2nd Terrace. On the valley bottom, Holocene or floodplain deposits containing silt and clay with peat occur extensively [62,80].

**Figure 9.**
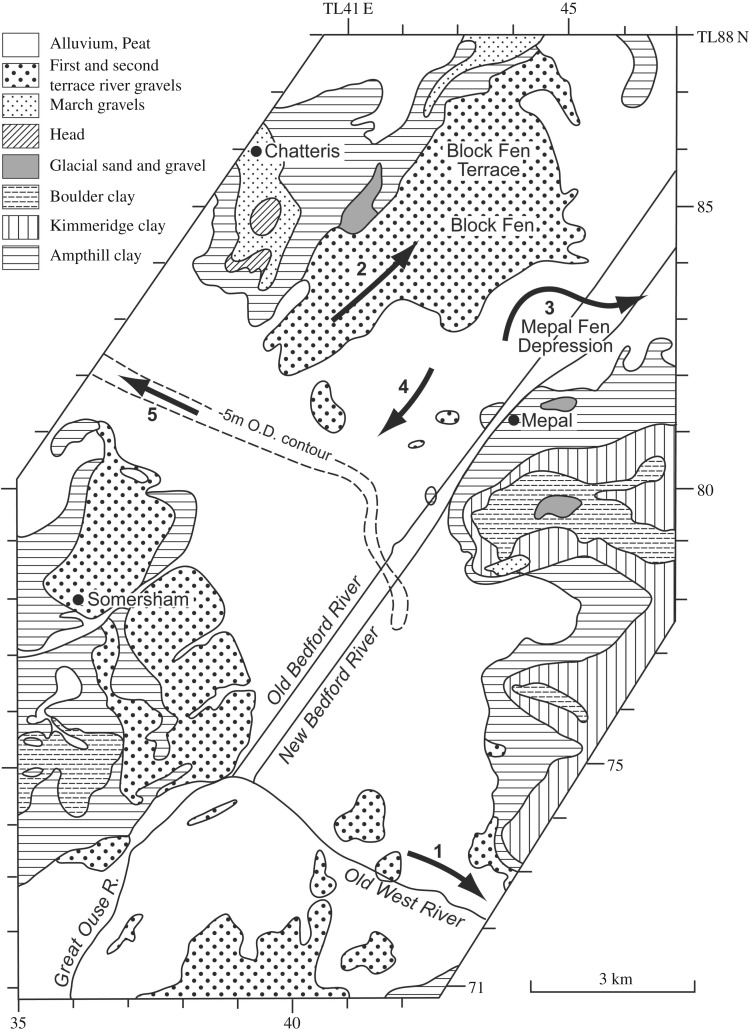
Geological map of the area of diversions of the River Great Ouse between Somersham, Mepal and Chatteris. The −5 m O.D. contour of the pre-Holocene surface is shown, after the map of Waller [3] (cf. figure 15). Arrows mark the flow directions of the River Great Ouse at successive times. (1) Late Wolstonian times in the direction of the Old West (Aldreth) River; (2) Late Wolstonian Block Fen Terrace times; (3) final channel associated with the Block Fen Terrace; (4) reversal of drainage towards the channel west of Chatteris, the West Water of the Holocene. (Based on the British Geological Survey 1 : 50 000 sheets 173 (Ely) and 187 (Huntingdon) and Industrial Mineral Assessment report (IMAU) 124 (Chatteris).)

Of the rivers which enter the southern Fenland, it is the Great Ouse which has been most affected by the glaciation. The northerly course of the river from St Neots to Huntingdon is diverted sharply to the east at Huntingdon, blocked by the till sheet to the north. If this course to the east reflects the approximate position of the ice margin, then it may have continued eastwards in the low valley now occupied by the Old West River, taking the river and associated meltwaters to the area of the gravel spread in southeast Fenland (see below) exposed on deglaciation. On further deglaciation, a later course is indicated by the Late Wolstonian gravel train which is found overlying the Ampthill Clay in the Somersham and Block Fen area, running north in the valley between Chatteris and Mepal [82]. This course became blocked during the Devensian by solifluction associated with the recession of slopes of Ampthill Clay under periglacial conditions [83]. The river was again diverted, this time to the west of the Chatteris ‘island’ to join the Nene River catchment, in the direction of the extinct West Water and Hammond's Eau. Figure 9 shows this sequence of diversions.

## 5. The western margin of Fenland

North of the Great Ouse Valley, the western margin of Fenland abuts a rising plain of Jurassic claylands, overlain extensively by a dissected till (glacial diamicton) sheet. The north-facing slopes show large-scale embayments filled by Holocene deposits of Fenland, including Whittlesea Mere and Holme Fen. To the north of this area, the valley of the River Nene enters Fenland at Peterborough, in an area subject to much recent study.

The Pleistocene sequence in the Peterborough district has been summarized by Booth [84], Horton [85], Davey [86], Langford [87,88] Langford & Briant [89] and Briant [90], together with individual site reports by Horton *et al*. [91], Keen *et al*. [92] and Langford *et al*. [93,94]. In addition, the river Nene terrace succession has been discussed by Castleden [95] and Bridgland *et al*. [96].

As Langford & Briant [89] state, the earliest deposits preserved in the district are the Anglian-age glacial diamictons that underlie the plateau surfaces to the west of the city. This is followed by the Woodston Beds that occur in the south of Peterborough approximately 2 km north of the Norman Cross–Yaxley–Stanground ridge. These fine-grained deposits contain floral and faunal assemblages that represent freshwater to estuarine environments and have been correlated with the Hoxnian interglacial Stage [91]. Locally their base lies at 5.2–10 m O.D. and they occur up to 16 m O.D. [86,89]. They abut the River Nene 3rd Terrace gravels that are aligned SW to NE. The occurrence of these interglacial beds resting on gravel indicates that the Nene Valley was initiated at the end of the preceding Anglian glacial Stage. The Woodston Beds have been correlated with the Nar Valley Clays, both sequences recording high sea level late in the Hoxnian Stage (Substage Ho II–III).

As the River Nene 3rd Terrace gravels are thought to overlie the Woodston Beds, the former must postdate the interglacial, i.e. they must be of Wolstonian age. Indeed Langford [87] considered these gravels to result from glacial meltwater flow through the Southorpe dry valley—linking the Welland and Nene valleys west of Peterborough. The valley was apparently initiated following diversion of the River Welland arising from glacial damming of the river east of Stamford in Lincolnshire. Langford [87,97], HE Langford 2004, unpublished; [89]) considers that the formation of the Southorpe Valley and the fan-delta complex at its mouth at Stibbington and Sutton (west of Peterborough) are coeval and were the result of meltwater streams from the same ice sheet. This point is significant in that it requires post-Anglian glaciation in the western Fenland margin in the Middle Pleistocene. Moreover, glaciolacustrine deposits at Elton in the Nene Valley could also be related to glacial damming of the River Nene. It is significant that Langford ([87,97], HE Langford 2004, unpublished; [89]) concluded that these events represent the same glaciation as that at Tottenhill, Norfolk [12,13].

The interpretation of the Norman Cross–Yaxley–Stanground ridge as a push-moraine, related to the Fenland Tottenhill glaciation ice recession, further strengthens Langford's [87,97], unpublished; [89] conclusion. Assuming this interpretation is correct, it follows that the lacustrine deposits and diamicton underlying the ridge represent proglacial deposition in the Nene basin dammed by ice from the east. This ice ultimately partially overrode the lacustrine sediments to deposit carbonate-rich diamicton and to form the push structure at its margin.

Deglaciation of the district was followed by immediate re-establishment of the drainage system. Following downcutting, the Nene 2nd Terrace gravels were laid down, with similar 2nd terrace deposits occurring in the Welland Valley. These limestone gravels occur along the Nene Valley in the Peterborough area, cutting through the glacial sequence at Stanground. [85]. They include gravel deposits grouped into this unit containing marine Mollusca, Foraminifera and Ostracoda of both cold and temperate aspects. While Langford [87] and Langford & Briant [89] have explained these as representing estuarine sedimentation resulting from a high sea level persisting into a cold period, the more plausible explanation is that these deposits, in common with the March Gravels of the Fenland Basin, result from reworking of older, interglacial materials, in this case potentially from the Woodston Beds. Overall, these coarse arenaceous deposits appear to represent a cold-climate accumulation, predating the Ipswichian Stage. They must therefore also be of Late Wolstonian age, but postdating the Tottenhill glaciation.

Within Fenland the 2nd Terrace gravels fall from 16 m to 5 m O.D., where the March Gravels occur at a similar height. The March Gravels at Eye, near Peterborough, at a height of approximately 1.5–5 m O.D. contain a mixed fauna of marine, brackish, freshwater and terrestrial molluscs, while the Ostracoda show marine, brackish and freshwater affinities [92]. The Foraminifera suggests brackish and temperate conditions obtained during deposition of sands in the sequence. The pollen from clay clasts in the sequence contained a flora including *Carpinus*, suggesting an Ipswichian age of the clast sediment. The conclusion drawn from this study was that the sediments were laid down in brackish water conditions and a temperate environment. But it seems more likely that the mix of environments suggested by the palaeontology is a result of reworking of earlier brackish/marine or freshwater sediments, as at Somersham [98], with the source of the mixture originating in underlying Ipswichian or Hoxnian temperate deposits. The 1st Terrace gravels appear contiguous with the Fenland-edge Abbey Gravels, which, like the March Gravels, contain a marine fauna.

The River Nene 1st Terrace deposits in the Peterborough district are complex, potentially polygenetic and are comparable with the Great Ouse [82,98,99]. Some 1st Terrace deposits of both the rivers Nene and Welland preserve a record of events predominantly during the Devensian Stage, postdating the Ipswichian Stage interglacial [90,100,101].

While the sequence summarized here appears to be coherent, comparable to that in other Fenland and East Anglian lowland river systems, it is important to stress that the re-evalution presented, in part, conflicts with previous schemes (e.g. [89,102,103]). In particular, it raises questions concerning the validity of certain numerical age determinations. While the determinations for the 2nd Terrace materials give ages older than the Tottenhill glaciation, the possibility that the dated fossil material is reworked renders these dates questionable for establishing a chronology of the deposits of this unit.

Horton [85] notes that the ‘river and marine gravels are of broadly similar composition’ with the 2nd Terrace associated with the March Gravels and the 1st Terrace associated with the Abbey Gravels, as described above.

Gallois [2] commenting on the origin of the March Gravels notes that ‘the March Gravels at Wimblington Common and The Dams could be interpreted as shelly patches of river terrace. By analogy, the March Gravels of the March–Wimblington ridge (the type area of the deposit) could be regarded as a fan deposited in a shallow marine bay into which rivers that deposited the higher terraces debauched. This origin for the March Gravels would explain the source of the flints and other stones and their cross-bedding features, and would be in accord with their angularity and lack of sorting.’

The critical question is whether the marine faunas are of a time of deposition of the gravels or are reworked from underlying temperate-stage sediments. The evidence from the Somersham analysis of *in situ* and reworked faunas suggests an alternative explanation: that the fluvial gravels forming the terraces in the Peterborough area and elsewhere in the Fenland dissected underlying temperate deposits, freshwater and brackish/marine, in their restricted valleys as they approached the Fenland Basin, giving rise to the ‘marine’ gravels downstream at the lower levels, thus giving the mixed faunas of the seaward lower terrace deposits.

## 6. Gravel spreads in the area of the Tottenhill glaciation

### 6.1. Northeastern Fenland

A distinct gravel train occurs along the eastern Fenland margin extending northwards from north of Downham Market to Watlington, immediately west of Tottenhill, at the mouth of the Nar Valley. A second patch occurs at Hilgay (figure 3). The deposits abut the higher ground on the eastern side throughout their distribution, their surface forming a distinct terraciform feature declining in the same direction from approximately 9 m to 5 m O.D. at Watlington, its present form including a steep west-facing margin, while its eastern limit forms a gentle gradient with local topography. At its northern end the spread abuts the Tottenhill deposits described by Gibbard *et al*. [13] that occur at 12 m O.D. However, because the terrace-like surface occurs at a distinctly lower elevation (approx. 5–6 m O.D.), it is likely that it was incised into the deltaic accumulation subsequent to deposition of the latter.

These deposits were poorly exposed during the present study, although old workings occur on the east side of the Wimboltsham to Watlington road. The gravel and sand sediments were exposed at Denver Golf Club, adjacent to the Windmill (NGR TF 61724 09272: 7.5 m O.D.), but could be examined in an excavation just south of the Runcton Holme Church (NGR TF 61724 09272: approx. 7.0 O.D.). Here approximately 1.5 m of stratified sands and gravels were exposed (facies Gm, Sp), the uppermost 1 m of which was cryoturbated. Boreholes confirm that the deposits comprise gravel and sand reaching a maximum depth of 3.65 m lying on ‘blue clay’ (Jurassic bedrock, at Plough Lane, Watlington, NGR TF 6254 1094) (BGS), and over 4.0 m in Downham Road, Watlington. Altitudinally equivalent deposits occur north of the mouth of the Nar Valley at Setch, where BGS borehole TF61SW17 (TF6379 1442: 5.47 m O.D.) proved 3.96 m of gravel and sand resting on Nar Valley Formation deposits.

In the past these deposits have been interpreted as representing a terrace accumulation of Fenland rivers [104, p. 91], although the absence of a western valley side puzzled previous investigators. For example, Gallois [105,106] interpreted this spread as a fan deposited into a lake filling the Fenland Basin. However, the form and occurrence of this spread imply that it could be interpreted as a kame-terrace-like accumulation laid down by meltwater flowing northwards marginal to an ice lobe occupying the basin to the west. Whether or not this is correct, its relationship to the Tottenhill sequence demonstrates that the spread postdates the former. This interpretation implies that it almost certainly therefore represents a stillstand position during the local ice recession and that by this time drainage was aligned northwards towards the North Sea. This contrasts with the eastward drainage that occurred during the phase represented by the glacial lacustrine deposits at Tottenhill itself.

As these fluvial deposits occur at 5–6 m O.D. at the mouth of the Nar Valley, they must predate the Wormegay Gravels of the valley, because the terrace surface of the latter occurs at 3.5 m O.D. at the Wormegay stratotype (NGR TF 655131) [107,108]. This observation is important because it demonstrates that there was a significant time interval (and erosional hiatus) between deposition of the marginal Downham Market deposits and the Late Wolstonian Wormegay Gravels. Likewise, there was a similar hiatus between the Wormegay unit and the subsequent Marham Gravel (that directly underlies the Ipswichian-age Pentney deposits [107,108]). In the Wissey Valley, extensive terrace deposits at Wretton, south of West Dereham, at 4 m O.D. are of Devensian age [109].

### 6.2. Southeastern Fenland

**Figure 10.**
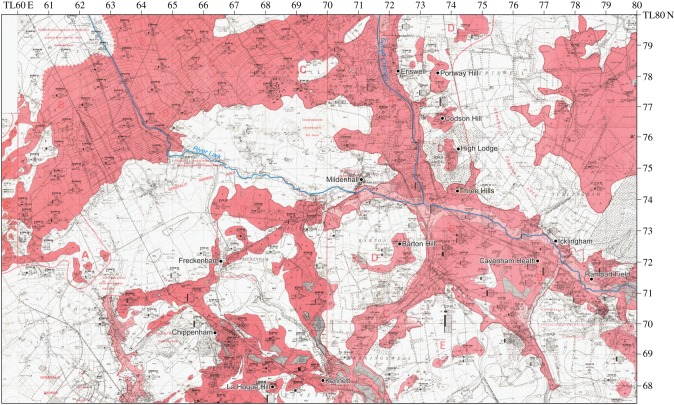
Enlargement of the British Geological Survey Industrial Mineral Assessment Unit (IMAU) Mildenhall sheet TL76, 77, and part of TL 78 showing the gravel spreads of the southeastern Fenland. The dark pink colour represents mineral resources exposed at the surface. Light pink shows continuous or almost continuous spreads of mineral beneath overburden cover.

The distribution of gravel and sand formations in the southeastern Fenland area has been mapped by the BGS [2], and in connection with the Industrial Mineral Assessment Unit (IMAU) programme of the 1970–1980s [110,111]. In the area north of Newmarket and east of Ely, centred on Mildenhall, a series of gravel spreads are mapped (as river terrace deposits) that form distinct, SW–NE trending bodies, linked by NW–SE spreads aligned perpendicular to the bodies (figure 10). Unfortunately, much of this material is not exposed but boreholes put down for various purposes, principally the IMAU survey, provide insight into the disposition of the materials [110,111].

In the area southeast of Chippenham, a large fan-like spread occurs that appears partly continuous with that south of Freckenham, at Red Lodge (T3 of the BGS map Sheet 189). Here, exposures in old workings immediately southeast of the A14 trunk road and B1085 Turnpike Road were examined in 2003 (NGR TL691691). This series of exposures showed stratified gravels and sands up to 4.5 m thick underlain by bedrock Chalk and overlain by approximately 30 cm of sands. The sediments comprise predominantly horizontally stratified sands (facies Sh) interbedded with coarse flint-rich gravel units (facies Gmm, Gms) 20–30 cm thick, the latter with erosional bases grading upwards into the sand units. The gravels are dominated by very large flint clasts up to 10 cm in diameter, with other lithologies being rare, although chalk clasts occur near the base of the unit. At several places individual gravel beds included many vertically aligned stones, a structure associated with cryoturbation. At one site, a small, vertical frost wedge was visible in the gravels. The occurrence of these structures within the beds implies that the frost activity occurred syndepositionally.

According to Corser [110], the gravel fraction comprises 75–80% flint. The higher proportions of flint are found in areas east and south of Chippenham. Both patinated and unpatinated flint occur; the former generally predominates. Unpatinated flint is generally angular to subrounded and includes freshly fractured faces. The patinated flint tends to be rounded to angular. Quartz and quartzite are minor constituents of the gravels, averaging 2%, but ranging from 1 to 6%. Chalk and ironstone clasts occur mainly in the fine gravel fraction.

The available borehole records (e.g. [110]) indicate that the upper surface of these deposits occurs as high as 26 m O.D. in the extreme SE area near the A11 road, but declines in altitude towards the NW to 17 m O.D. west of Freckenham, and 16 m east of the village. Over much of their distribution area, these gravels are no more than 1.5 m thick, but locally, e.g. near Red Lodge, they reach 5.5 m. The SW–NE-trending accumulation from Fordham to Freckenham shows similar or greater thickness up to 10 m. Throughout the area the gravels rest on bedrock Chalk.

North of the Chippenham formation is a second substantial SW–NE-trending spread to the north (figure 10) that extends from Soham to Mildenhall reaching a width of approximately 3.5 km and forms a quasi-continuous feature, although it is not exposed at the surface. Based on the surface altitude, the unit on the south occurs at approximately 3 m O.D. grading broadly NNW to approximately −1 m O.D. towards Prickwillow where the deposits are buried by Holocene Fenland organic materials. The survey map shows this unit divided into two subunits based on surface height, but the distinction is subtle and may not be significant given that the two surfaces are separated by less than 1 m. This gravel and sand unit appears to continue upstream into the Lark Valley in a southeasterly direction where it is mapped as ‘Terrace 2’. The deposits seldom exceed 2–3 m in thickness. In common with the more southerly spreads, the IMAU surveyors [111] describe the gravels as: ‘subangular to patinated flint … typically comprising about 80% of the gravel; chalk, quartz and quartzite are subordinate constituents’, while minor proportions include ‘mudstone, limestone and ferruginous fragments’.

The distribution of these spreads, their form and relation to the local rivers, and finally to previously described units, especially those of the Feltwell Formation, Skertchly Line fan-delta and associated accumulations, suggest that they originate from the deglaciation of the district, because they must postdate the overriding of the area by glacial ice. They also imply that they are not ‘normal’ fluvial accumulations. Indeed the broad SW–NE alignment of the spreads strongly parallels the reconstructed ice-margin direction, i.e. perpendicular to the ice-lobe advance into the region and banked against the gentle southeastern bedrock slope (figures 3 and 10). The distribution of the gravels and sands and the steep, potential ice-contact slopes on their northwestern sides suggest that these accumulations originate from ice-contact meltwater discharge, their gradients aligned broadly towards the NE. Indeed the correspondence of the NW–SE-aligned spreads hints at their origin as material deposited at tunnel portals, like those invoked to explain the Skertchly Line fan-delta bodies and in approximately equivalent positions relative to the ice lobe [14]. The fan-like form of the Chippenham and Red Lodge spreads suggests that they represent subaerial ice-front alluvial fans comparable to that described from Shouldham Thorpe, Norfolk [15,16]. By contrast, the SE–NW gradient of the Soham–Mildenhall spread implies that it might represent confluent fluvial and ice-marginal drainage alignment towards the Fenland Basin centre. This distribution is confirmed by the relationship of this spread to the underlying bedrock topography, the gravels being aligned towards the central Fenland Valley, passing between the Shippea Hill and Littleport ‘islands’ (figure 3).

If these bodies are indeed ice-marginal accumulations, then they appear to indicate that the active ice front was retreating towards the NW (the basin centre), the retreat being punctuated by a series of at least four partial stillstands (figure 10). If this is correct, it is not possible at present to determine how long this might have taken, although the shallow thicknesses of the deposits themselves might imply that any stillstand was short-lived. The decline in surface altitude might reflect source recession during deposition. The easily eroded incoherent bank materials might explain the deposits' shallow thicknesses that allowed the streams to spread laterally largely unrestricted.

### 6.3. The middle level of Fenland

**Figure 11.**
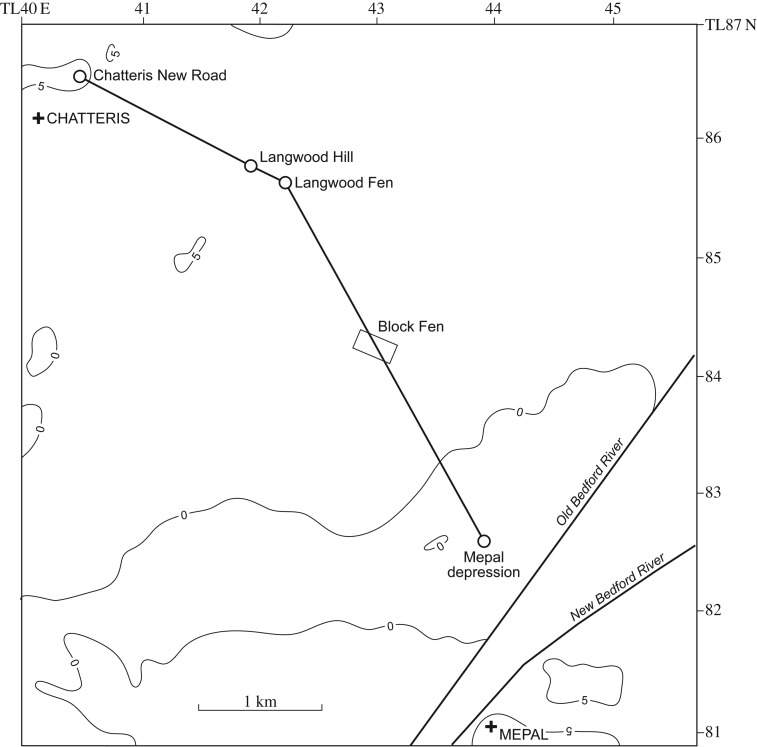
Map of the Middle Level gravels area between Chatteris and the Isle of Ely at Mepal, across the valley once the former course of the River Great Ouse during Late Wolstonian times to an early part of the Devensian, and now occupied by the seventeenth century Old and New Bedford Rivers, which run from Earith to Denver. The figure shows the position of the line of section given in figure 12.

Gravels of the River Great Ouse in Fenland were extensively exposed during the 1970s and 1980s in the area between Chatteris and the Isle of Ely. The exposures of gravel contained horizons of sediments with palaeontology indicating temperate freshwater and brackish/marine conditions, thus splitting the gravel beds. These horizons were correlated with the Ipswichian temperate Stage. The significant sites (figure 11) are in the March–Wimblington–Chatteris area to the west of a former course of the Great Ouse [104], at Somersham on the southern edge of the gravel spread abutting the Ampthill Clay [112], at Block Fen in a central part of the former course of the river [99], and near Mepal, also in the central part of the former course of the river [83], abutting the Ampthill Clay on the east (Isle of Ely) side of the former river course.

**Figure 12.**
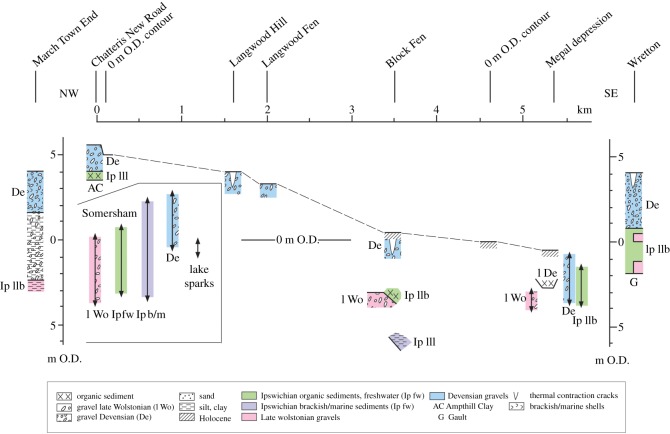
Section across the valley between Chatteris and Mepal. The main part of the figure is the section across the valley between Chatteris and Mepal, shown with the height relationships of Late Wolstonian (lWo), Ipswichian (Ip) and Devensian (De) stage sediments. Sections at March Town End (to left) and at Wretton (to right) are shown for comparison. The main section includes an inset showing the range of heights of Late Wolstonian, Ipswichian (freshwater and brackish/marine) and Devensian-age sediments, including those of Lake Sparks, at Somersham. The range of heights in the Mepal depression are indicated in the same way, together with the height of Late Devensian organic sediments in the depression. Subdivisions of the Ipswichian Stage are based on palynology.

Figure 12 presents a cross section across the former course of the Great Ouse from Chatteris to Mepal, also giving heights of relevant sediments at March, Somersham and at Wretton near Stoke Ferry in the Wissey Valley at the eastern margin of Fenland. The cross section shows the eastwards descending height of the gravel surfaces as the centre of the former course is reached at Mepal. O.D. heights are shown of the pre-Ipswichian (Late Wolstonian) fluviatile gravels, Ipswichian freshwater sediments, Ipswichian brackish/marine sediments and Devensian gravels, with associated sediments in the Mepal depression. The following units are present:

*Late Wolstonian gravels*. These gravels, postdating the Tottenhill glaciation of the Fenland, are recorded at Somersham (−0.4 to −2.5 m O.D.), and in the Mepal depression (−4.0 m to −2.5 m O.D.).
*Ipswichian freshwater sediments*. These are recorded at Somersham (−3.5 m to 1.0 m O.D.), and in the Mepal depression (−4.0 to −1.0 m O.D.).
*Ipswichian brackish/marine sediments*. These are recorded at Somersham (−3.7 to −0.5 m O.D.), at Block Fen (−6.3 to −5.8 m O.D.) and at March (−2.5 to 2.4 m O.D.).
*Devensian gravels and associated sediments.* These sediments are recorded at Somersham (−4.0 to 0.5 m O.D.), at Block Fen (−2.0 to 4.0  O.D.), in the Mepal depression (to a height of −0.5  O.D.) and at March, the ‘type site’ of the March Gravels [113] (to a height of 5 m O.D.). There are Devensian solifluction sediments and Devensian late-glacial organic deposits in the Mepal depression which demonstrate a complex history of the former valley of the River Great Ouse, which resulted in the diversion of the river westwards of Chatteris in the later Devensian [83], as described above.

This analysis shows that Late Wolstonian gravel spreads in the Fenland suffered incision prior to the Ipswichian marine transgression, and that the levels of all the above-mentioned units overlapped during the time represented, within a vertical band of some 11 m. The spread of Devensian fluviatile sediments in the area, overlying Ipswichian freshwater and brackish/marine sediments, covers a surface level, declining from 5 m O.D. to near O.D. towards the present valleys and reflecting a lowering of sea level during the Late Wolstonian. The terraces are indistinct, shown by small areas which are relatively level (e.g. Wimblington Common, Lingwood Hill).

In some areas, the gravels contain floras and faunas reworked from the underlying Ipswichian deposits, such as at Somersham [98] and March [82]. These have often been described as a distinct stratigraphical unit, the March Gravels of Baden-Powell [113]. But they are better considered as a local variant of the Devensian suite of gravels containing reworked marine fauna from underlying earlier temperate-stage deposits, a process analysed and demonstrated at Somersham [98].

The Somersham site is particularly interesting in showing a brackish/marine influence extended to the southern margin of Fenland in the Ipswichian, to a height of −0.5 m O.D. This transgression must have affected all the major rivers of the Fenland at the time. The site also contained varved sediments of the Devensian proglacial Lake Sparks [114], indicating the presence of what must have once been a widespread lake in the Fenland, for which there is little evidence preserved.

## 7. Fenland ‘islands’, ridges and meres

In the BGS memoir Gallois [2] for the Ely sheet (173) comments ‘one of the unsolved mysteries of Fenland is the origin of the ‘islands’ that rise above the Recent deposits' (‘Recent’ = Holocene). These ‘islands’ occur as isolated, low hill-like ridges that stand above the low plain of the Fenland Basin. Although complex in plan, the ridges are distinctly elongated in a WNW to NW–SE to ESE direction, forming a series of subparallel features. As Gallois [2] notes, these characteristic elongate forms have distinct marginal slopes which are ‘commonly 5–7°, the ridges rising from 0 m a.s.l. to over 20 m’. He considered that the slopes are in places modified by fluvial activity (e.g. the western side of the March–Wimblington ridge and the eastern slope of the Ely–Littleport ridge); elsewhere there is no evidence that these landforms were substantially modified by later action. There is a tendency for the slopes to be steeper on the south-facing sides. In several places, the areas broadly immediately to the north or NNW to NNE of the islands show minor basinal depression-like forms to the bedrock surface. These hollows do not occur on the opposite side of the features, e.g. Grunty Fen (figure 3). The depressions are largely infilled by Fenland Holocene sediments and include the meres of the southern margin of the Fenland (Whittlesey, Soham, etc.).

In terms of their geology, exposure on the islands is limited. Predominantly the ridges, such as the March–Wimblington complex, Manea, Little Downham, Witchford, Stretham, Wicken and Shippea Hill, are mapped as bedrock-cored and capped by glacial diamicton (the ‘Chalky-Jurassic till’ in [2]), generally less than 10 m thick, but locally considerably more. This sequence is confirmed by borehole evidence (http://mapapps.bgs.ac.uk/geologyofbritain/home.html). According to these records and Gallois [2], the diamicton is brown in colour, has a sandy clay matrix and includes erratics of chalk, flint and Mesozoic lithologies, notably Triassic marl, red chalk, Carboniferous limestone, and a few far-travelled igneous and metamorphic rocks. Most of the matrix is derived from local Jurassic clays. Gallois [2] concludes that the diamicton material was derived from the northwest or north, but he notes that the high frequency of Triassic rocks in the basal parts of some sections implies a possible varying ice-movement direction. However, this could also indicate incomplete incorporation and mixing of locally reworked source materials.

The lithological similarity of this diamicton (tentatively termed here the Feltwell Formation Roslyn Diamicton Member—stratotype Roslyn Hole pit, Ely) to the characteristic Anglian-age Lowestoft Formation diamicton (Chalky Till) implies that the latter probably provided considerable source material for the younger unit. If this is the case, differentiation of the two diamicton units, in the absence of intervening stratigraphic markers, could prove problematic (particularly in boreholes). Such a situation might explain the considerable thicknesses of diamicton (over 60 m: TL27NW153, NGR TL523660) encountered in the Huntingdon district, for example.

Gallois [2] notes that, in places, the diamicton is overlain by gravel and sand, in part cryoturbated at Stonea (NGR TL 4506 9449), a similar sequence being recorded at Mill Lane, Littleport (NGR TL 5649 8634) and a few other sites. Occasional localities also include laminated clay strata intercalated in the diamicton member. Little more can be said about the general pattern, because of the limited exposure; however, one locality provides a unique picture of the internal structure of the Ely Ridge.

**Figure 13.**
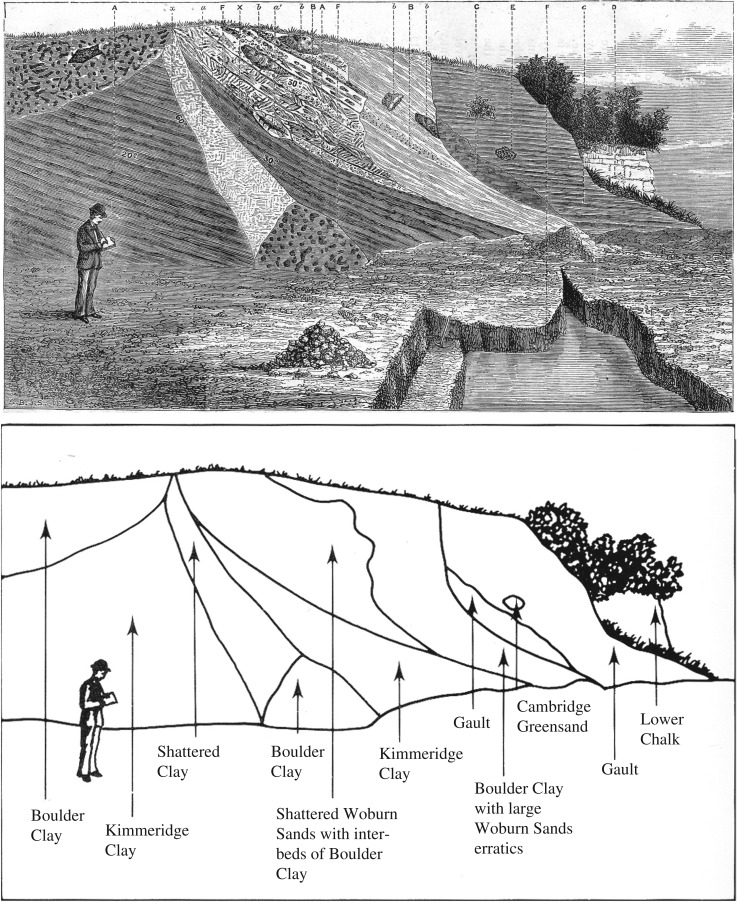
Woodcut image of the section at the Roslyn Hole pit, Ely by Skertchly [5] showing the ‘Great Erratic’, and below an interpretation by Gallois [2] from the British Geological Survey 1 : 50 000 sheet 173 (Ely) memoir.

The BGS borehole archive (http://mapapps.bgs.ac.uk/geologyofbritain/home.html) includes records from the Ely–Littleport Ridge. In central Ely, borehole TL57NW44 (NGR TL 5533 2799: 22.5 m O.D.) at the RAF Hospital recorded 4.0 m of ‘greenish-brown’ diamicton overlying Kimmeridge Clay bedrock, a similar sequence being encountered at Barton Farm (TL57NW33: NGR TL 5538 2796: 14.5 m O.D.) where the ‘yellowish-brown’ diamicton was 2.0 m thick. However, an excavation for material to repair river channel embankments provided extensive exposure in the Roslyn Hole pit (NGR TL 557 807) on the eastern side of the Ely–Littleport island complex. Here, sections observed from the mid-nineteenth century demonstrated the occurrence of a large erratic floe-block of bedrock within the till (figure 13). Skertchly [5] records the ‘Great Erratic’ as 50 × 400 m in plan and over 5 m thick. It comprised a sequence of Kimmeridge Clay, Lower Greensand (Woburn Sands), Gault Clay, Upper (Cambridge) Greensand and Chalk dipping at an angle of over 30°. The interpretation of this sequence of bedrock materials has been controversial since its original description in [115]. However, Fisher [116] and Bonney [117] concluded that the erratic must have been emplaced by glacial action. This interpretation was subsequently agreed by Skertchly [5, p. 236] who observed diamicton surrounding the block on all sides (figure 13). This specific assemblage of bedrock lithologies can only have been derived locally from the west or northwest in the Ely–Littleport area, the floe-block having been deposited in the lee of the island ridge (cf. [2]). The emplacement, as well as the high angle of dip and slickensided margins between the bedrock contacts [117,118], suggests the block was glaciotectonically thrust. Thrust-block moraines are typical of surging glacier land systems [119], and this possibility is explored further below.

Taking this evidence together, it is evident that the ridges predate the formation of the modern Fenland Basin. Gallois [2] thought that they represented islands formed in a lake or the sea, the implication being that they are eroded remnants of a once continuous cover. If this were the case, it does not explain why they are clearly associated with a glaciation that traversed the area from the N–NNW, a direction already identified from the earlier evidence presented above. Indeed this direction corresponds closely to that observed from the sorted gravel and sand formations to the southeast, as well as that from the Skertchly Line and associated sequences from the eastern Fenland margin. That the island ridges form a series of subparallel features aligned broadly WNW to NW–SE to ESE suggests that they originated as landforms marginal to the ice sheet, their situation implying that they were formed during stillstands in the retreat of the ice lobe.

Although there is only one exposure indicating unequivocal glaciotectonic activity associated with the ridges in the central Fenland, comparison of the form and distribution of the ‘island’ ridges strongly suggests that they all represent ‘hill-hole pair’ (ice-pushed ridge) glaciotectonic landforms (cf. [120]). The Fenland ridges are characterized by an arcuate-shaped plan morphology on the proximal sides, steep marginal slopes on the distal sides, subparallel ridge-alignment, their internal geology and the occurrence of basinal depressions on the potential (concave) up-glacier side of the ridges. Finally, the occurrence of meltwater deposits on the tops of the ridges has been recorded from comparable landforms by Bluemle & Clayton [121] and implies that the gravels and sands, and the lacustrine deposits on the ‘islands’ can also be explained by local glacial meltwater release.

**Figure 14.**
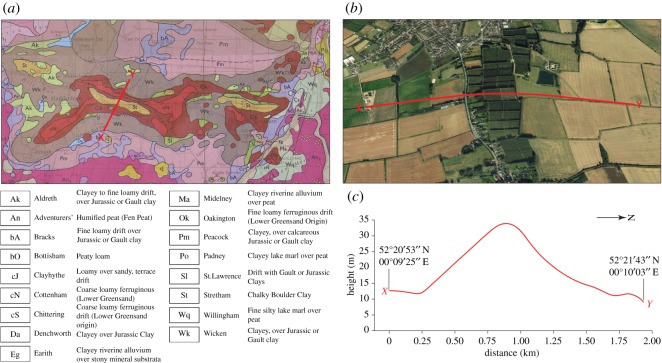
Haddenham Ridge. (*a*) Extract from Soil Survey map 1 : 50 000 sheets 173 (Ely) and 188 (Cambridge). (*b*) Oblique aerial photograph of the ‘island’ ridge at Haddenham looking towards the west and (*c*) a topographic cross section *X–Y* showing the surface form of the ridge (source: Google Earth).

In addition to these observations, it is notable that the Fenland ridges are restricted to areas of Mesozoic clay substrate: the Littleport–Haddenham complex overlying Kimmeridge Clay, the March–Manea group overlying Ampthill Clay, the Stretham–Wicken ridge overlying Gault Clay and those to the west rest on Oxford Clay. By contrast, the ridges are absent from those areas underlain by Chalk and Greensand rocks (figure 3). This distribution cannot be coincidental. Indeed descriptions of composite ice-pushed ridges of the form and scale of those in the Fenland have frequently been recorded from areas underlain by clay bedrock, e.g. in northern Jylland, Denmark by Gry [122] and north Germany [120]. Ice-pushed ridges of this type are well known from the central Netherlands where they are more composite in plan, having been formed by thrusting of unconsolidated Rhine–Maas fluvial deposits [123,124] to produce the characteristic ridge-and-basin topography. Figure 14 illustrates the geology and form of such a ridge at Haddenham (figure 1).

The interplay of a range of factors that influence their formation includes the nature of the substrate materials, inhibited meltwater release and the potential occurrence of permafrost (cf. [125]), each of which could have conceivably been instrumental in the formation of the Fenland ridges operating either independently or in concert.

The Fenland ice lobe had a very low gradient with little variation in altitude along its lateral margins, based on its topographic expression. Such a low gradient means that the shear stresses associated with the ice lobe would have been very low (cf. [126]). The extensive advance of the Fenland ice lobe must have been facilitated by a highly deformable bed or subglacial meltwater, or both. If the driving stress from ice is less than the yield stress of the subglacial bed, no ice movement can occur under perfectly plastic ice rheology. Conversely, if the driving stress from ice is greater than the yield stress of the subglacial bed, then the ice will simply adjust its profile and depth accordingly until the driving stress matches the yield stress [127]. This means that the substrate in the Fenland Basin must have a low yield stress to explain the palaeoglaciology of the area. Indeed, the basin is underlain by soft Mesozoic clays which would have been easily deformed by the advancing ice, enabling the ice lobe to extend further south than ice outside of the Fenland Basin. The clays would have had low ductile strength compared with bedrock lithologies surrounding the Fenland Basin and this explains the geometry of the lobe. Very low gradient lobes are also facilitated by subglacial floods [128] and the evidence of ponding and ice-marginal lakes within the Fenland Basin [14] would have also magnified the effects of a highly deformable bed substrate. This would have also facilitated sliding, contributing to glacier surging. This is especially because the subglacial water would have filled the entire Fenland Basin, reducing the effective pressure at the base of the ice sheet, and facilitating increased glacier velocities through the ice lobe (cf. [129]). All of these factors are consistent with surging glaciers. Furthermore, the evidence of severe permafrost conditions in the region in the Wolstonian [14,22] may have meant the ice lobe was polythermal and thus potentially cold-based at the margins yet warm-based over large areas under the ice lobe, which again would have made it prone to surging (cf. [130]).

The interpretation of the Fenland ‘islands’ as ice-pushed ridges implies that they represent active ice-front stillstands or minor re-advances associated with periodic surging into the basin. If this is correct, they indicate a series of four or possibly five such events. These stillstand limits are also apparently represented by the meltwater spreads identified from the Mildenhall–Newmarket area, where possibly as many as four positions are indicated (see above). Combining these limits with those identified above suggests a minimum total of six, or possibly seven, potential positions. Indeed the apparent eastward continuation of the Haddenham–Stretham–Wicken ridge to the northern limit of the Soham–Mildenhall gravel and sand formation (figure 10) implies that the latter represents an equivalent stillstand position. The maximum extent may have been even further, possibly reaching the northern extremity of Cottenham, and continuing westwards to Over and thence northwestwards to Bluntisham.

The Great Ouse enters Fenland near Over, where there is a ridge of till and glacial gravel at 10–15 m O.D., related to the till ridges described below. On either side of the valley west of Over are spreads of gravel of the 3rd Terrace of the Great Ouse at approximately 11 m O.D. Otherwise the valley from here to Huntingdon and south to St Neots is filled with a broad expanse of 1st and 2nd Terrace gravels. Gravels of the 3rd Terrace at Fen Drayton (IMAU borehole TL 36 NW 16) overlie organic sediments at the same height as Ipswichian sediments at Chatteris (3–5 m O.D.), suggesting that the 3rd Terrace is Devensian in age. The wide expanse of gravels in the valley from Over upstream suggests that the valley was ponded by the ice of the Tottenhill glaciation, with its margin on the north side of the valley and to the east. There is no record of quietwater sediments in the valley which might indicate ponding, and it appears that the fluvial system remained actively draining the area during the events relating to the Great Ouse at the time of glaciation.

A short distance to the east of Over are 3rd and 4th Terrace gravels of the Cam Valley at approximately 10–15 m O.D., suggesting a complex drainage system in the area involving events at the glacial margin of both Great Ouse and Cam Rivers during the glaciation and the deglaciation.

The westward projection of the Haddenham ridge (noted above) suggests that it continues north of St Ives to the Bluntisham–Woodhurst interfluve, while the Ely–Sutton ridge can be traced to the Pidley interfluve, west of Somersham (figure 3). Tentative projection of the ridge alignments further to the northwest suggests that they continue broadly to Wenningham and Warboys, respectively, where they merge with the thick mass of diamicton north of Huntingdon. From here they can potentially be traced, after a break on the Folksworth interfluve, where they turn at right angles, to continue as the substantial ridge reaching to over 30 m O.D. through Yaxley to north of Farcet, southeast of Peterborough. Langford [88,97] notes that the internal structure of the ridge is complex, including meltwater sands interbedded with two to three diamicton units and associated bedrock inclusions (e.g. adjacent to the A1 Motorway at Norman Cross). Southeast of Peterborough, BGS boreholes and the Ramsay 50 000 map (sheet 172) indicate that the diamicton here rests on glaciolacustrine deposits, and is locally overlain by gravel and sand. Of particular importance is the temporarily exposed sequence described from Stanground (NGR TL 202955) by Langford [87,88,97,131]; HE Langford 2004, unpublished. Here what he interprets as 7.5 m thick ‘deltaic sands and gravels with interdigitated debris-flow deposits’, overlain by bottomset fines (up to 10 m thick), in turn overlain by up to 7 m of ‘chalk-rich, structureless diamicton’ (possibly resembling ‘marly drift’), the latter possibly deposited as a subaquatic ‘slide facies’, occur. His description of the diamicton, includes an ‘irregular, lower bounding contact; ribbons of the subaqueous slide material descending into the underlying deep-water facies’, and notably ‘thrust faults associated with a synclinal and overturned anticlinal pattern of deformation’ including ‘pockets trailing in the direction of transport’, and together rather than implying a gravity slide origin for this diamicton unit, the evidence suggests it represents a glaciotectonic thrust, analogous to those seen at Ely and other East Fenland margin sites, e.g. High Lodge, Warren Hill, etc. described above.

According to Horton *et al*. [85,132], the lacustrine deposits comprise thinly bedded brown and grey clays up to 15 m thickness near Farcet filling a shallow, bedrock-floored depression at approximately 5–10 m O.D. The Stanground delta was presumably also laid down into the same glacially dammed lake at the adjacent ice margin to the northeast at the basin's eastern extremity [88,135–131]. It is interesting to note that the substantial depression, occupied by the Whittlesey Mere and Ugg Mere basins, occurs within the semicircular, concave arc of this Yaxley to Wennington (push-moraine) ridge complex (figure 3).

Further examination of the grouping of the ridges suggests that they represent a double-lobed ice-front system, an eastern lobe that occupied the Mildenhall and Ely area, and a western lobe in the district to the west of the line from Littleport to Stretham, the NE–SW-orientated Ely–Littleport Ridge apparently occupying an interlobate position. At their maximum extent, the western lobe extended as far as Sawtry and Stilton, south of Peterborough, while the eastern lobe extended to the Skertchly Line at Barton Hill and the Lark Valley sites (figure 10).

## 8. Digital terrain model

**Figure 15.**
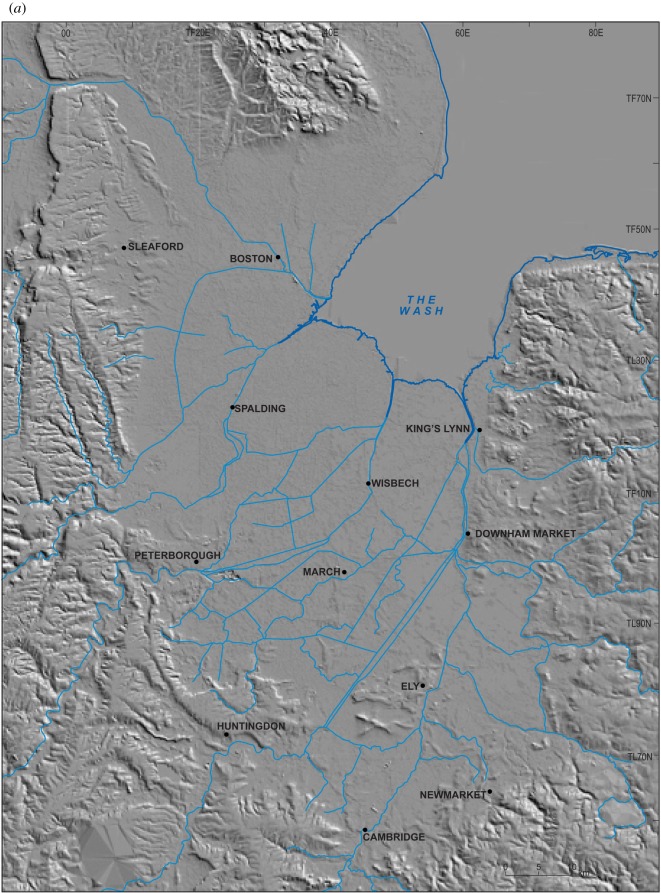

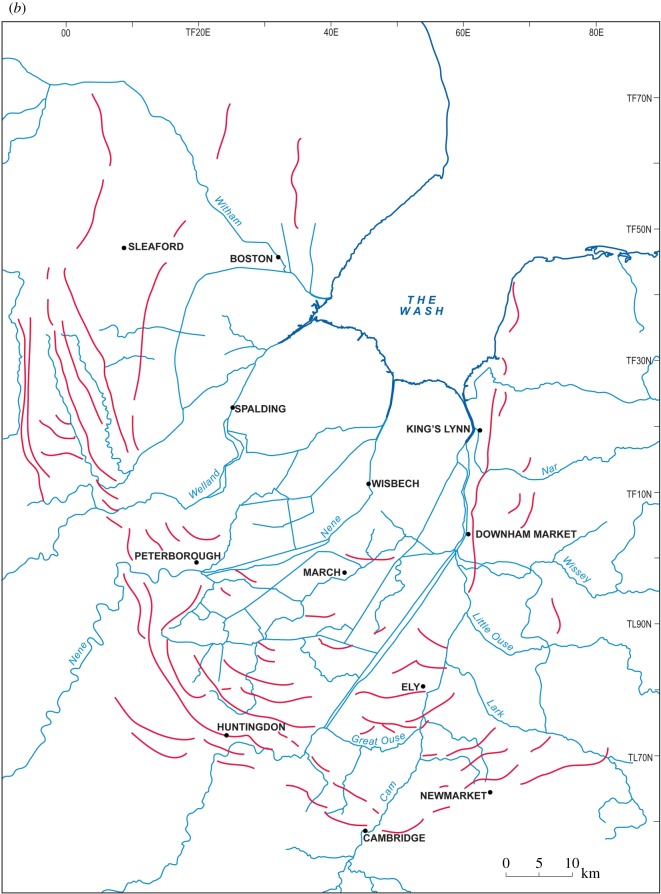
(*a*) Digital Terrain Model (DTM) of the Fenland Basin constructed from a 50 m cell size, as is the hillshade generated from it. The hillshade is at an azimuth of 045° and an altitude of 45°. (*b*) Interpretation of the landforms shown in the DTM. The red lines indicate the ridges interpreted as glacio-marginal push features.

The interpretation of the Fenland ‘islands’ and associated ridges as marginal push-moraine complexes is further reinforced by inspection of a digital terrain model (DTM) of the basin. Figure 15*a* shows this model constructed from a 50 m cell size, as is the hillshade generated from it. The hillshade is at an azimuth 045° and an altitude of 45°. It is accompanied by an interpretation of the landforms (figure 15*b*). This interpretation unequivocally demonstrates that the subparallel, quasi-continuous, ridge-like hills are aligned in a pattern that reflects a substantial lobate form filling the Fenland Basin. The ridges can be seen to extend from the areas described above via Peterborough north to between Grantham and Sleaford in Lincolnshire, and southwards to north of Cambridge, from where they turn eastwards to link with those already described bordering the south and southeastern Fenland. Comparison of the form and distribution of the ridges implies that they are marginal glaciotectonic pushed hills and associated structures, the maximum extent of the lobe being approximately 86 km north–south, and approximately 46 km east–west in the north and approximately 52 km east–west in the south.

Comparison of the landform assemblages with those of other similar lobate glacial basins (e.g. the Wisconsinan-age Green Bay lobe in North America (cf. [120,126])), though of smaller scale, places beyond doubt this interpretation of Fenland Basin's glacial origin, the overall alignment of landscape forms indicating the advance of the Tottenhill ice lobe from the north to northeast. Major ice lobes of the last Laurentide Ice Sheet were often associated with a slowly permeable substrate and low-gradient lateral margins [133], like those observed in the Fenland Basin. A similar explanation was proposed by Tveranger *et al*. [134] for the lobate margin of the northern Russian Kara Sea Ice Sheet for which a deformable bed has been invoked. This suggests that the southern part of the Kara Sea Ice Sheet, in common with those in North America, may have undergone marked and rapid fluctuations, implying that they occurred as short-lived surges of thin and fast-moving ice. As already noted, this situation is typical of surging ice lobes, especially when combined with subglacial flooding, arising from inhibited drainage (cf. [128]). Surging glaciers often produce ice-pushed or ice-thrust ridges [135], and this is also consistent with the ridges observed in the high-resolution DTM of the Fenland Basin. This is also supported by the evidence of glaciotectonism in the morainic sediments, and the overall sediment–landform associations are consistent with a surging glacier land system [119].

## 9. Bedrock topography and extension into the Wash

**Figure 16.**
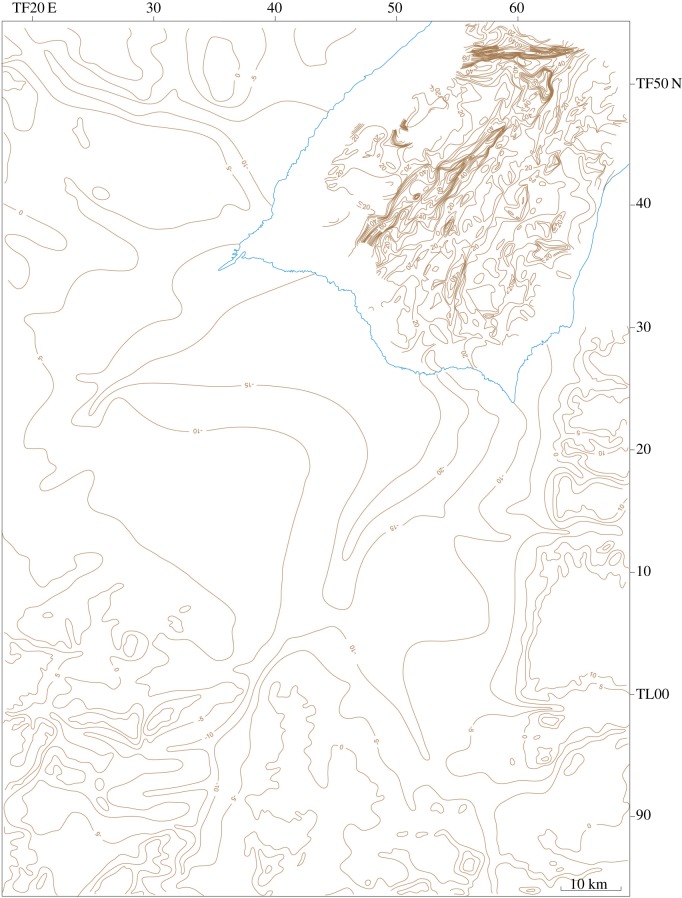
Bedrock surface of Fenland constructed by Waller [3], contour interval 5 m and British Geological Survey bedrock surface of the Wash (from [3]) inserted, submarine contour interval 4 m.

Examination of the bedrock topographic map [3] (figure 16) shows a number of important features that are not expressed in the present surface topography. Of particular importance is the northward-aligned valley that occupies the central northern half of the basin, immediately south of the Wash. This basinal depression, once vacated by the ice lobe, would have been reoccupied by the rivers. A combination of glacial erosion and potential isostatic depression of the region, centred on the central North Sea Basin, would have contributed to the alignment of the streams towards what is now the Wash and the southern North Sea, off-northern East Anglia. Tracing these valleys upstream, they divide north of the March Island, with the main (eastern) valley continuing from Welney to Littleport and thence turning SSE towards the Cam spreads north of Cambridge. This valley is also joined on its eastern side by the eastern Fenland margin rivers, the Lark, Little Ouse, the Wissey and further north, the Nar. The smaller western valley continues to the west and southwest of Chatteris and appears to be an extension of the Great Ouse. However, the extensive gravel spreads occupying the low area between the Chatteris and Sutton and further north between Manea and the Littleport ridges were exposed at Block Fen [82]. They unequivocally indicate that the Great Ouse was earlier aligned NE through this topographic low, to join the Cam in the Welney area until after the Middle Devensian (as described below). This implies that the western valley, now linked to the Great Ouse, is a younger feature, the river having abandoned the Block Fen course, as described above.

Examination of the bedrock surface and geological maps of the floor of the Wash indicate a continuation of the features observed beneath Fenland (figure 16). The substantial valley below −20 m.a.s.l. can be seen to continue as the Lynn Deep which grades seawards towards the NNE [136]. To the west is a subparallel valley today occupied by the Boston Deep, which is floored by gravel. This valley rests on glacial deposits within a substantial bedrock valley, over 60 m deep. This partially filled tunnel valley grades northeastwards into the channel system entering the Silver Pit, off the coast of northern Lincolnshire (cf. [136,137]). The Silver Pit tunnel valley, like those on land and elsewhere beneath the southern North Sea appears to be of Anglian (Elsterian) age, being infilled by Svarte Bank Formation sediments that include Hoxnian Stage marine deposits [138,139]. Reoccupation of partially filled tunnel valleys by later river systems, e.g. the Waveney, Hitchin-Stevenage and Nar valleys, the infilling of the latter also including Hoxnian interglacial marine sediments (cf. [140], above), is analogous to those recovered from the Silver Pit [139].

A third deep valley aligned east–west at the mouth of the Wash (figure 16) joins the Boston Deep Valley, this orientation suggesting a trend towards the Lincoln Gap to the west. Attempts to trace this valley on land immediately west of the Wash, based on examination of borehole records, was unsuccessful. Here a till cover over 10 m thick rests on bedrock. However, the possibility remains that the valley continues on land.

Those rivers that formed following the Anglian deglaciation of the basin would have probably followed courses through the depression controlled by the incompletely filled tunnel valleys. Following the Anglian glaciation, the only available exit to the North Sea towards the northeast, was the Wash. The Wash gap certainly existed in some form at the end of the Anglian glaciation because Hoxnian-age marine deposits occur in the Nar Valley, near Kings Lynn, Norfolk, and at Peterborough, Cambridgeshire, showing that the northern Fenland area was open to the sea at this time, implying an estuary may have existed here (cf. [89]).

Secondly, if the ‘proto-Soar/Bytham’ stream is correctly identified as having adopted a course eastwards through the South Witham Gap in the Jurassic escarpment in the post-Anglian period (cf. [137]), the river would have followed the course south of Spalding, Lincolnshire, crossing the northern Fenland/Wash region to enter the North Sea via the Wash tunnel valley during sea-level low-stand phases of the Wolstonian Stage. Likewise the rivers of the northwestern Fenland margin (Welland, Nene, West Glen) would be expected to have been confluent with this stream.

Thirdly, because this south Lincolnshire–Wash course must have been overridden by the subsequent ‘Tottenhill ice sheet’ as it advanced into the Fenland, the gravel braidplain could have provided a second significant source of the frequent quartzose pebbles in the Feltwell Formation glacial sediments of the East Fenland margin (cf. §3.3).

## 10. Chronology of Fenland events

Following the period of uncertainty regarding the timing and extent of glaciation during the late Middle Pleistocene in Britain (cf. §2), recent research has demonstrated that the Fenland was glaciated during the Wolstonian (= Saalian) Stage, intermediate between the Hoxnian and Ipswichian stages (= Holsteinian to Eemian: [6,8,9,141]; broadly equivalent to Marine Isotope Stages [MIS] 11b-6) (table 1).

The age of the Tottenhill glaciation advance to the Skertchly Line complex in Fenland during the Late Wolstonian Substage (i.e. Late Saalian Substage, approx. MIS 6) has been independently demonstrated based on litho- and morphostratigraphical relationships in the region, optically stimulated luminescence (OSL) ages from individual localities (*ca* 160 ka), especially from Warren Hill (Three Hills) and Lynford, Suffolk and Tottenhill, Norfolk and the presence of an interglacial palaeosol developed on the deposits' surface ([11–14,17–20,36,142], SM Pawley, personal communication; [143]) and elsewhere. Suggestions that the glaciation occurred in the Middle Wolstonian (*ca* MIS 8), based on OSL ages [144–149] described as ‘inaccurately determined’ [150], are here rejected in the light of the modern multiple age determinations mentioned above. These ages confirm the stratigraphical evidence demonstrating that there is no reliable basis for identifying glaciation intermediate between the Anglian and Late Wolstonian Substage (= MIS 6) Tottenhill advance events in eastern England [13–16,151].

This age correlation is further supported by correlation with The Netherlands and Germany, where the Late Saalian (Drenthe Stadial) glaciation is equated with MIS 6 (cf. [8,9,143,152–155]). Moreover, as Gibbard *et al*. [14,15] and Gibbard & Clark [20] discussed, this equivalence is further reinforced in the southern North Sea Basin. Here, offshore seismic evidence demonstrates the continuity of the ‘Skertchly Line’ glacial limit north of East Anglia with The Netherlands' Drenthe glaciation maximum [14–16,20,156,157]. The seismic analysis clearly differentiates this strongly defined feature from those of the earlier Anglian/Elsterian and later Devensian/Weichselian glaciation. The identification of the Tottenhill/Drenthe limit confirms that the glacial maximum identified in the Fenland region must therefore be of the same age, i.e. *ca* 175–155 ka, as Gibbard *et al*. [13] and Gibbard & Clark [20] concluded. Moreover, it demonstrates that the British and Scandinavian ice sheets were confluent at the time, as predicted by previous workers [124,158]. The implication of this confluence is that a substantial glacial lake would have formed in the southern North Sea Basin immediately south of the ice margin (cf. [152,155,159–163]) because drainage from both sides of the basin would have been prevented from flowing northwards.

Subsequent ice recession appears to have resulted in separation of the ice sheets, as indicated in geophysical plots [156,157], resulting in drainage of the North Sea glacial lake. As a consequence, this, potentially encouraged by the linked isostatic depression of the basin, allowed northward drainage of both Fenland rivers and meltwaters into the southern North Sea to be re-established.

As the Saalian/Wolstonian Stage represents an interval of *ca* 260 ka, i.e. twice the duration of the Weichselian Stage, division of the former into early, middle/mid- and late substages, based broadly on the equivalent MIS cycle divisions; MIS 11b–10, MIS 9–8 and MIS 7–6, respectively, has been proposed by Head & Gibbard [164]. Events within the substages are identified using the same climatostratigraphical approach as that of the Weichselian. Although the placement of the Tottenhill glaciation within MIS 6 confirms that it occurred within the Late Wolstonian Substage, the dates currently available imply that the glaciation predated the global ‘Penultimate Glacial Maximum’ or ‘PGM’ at 140 ka by as much as 20 ka (cf. [165,166]). That the glaciation was followed by a cold-climate interval of 25–30 ka, during which the Fenland river system continued to evolve, is indicated by the post-Tottenhill–pre-last interglacial fluvial sequences surrounding the basin. This interval is broadly time-equivalent to the Warthe Stadial (*ca* 155–126 ka) of the Late (Younger) Saalian in northern Europe (cf. [8,9,153,167]) and includes the period which saw the periglacial erosion of the Gault Clay bedrock to the west of the Travellers' Rest Gravels in Cambridge (see above).

The dating of the Tottenhill glaciation to the Late Wolstonian also indicates that the preceding period, the Early to Middle Wolstonian substages, lasted over 200 ka. While various cold-stage fluvial and related accumulations, as well as the High Lodge interglacial/interstadial event, have been identified within this interval, they generally occur in river valleys entering the basin or on the basin margins, rather than within the basin itself. This implies that erosion was the dominant process shaping the basin throughout the Wolstonian Stage.

Subsequent Ipswichian Stage (= Eemian Stage, *ca* MIS 5e) interglacial sequences, occurring at or close to the modern floodplain level in river valleys throughout the region, confirm that the present drainage system was in place by this time. Given the fact that it is known that Ipswichian sea level was somewhat higher than at present [168], this suggests that the deposits preserved may represent eroded remnants of a once thicker sedimentary fill. Later valley evolution, incision and accompanying fluvial sediment deposition are indicated throughout Fenland and beyond.

## 11. Summary: Middle to Late Pleistocene history of Fenland

**Figure 17.**
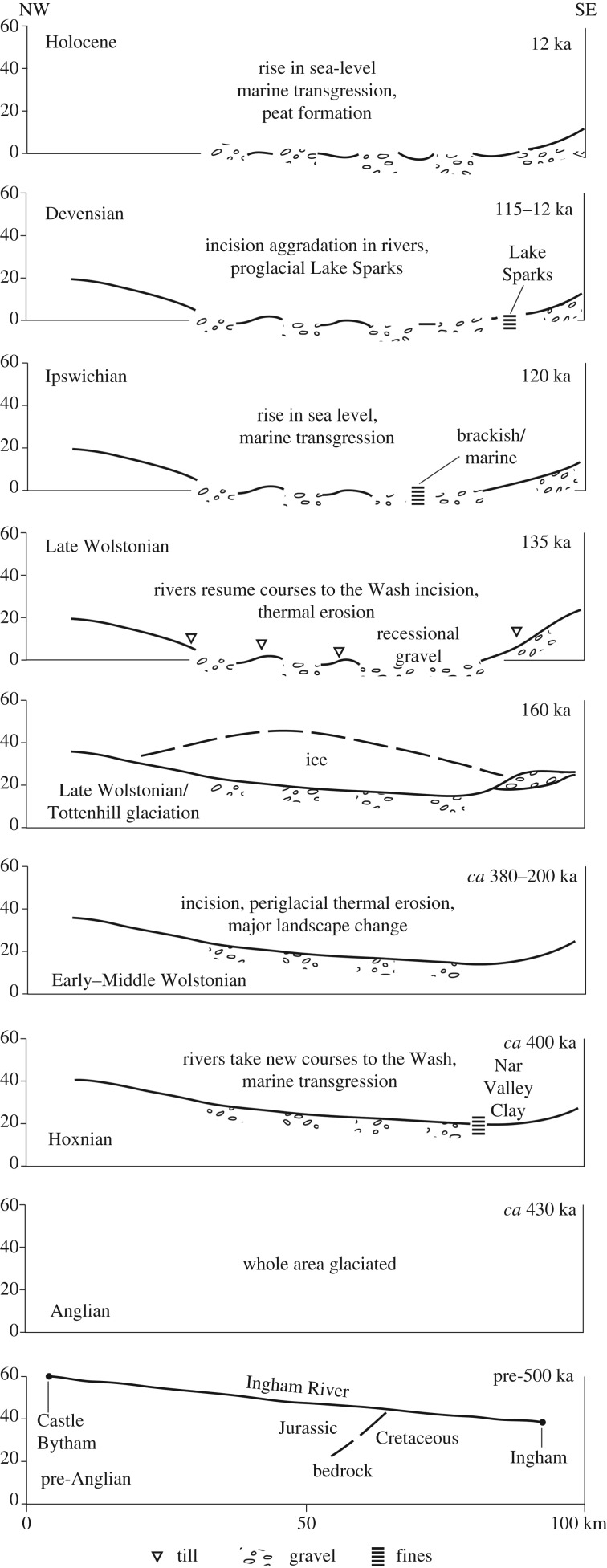
History of the Fenland Basin as seen in cross sections through the Middle to Late Pleistocene.

**Figure 18.**
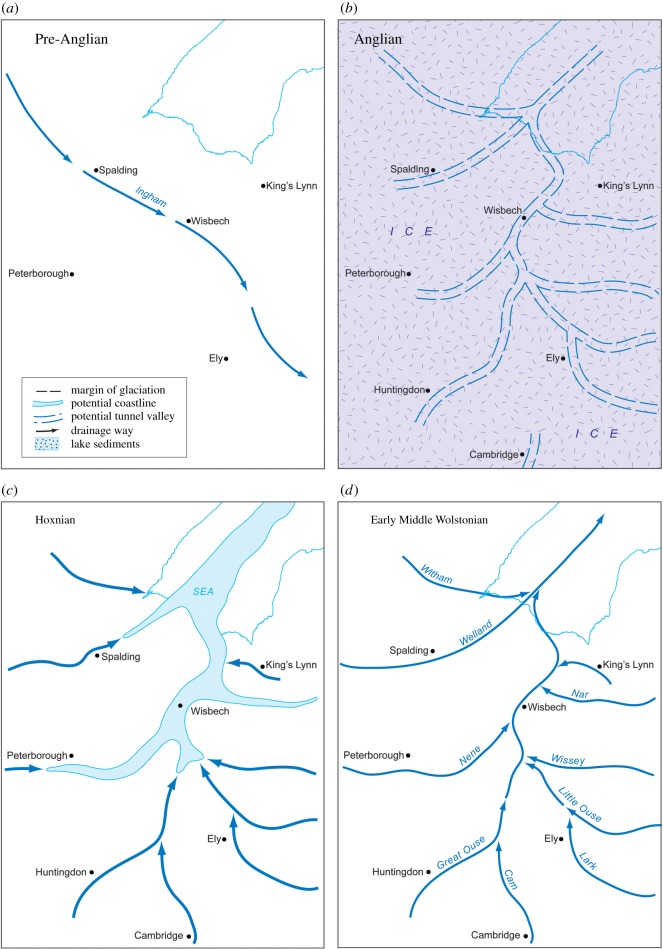

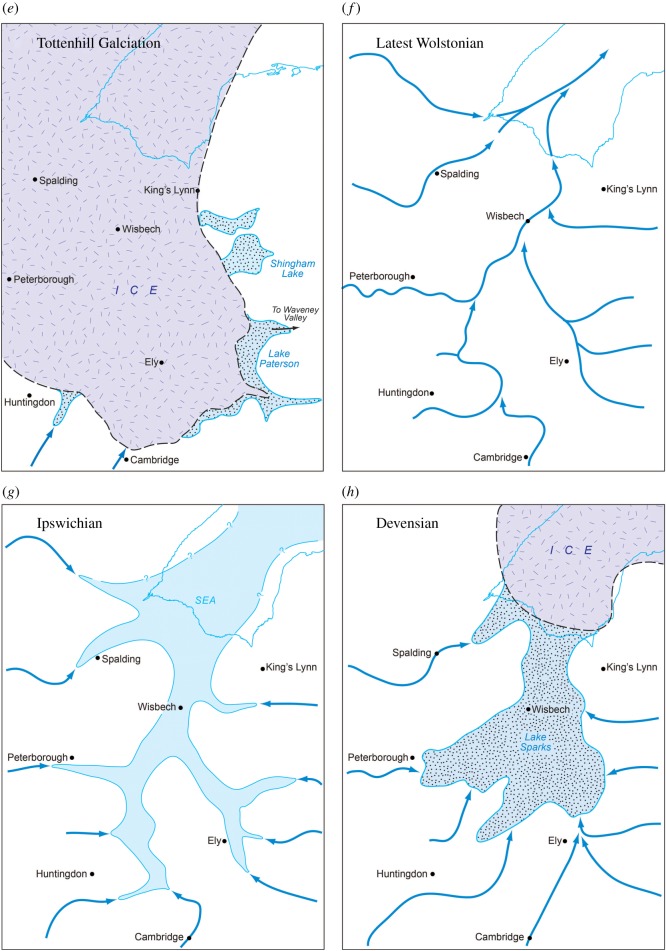
Middle to Late Pleistocene palaeogeography of Fenland.

This section presents a summary of the geological development of the Fenland based on the evidence assembled in this report (table 1). The evolution of the palaeogeography, stage by stage is shown in figures 17 and 18.

### 11.1. Pre-Anglian Pleistocene

Although no deposits are apparently preserved from the pre-Anglian Stage interval, it is possible to offer some broad observations about the general form of the Fenland area landscape. Before the East Anglian region was glaciated, the region was subjected to long-term evolution, predominantly under periglacial conditions. In common with much of lowland western Europe, the fluctuating climates of the later Pleistocene with periglacial conditions, frost-weathered bedrock and seasonally focused run-off led to episodes of river incision and deposition.

The recognition of the course of a palaeo-Trent river, aligned from WNW–ESE across the Fenland, based on the recognition of the East Midlands-derived Ingham Formation gravels in central Suffolk (previously related to a ‘Bytham’ stream: for discussion compare [27]), implies that the Fenland landscape in pre-Anglian times was comparable in form to the areas bordering the present Fenland. The youngest unit that can be traced across the region is the Kirby Cane Member in northern Suffolk, which underlies the Timworth Terrace surface of the Ingham stream, according to Rose *et al*. [169], Lewis [170] and Lee *et al*. [26]. The gradient of this unit, if projected upstream of the closest locality, Hengrave near Bury St Edmunds, where the unit occurs at 26 m O.D., implies the braidplain would have crossed the modern Fenland Basin at 35–45 m O.D. The implication is that the contemporaneous interfluve topography would have to have exceeded this figure, by possibly as much as 10 m or more. Interestingly, a reconstruction by Clayton [171], based on landscape modelling, reached a similar conclusion. Small streams may also have been aligned subparallel to the Ingham major drainage line, i.e. WNW–ESE (cf. [172]), while locally dry valleys would have been expected. It is reasonable therefore to assume that the Fenland landscape, in common with those across much of lowland western Europe, was subdued and gently undulating. In other words, no basin existed in the area before the Anglian Stage.

### 11.2. Anglian Stage

The arrival of glacial ice in the Anglian Stage caused a profound change in the Fenland region landscape evolution. The Anglian glaciation began when an ice lobe advanced across the North Sea Basin from the NE, overriding the pre-existing landscape throughout the north and east of East Anglia. This advance deposited a complex suite of interbedded tills and associated meltwater sediments, collectively termed the North Sea Drift Formation [20], but it did not override the Fenland. However, the arrival of ice of British origin advancing through Lincolnshire and the adjacent North Sea Basin into central and western East Anglia, crossed the Fenland. This pattern of ice movement is thought to result from confinement of the British ice sheet by the Scandinavian ice occupying eastern Norfolk and the adjacent North Sea area. This confinement might explain the substantial erosion of the basin during the advance. Once in the Fenland area, the ice radiated outwards in a fan-like pattern and continued to excavate the Mesozoic clay landscape (cf. [171]). Over a large part of East Anglia and the adjacent south Midlands, the British ice deposited the so-called Lowestoft Formation diamicton (till) and equivalents, and associated meltwater deposits.

Throughout East Anglia a series of deep, steep-sided valleys are cut through the Chalk and associated Mesozoic bedrock. These valleys form a radiating pattern, broadly parallel to the dip of the Chalk, that is, they trend west–east in the north and north–south in the south and north–east–south–west in the west of the region. Investigation of these ‘tunnel valleys’, by Woodland [173], Cox [174], Cox and Nickless [175] and van der Vegt *et al*. [176,177] among others, indicates that they are generally filled with glacial sediments, predominantly meltwater sands, gravels or fines, deposited by the Lowestoft Formation ice. Tills also occur but are less frequent. Closely comparable in form and scale to the *rinnen* of northern Europe, they undoubtedly result from subglacial drainage discharge under high hydrostatic pressure [176,178].

Around the Fenland a series of these tunnel valleys occur to the east, southeast and west, broadly parallel to the ice movement of the Lowestoft ice sheet. These valleys were potentially confluent at the northern end of the basin, radiating southwards in fan-like pattern, possibly in response to bedrock strike and/or movement of the British ice lobe. In particular, a substantial northeast-aligned tunnel valley occurs south of Spalding, Lincolnshire (BGS, Geological Map Spalding sheet, 144). This depression continues ENE beneath the Wash, where it is over 60 m deep and is partially filled by glacial deposits [136]. Beyond, it can be traced northeastwards into the substantial Silver Pit tunnel valley, off the coast of northern Lincolnshire [138,139]. In the central Fenland, one such infilled tunnel valley, over 27 m deep, occurs at March ([2]; S Boreham & PL Gibbard, personal observation), while marginal valleys include those underlying the Nar, Lark, Cam, Great Ouse and the Nene valleys. It is uncertain whether the tunnel valleys were confluent in the area south of the Wash, but the occurrence of the substantial Wash tunnel valley suggests that the breach between the Lincolnshire and Norfolk chalk originated from the incision of this subglacial landform. Glacial erosion may have further widened the gap once formed.

During deglaciation, meltwater rivers and glacial lakes became concentrated in the series of these large channel-like valleys, the alignment of which was determined by the subglacial ‘tunnel valley’ courses, especially where these valleys were only partially infilled by glacial sedimentation. In the Nar Valley [108,140] where the ‘tunnel valley’ deepens towards the west, that is, in the direction of the ice retreat, a proglacial ice-dammed lake formed in the valley, causing laminated clays to accumulate. Subsequent deglaciation gave rise to drainage of the lake and establishment of fluvial sedimentation at the transition to the subsequent Hoxnian interglacial Stage. Similar valley fills occur beneath the Great Ouse and the Nene valleys [91,179].

### 11.3. Hoxnian Stage

Following deglaciation during the latest Anglian time, local rivers established new courses on the deglaciated terrain. Initial freshwater sedimentation gave way to estuarine/marine deposition as a glacio-eustatic marine transgression entered the valleys. According to Ventris [140] the marine incursion originated from the area of the modern Wash and proceeded up the open tunnel valley(s) from west to east. The earliest marine incursion in the west is recorded at −2 m O.D. at Setch, south of Kings Lynn, during Hoxnian Substage Ho II, while the distribution of the marine/estuarine, Hoxnian interglacial-age Nar Valley Clays indicates that transgression reached a maximum proven altitude of approximately 23.5 m O.D. [140,180] in the east.

In southern Peterborough, on the western side of the Fenland Basin, the Woodston Beds [91,181,182] represent an equivalent accumulation to the Nar Valley Clay. These deposits record a marine transgression into the freshwater River Nene Valley potentially up to 6 m O.D. The abundant fauna of the Woodston Beds indicates deposition in a fluvial/estuarine situation. The palynology of these sediments confirms that they accumulated during Hoxnian Substage Ho IIc [91,178,179]. Correlation with the Hoxnian Stage is strengthened by coleopteran and numerical dating evidence [87,140].

The Hoxnian interglacial marine sequences in the Fenland valleys represent the lateral equivalent of the remarkably parallel Svarte Bank Formation infilling of the Silver Pit tunnel valley, on the North Sea floor [138,139], further emphasizing the connection of the Fenland Valley system with that beneath the adjacent North Sea.

The absence of Hoxnian-age freshwater and estuarine/marine deposits from the Fenland, south of a line from the Nar and Wissey valleys interfluve in the east to southern Peterborough, is significant. It implies that the land to the south must have exceeded the maximum marine transgression height, i.e. at least 25–26 m O.D. The occurrence of high ground, capped by gravel of the Ingham (palaeo-Trent) stream and possibly also Lowestoft Formation deposits, forming a WNW–W extension of the interfluve across what is now the Fenland Basin, has already been mentioned. To have acted as an effective barrier, such high ground would have had to extend across the entire Fenland Basin, forming the Hoxnian North Sea coast. The presence of such high ground implies that the southern part of the Fenland Basin did not exist during Hoxnian times.

### 11.4. Early to Middle Wolstonian substages

Climatic deterioration, following the Hoxnian interglacial, returned in the earliest Wolstonian. This cooling, accompanied by glacio-eustatic sea-level fall, and the return of periglacial climates, resulted in rivers extending and adopting courses focused on the Wash area. The lack of deposits preserved from this interval, probably a result of later glacial scouring, makes it difficult to present anything other than a cursory review of events in the Fenland at this time, although detail can be increased by including evidence from the surrounding areas.

What is certain is that during post-Hoxnian/pre-Tottenhill glaciation times a major period of evolution must have occurred in the Fenland Basin, markedly lowering the landscape associated with streams entering Fenland. This *ca* 200 ka period was dominated by cold climates, punctuated by occasional warmer climate events, one such being that represented at High Lodge, Mildenhall, Suffolk, where the High Lodge clayey silts and underlying sands infill one of a group of doline hollows in the Chalk bedrock. This forested cool-climate, temperate, interglacial or more probably interstadial event occurred within the Wolstonian Stage (possibly equated to MIS 7), while the solution hollow in which the deposits accumulated must have developed shortly before, following the melting of permafrost at the end of the preceding cold period, i.e. potentially the Middle Wolstonian Substage (possibly equivalent to MIS 8) [17]. Palaeolithic humans occupied the hollow during the warm period represented at High Lodge, where an *in situ* Palaeolithic flake industry has been recovered associated with lake sediments. Artefacts, including hand-axes, have also been frequently recovered from Feltwell Formation proglacial deposits in the district. Although the occurrence of the artefacts at High Lodge attests to the occupation of the doline and of immediate areas to the west of the Warren Hill ridge by Palaeolithic humans, the general occurrence and distribution of artefacts indicates that the humans were present throughout the Fenland district throughout much of the period from the end of the Hoxnian Stage interglacial to the Late Wolstonian glaciation.

Deposition during this period represents an exception; however, fluvial incision operating in concert with periglacial thermal erosion of the Mesozoic clays flooring the basin being the norm for the Early to Middle Wolstonian interval. An example is the River Lark at Mildenhall. Here, erosion of the Lark Valley (presumably under a cold climate), immediately adjacent to the Warren Hill ridge to the south and west, caused associated recession of the Chalk escarpment eastwards towards the ridge containing the High Lodge doline, causing local landscape levels in the area to be reduced by at least 25 m (cf. [17,32,183]). As noted above, this incision would have terminated the doline evolution as a consequence of the valley incision, locally exposing the relatively impermeable Lower and Middle Chalk.

Elsewhere in the areas marginal to the Fenland, river deposits representing this interval notably occur beyond the Tottenhill glaciation maximum limit. For example, in the Nene Valley the sequences underlying Terrace 3, and abutting the Woodston Beds date from this time, but are separated by an erosional interlude. The gravels underlying the River Great Ouse Terrace 2 surface at Stoke Goldington may also belong to this period [184].

While additional warm climate events have been claimed to be represented by fluvial deposits, for example in the Peterborough area, e.g. by Langford & Briant [89] and Langford *et al*. [93], the equivocal numerical dating of these units and their occurrence in part in an area apparently overridden by the Tottenhill ice lobe suggest that the dating of these sequences requires re-examination.

### 11.5. Late Wolstonian Substage (including the Tottenhill glaciation**)**

Climatic deterioration in the Late Wolstonian followed the temperate event represented at High Lodge (cf. above). During this time (the Drenthe Stadial) the Fenland region was glaciated by the Tottenhill advance (the deposits referred collectively to the Feltwell Formation). However, before the arrival of the ice lobe, periglacial conditions returned. Evidence for these conditions occurs at the Warren Hill Beech Clump locality where periglacial ‘rubble-chalk’ diamicton slope material, derived by freeze–thaw weathering of the underlying Chalk bedrock, is present. The cryoturbated upper surface of this local diamicton indicates subaerial exposure of the slope of the bedrock ridge [17].

During the Late Wolstonian, a substantial ice lobe advanced down the eastern side of Britain, entered the Fenland and continued to flow and fan outwards broadly towards the east, southeast, south and west. The advancing ice appears to have preferentially followed the Mesozoic clay outcrops towards the south, southwest and southeast, the ice apparently stalling against the rising ground underlain by the more resistant Jurassic limestones and associated rocks in the west and the Chalk in the east. The initial impact of the glacial ice-lobe advance towards its Skertchly Line maximum limit is poorly represented, although it is likely to have disrupted drainage, as well as eroded pre-existing bedrock and Quaternary materials alike. Evidence of this is found in the recycling of pre-existing materials in the glacial deposits. At the Beech Clump site the horizontally bedded sands rest directly on the rubble-chalk diamicton, indicating that they accumulated in a waterbody presumably dammed by the ice as it advanced towards the local hillslope. The combination of a ductile Mesozoic clay subglacial substrate and subglacial flooding would have made the ice lobe prone to surging, a situation important in the subsequent evolution of glacial sediments and landforms in Fenland.

Throughout the Fenland area the colour, texture and clast content of the glacial diamicton associated with this advance is predominently yellow-brown to red-brown in colour but it varies across the area, apparently being dependent upon the incorporation of local materials. The earliest glacial sediment recorded is yellow-brown silty clay-rich diamicton interpreted as basal-facies glacial diamicton (lodgement till) at the High Lodge site. Here it is overlain by glaciofluvial deposits. These sediments, together with the silts including the fossil assemblages and artefact industry, have been glaciotectonically dislocated and locally overridden by glacial ice post-depositionally. However, the general paucity of a regional diamicton (till) sheet from this glaciation may, in part, result from the surging behaviour of the ice lobe.

Following the advance of a glacial ice lobe into the Fenland Basin, the ice reached the marginal area in the southeast and was probably halted by the rising ground of the chalk hills to the east and south. From the evidence collected, it is likely that the ice advanced locally a few kilometres further towards the east and southeast up the valleys (e.g. the Lark) than the later ridges, to account for the finds of glacial diamicton (till) beneath the overlying sand and gravel accumulation, noted at some sites. This also accounts for the push structures noted above and for the deposition at the Rampart Field and equivalent localities.

Deposition of these ice-contact delta-fans (the Skertchly Line), which prograded into proglacial, ice-dammed lake or lakes in immediate contact with the ice lobe [14] arose from damming of the local streams, such as the Lark and Little Ouse. Initially, the lakes formed in each valley, but subsequently coalesed as the water level rose to form the extensive Lake Paterson in the south and southeastern Fenland marginal zone. Similar lakes formed in the Nar and Wissey valleys. Above the lake level, subaerial ice-contact fans accumulated, as at Shouldham Thorpe. The lateral distribution of the fans indicates a relatively regular spacing of meltwater conduits in the ice margin.

The glacio-marginal delta-fans consistently indicate a maximum lake-water level of approximately 30–32 m O.D. in Lake Paterson [14] although the level was falling throughout the period represented, interpreted as indicating progressive incision of the col as lake drainage occurred through the Little Ouse–Waveney valleys to the North Sea [50,53]. The meltwater from the Shouldham Thorpe fan presumably drained into the ice-marginal lake to the north in the Nar Valley at 12 m O.D. The position of the marginal fan accumulations in the landscape implies that the local topography at the time of their formation was already reduced to that seen at the present day, i.e. ranging from 17 to 32 m, i.e. substantially lower than the local Chalk hills to the east and the till plateau to the south, locally reaching over 50 m O.D. today.

Extension of the ice lobe further to the southwest and west has, until now, remained less certain, a consequence of later fluvial activity. In the Cam Valley, the 4th Terrace ridge at Stow-cum-Quy and Little Wilbraham and the Travellers' Rest Pit Gravels northwest of Cambridge appear to relate to marginal drainage of the glacier. However, tracing the ridge complexes described above, it is clear that the western lobe reached a maximum position possibly as far as Cottenham, continuing westwards towards Over, northwest to Bluntisham and Huntingdon, thence to the Folksworth interfluve, and to the Yaxley to north of Farcet ridge, reaching southeast of Peterborough. At Peterborough the complex internal structure of the ridge closely mirrors that seen in the eastern Fenland margin localities. It indicates both local ice-front oscillations and meltwater discharge producing marginal fans. Equally, here glaciolacustrine deposits, overlain by deltaic sands and gravels, indicate that the ice-dammed local streams (in this case the River Nene), causing a proglacial lake to form, which was later locally overridden. Finally, the overlying diamicton, rather than originating by subaquatic slide, almost certainly represents a glaciotectonic thrust. It is interesting to note that the substantial depression, occupied by the Whittlesey Mere and Ugg Mere basins, occurs within the semicircular, concave arc of this Yaxley to Wennington ridge complex (figure 3). From Peterborough, the ridge complexes extend northwards as far as the Grantham–Sleaford area of south Lincolnshire.

Diversion of the streams entering the Fenland Basin at the glacial maximum is repeatedly indicated. Langford [87] considered the Nene 3rd Terrace gravels, west of Peterborough, to represent in part glacial meltwater flow through the Southorpe Valley—linking the ice-dammed Welland and Nene valleys. This was initiated following diversion of the River Welland into the Nene Valley following glacial damming of the river east of Stamford.

Further evidence of glacial stream diversion, drainage of the local rivers, including the Cam and the Great Ouse, in common with the rivers of the Peterborough district would have been forced to flow marginal to the ice lobe(s) (cf. [15,16,87,135,136]). Discharge along or parallel to the ice front must have occurred, the water being forced to adopt a course from valley to valley and entering Lake Paterson in the east. This course is indicated by the accumulation at Hare Park where drainage was confined between the ice front and the bordering high ground. Latterly, the southward diversion of the Nene, east of Peterborough, and the Great Ouse, south and east of Huntingdon at Godmanchester, attest to the local redirection of the streams arising from the local impact of glacial-marginal bulldozing. This must have occurred after the marginal lakes drained when the river courses were being re-established. Local drainage was also controlled by the ice-pushed morainic ridges such that streams occupied the inter-ridge depressions as they became ice-free on deglaciation.

A consistent picture therefore emerges of a two-lobed ice advance, the margin of which was dynamic in its behaviour, the ice reaching a maximum position zone, yet oscillating over minor distances as it terminated within waterbodies dammed in the valleys entering the basin. The role of the water in potentially causing or amplifying these oscillations is unknown but changes of level during the ‘stillstand phase’ might be expected.

Having reached its maximum extent, the ice began to retreat but throughout the period maintained its dynamic activity, the ice front oscillating to generate the series of ice-pushed ridges that are now the Fenland islands and its margin and associated erosion hollows from which the diamicton material was quarried. These hollows may be the origin of the depressions which were to become the meres in the Holocene, all of which are in the marginal area of the glaciation. Once deglaciation began, the ice appears to have continued its oscillatory behaviour, the active ice-front stillstands or minor re-advances punctuating the general ice-lobe retreat. As many as four or possibly five such stillstands are indicated by these ridge complexes, which occur as bedrock/diamicton thrust structures on the Mesozoic clay bedrock, whereas on the Chalk, in the Mildenhall–Newmarket area, the stillstands are marked by ice-contact meltwater stream spreads. Here, possibly as many as four positions are indicated, which together with the push-moraines identified suggest a minimum total of six, or potentially seven or more stillstands.

As the ice lobe retreated, it is possible that it fragmented into two sublobes, separated by the north–south aligned ridge complex at March. A similar north–south orientated ridge occurs in the Littleport–Ely area and again might imply further fragmentation of the lobe structure.

During this period the Downham Market to Watlington gravel spread, along the northeastern Fenland margin, probably formed as a kame-terrace-like deposit laid down by northwards flowing meltwater marginal to the Tottenhill ice lobe. The altitudinal relationship of this unit to the Tottenhill delta demonstrates that the former spread postdates the latter. This interpretation implies that the kame-terrace almost certainly accumulated during a stillstand phase in the local ice recession and that by this time drainage was aligned northwards towards the North Sea via the Wash gap. This contrasts with the eastward drainage that occurred during the phase represented by the glacial lacustrine deposits at Tottenhill itself.

Assuming those rivers continued to flow throughout the period the Tottenhill ice lobe occupied the basin, deglaciation would have been expected to see the rivers reorganizing their courses on the newly deglaciated basin terrain. However, once the ice had retreated sufficiently to the north, the rivers would be expected to have migrated towards the basin centre. Such a process might explain the substantial distribution of NE-trending gravel and sands to the north.

In the southeastern Fenland, the substantial meltwater deposits that occur as discrete, laterally separated accumulations appear to have been laid down during progressive ice retreat that began in the east with the ice front retreating westwards in a punctuated series of stillstands. The distribution of these spreads, generally unrelated to the modern rivers yet cutting through the Fenland island ridges, suggests that they accumulated during the deglaciation, and that they are not ‘normal’ fluvial accumulations. That they originate from ice-contact, southward-flowing meltwater, their gradients aligned broadly towards the NE implies that they were deposited at tunnel portals. The fan-like form of the spreads suggests that they represent subaerial ice-front alluvial fans locally confluent fluvial and ice-marginal drainage alignment towards the Fenland Basin centre. This distribution is confirmed by the relationship of the spreads to the underlying bedrock topography, the gravels being aligned towards the central Fenland Valley.

Following deglaciation, the rivers re-established the drainage system. Downcutting caused the rivers to lower their valleys, cutting through the pre-existing glacial sediments, and in places, scouring them away completely. On the basis of the bedrock floor map, it appears that the rivers adopted a northward course towards the Wash and the southern North Sea Basin; an alignment possibly encouraged by glacial erosion and glacio-isostatic depression of the region. The rivers were apparently confluent north of the March ‘island’, the Cam system passing northwestwards from Cambridge. The Great Ouse passed to the east and southeast of Chatteris where it was linked to the River Nene. Realignment of the river Witham in the northwest of the basin may also have occurred during this interval.

In the Nar Valley this period is represented by emplacement of the cold-climate Wormegay Gravels. As mentioned above, this sequence is important because it demonstrates that downcutting and deposition under cold-climate conditions persisted after the glacial retreat. Moreover, this was followed by a second phase of downcutting, and deposition occurred, during which time the immediately pre-Ipswichian-age Marham Gravel was laid down. Gravel and sand units occur in comparable positions to the Marham unit throughout Fenland at sites including Block Fen, Somersham, Mepal, Maxey, etc.

Likewise in the Nene and Welland, the 2nd Terrace gravels and potentially the basal segments of the 1st Terrace accumulations almost certainly represent the same depositional system. Overall, therefore, these coarse arenaceous deposits represent cold-climate accumulations, predating the Ipswichian Stage, i.e. the deglaciation was not immediately followed by interglacial climatic amelioration. Their distribution demonstrates that the interval between the Tottenhill glaciation and the beginning of the last interglacial saw the Fenland Basin achieving its modern form. The deposition of cold-climate gravelly sediments flooring the river valleys, and immediately predating the Ipswichian interglacial accumulations, is a pattern repeated throughout lowland Britain and indeed in neighbouring countries, e.g. The Netherlands [185,186].

### 11.6. Ipswichian Stage

In the period of climatic amelioration marking the end of the Wolstonian, the beginning of the Ipswichian saw rising sea levels and deposition of sediments in the river valleys. These are recorded in a large number of sites in the Fenland, and combine to give much detail of the afforestation of the Fenland in the interglacial, with the pollen zones characteristic of the Ipswichian, particularly the succession from mixed oak forest (Ip II) to forest with *Carpinus* (Ip III). The interglacial sediments vary from sands and fines to highly organic muds, with Jurassic or Cretaceous clays or Late Wolstonian gravels below and Devensian gravels above. A fluvial sequence within a terrace of the Great Ouse Valley shows a sequence of alluviation during the interglacial [74].

A further significant point of interest in the vegetation succession of the period is a change towards the end of the *Carpinus* zone, when there is an increasing diversity of the non-tree pollen, with higher frequencies of pollen of, *inter alia*, Gramineae, Compositae and *Plantago* species. This change is seen in the pollen diagram from the site at Histon Road, Cambridge, underlying the Terrace 3 surface of the Cam Valley [65] and at Chatteris [82], indicating a vast change in the landscape of the area, with the vegetation becoming more open and varied. The change is likely to be related both to climatic change and the taphonomy of the pollen recorded in the sediments.

The response of lowland English rivers to interglacial climatic conditions has been considered by Gibbard & Lewin [185]. They demonstrated that during temperate events, the establishment of little or no erosion, the development of cohesive channel banks and vertical accretion as the controlling sedimentation process result in the dominance of fine and organic sedimentation as channel fills or flood deposits. Reduction in the number of channels occupied by flowing water to one or two by progressive sedimentary infill encouraged the increased frequency of overbank flooding and thus floodplain sedimentation. This typical stable meandering or anastomosing river behaviour results in floodplain areas beyond flow channels accumulating marsh or fine-grained sediment. Such areas locally supported a dense woodland complex, with moist as well as drier areas, being colonized by diverse communities of plants and animals. Increasing opening of the vegetation through the latter half of the Ipswichian (Ip III–IV) led to a predominance of inorganic floodplain sedimentation by localized inwash of soil and later regolith materials.

The interglacial sediments include both freshwater and brackish/marine deposits. It is possible to demonstrate the presence of a marine transgression into Fenland during the Ipswichian, and its approximate timing. Brackish/marine sediments, identified by analyses of the molluscs, occur at March [104], Somersham [112], Block Fen [99] and Wretton [187], and show that the effects of the transgression stretched south up Fenland rivers to the Fenland margins at Somersham and to the east at Wretton.

The heights O.D. of these brackish/marine sediments at particular sites are shown in figure 12, along the section from Chatteris to the Mepal depression. Also shown are the heights at March Town End and Wretton and the approximate height limits of the freshwater and brackish/marine sediments in various sections at Somersham. The Somersham and Block Fen sites are associated with the former course of the River Great Ouse, and show a variation from −6.0 m to 2.0 m O.D, the height dependent on the position of the sediments within the drainage system. Transgression appears to have taken place in pollen zone Ip II, and regression in Ip III, though recorded in river valleys, may have been much more extensive, and possibly filling the entire basin.

### 11.7. Devensian Stage

The Devensian Stage is represented in the Fenland by gravels forming terraces in the lower reaches of the rivers which drain to the Wash. They extend below the Holocene sediments of the Fenland, and are separated from them by a thin horizon of clay with scattered pebbles in the Peterborough area, the Crowland Bed of Horton [85] and to the east in the Wisbech/King's Lynn area [106]. This diamicton appears to be a head related to a time of low sea levels in the Devensian, postdating the underlying terrace formations.

Devensian terraces are present in the valleys of the Welland, Nene, Great Ouse, Cam and Nar rivers, as described above. They are less well developed in the Wissey, Little Ouse and Lark valleys, potentially a consequence of their relation to the local bedrock Chalk aquifers. There is a wealth of information within or related to the gravel sequences, ranging from the rich palaeobotanical contents of fine and organic sediments within the gravels, as at Somersham [112], to indicators of permafrost conditions within or after the aggradations. There is also the wider influence of periglacial conditions in the wider landscape shown by depressions formed by thermal erosion [188]. The Devensian 4 m terrace of the Wissey at Wretton contains evidence for a complex of terrace sands interdigitating with more organic sediments, including events of interstadial character in an earlier part of the Devensian [109].

The study of the Devensian aggradations has led to two areas where taphonomy has complicated environmental interpretations. The first is the occurrence of gravelly beds containing fossils possibly reworked, most often occurring in the basal parts of aggradations, where the local landscapes have been swept by the onset of aggradation (cf. the situation described by [189] in relation to palaeoliths near the base of gravels). This situation may be revealed by the diversity of the palaeontology recorded, as at Eye (see discussions in [92] and [184]). The second follows from the presence of marine shells in certain of the gravels, including the March Gravels [190,191]. These have often been interpreted as marine sediments. There is evidence for brackish/marine conditions during the Ipswichian interglacial as far south in the Fenland as Somersham [112] and Block Fen [82]. At Somersham, Devensian gravels overlying Ipswichian brackish sediments contain a diverse reworked assemblage [98]. Reworking of Ipswichian brackish/marine sediments in the Devensian has evidently led to the incorporation of fossils, indicating brackish/marine conditions into the gravels, rather than the gravels being of marine origin. Thus the extent of the gravels with marine shells gives an indication of the extent of the marine transgression in the Ipswichian.

A further important development in the Devensian was the wide dispersal of cover sands, largely originating from the sand beds of the Wolstonian glacial lake deposits over the landscape, giving the character of the Breckland of East Anglia.

During the Late Devensian the Wash was invaded by the ice which deposited the Hunstanton Till, at *ca* 20 ka. The margin of the till is shown in figure 18*h*.

As a result, a lake was impounded in the Fenland, named Lake Sparks [114]. Laminated sediments indicating the presence of a lake were found at Somersham, on the southern margin of the Fenland. Here, varves were deposited over a *ca* 65-year period. Lacustrine sediments probably related to this lake are present within the Block Fen gravel sequence [82]. These sediments appear to be the only evidence for such a lake, indicating a wide degradation of the landscape in later times when Fenland drainage resumed after the retreat of the ice.

There are a considerable number of radiocarbon dates from the Fenland Devensian organic sediments or bones within the gravel aggradations, including the sites at Barnwell Station [68], Block Fen [82], Earith [192], Sidgwick Avenue (Cambridge) [64], Somersham [112] and Wretton [109]. The dates range from 42 to 18.5 ka, related to gravel aggradations in the later part of the Devensian, the latest also relating to the formation of Lake Sparks and the Devensian glaciation (*ca* 18.5 ka uncalibrated).

## 12. Conclusion

This summary has demonstrated that the formation of the Fenland Basin was multiple-phased, resulting from the complex interaction of cold and temperate climate processes over the last 500 ka, where easily eroded bedrock provided a preferential pathway for glacial advance during two glaciations.

On the basis of the results presented here, it appears that the basin began forming during the Anglian glaciation as a consequence of a subglacial meltwater tunnel valley that breached the Chalk bedrock in the area of the present Wash. A network of tunnel valleys was apparently confluent at the northern end of the basin and radiated southwards in a fan-like pattern. This pattern was possibly dictated by response to bedrock strike and/or the movement of the Anglian ice lobe. This glaciation was marked by southwards advancing ice that overrode the entire region and in the process destroyed the pre-existing NW to SE drainage system.

Following deglaciation, a new drainage system began to develop on the hummocky glacial terrain, the partially infilled tunnel valleys acting as conduits guiding the initial drainage pathways. During deglaciation glacial lakes formed in the tunnel valleys, such as the Nar Valley, resulting from the glacially impeded northward-aligned drainage. Once the blockage was removed, normal rivers occupied the valleys but they were quickly overwhelmed by marine transgression into pre-existing valleys, of the Nene at Peterborough and the Nar, near Kings Lynn, the sea reaching a maximum height of approximately 24 m O.D. As the marine transgression did not enter the March and Cam tunnel valley systems, it is assumed that they were completely infilled during the preceding glaciation. South of the line linking the Nar and Wissey valleys interfluve in the east, west to southern Peterborough, no marine deposits are found, this implying that the area was land that reached over 25–26 m O.D., forming the Hoxnian interglacial North Sea coast.

Following the glacio-eustatic sea-level regression at the end of Hoxnian, the rivers followed the retreating sea to establish courses across the Fenland, superimposed on marine/glacial surface. Potentially the tunnel valleys could have provided a broad control of course alignments, but this cannot be demonstrated in the main basin area today because of the substantial subsequent erosion. The Early to Middle Wolstonian in lowland Britain was characterized by predominantly cold climates, which saw a long period of periglaciation that made a major impact on the landscape. During this period of almost 200 ka the river systems evolved and matured, valleys including terrace accumulations of coarse fluvial sediments. The rivers generally incised into the pre-existing materials, the predominantly low sea levels allowing the rivers to extend beyond the present Wash into the North Sea Basin beyond.

Although the period was dominated by cold climates, warmer climate interludes also occurred. Of particular significance is the High Lodge event, represented by the sediments which occur within one of a suite of doline solution hollows in the Chalk bedrock, the doline having formed at the end of the previous Middle Wolstonian cold interval (i.e. MIS 8). This Late Wolstonian forested cool-climate, interglacial or interstadial event (potentially equivalent to MIS 7) included the presence of Palaeolithic humans. Humans had been present throughout much of the preceding period from the Hoxnian interglacial, their artefacts being found repeatedly in many of the deposits from the period and reworked subsequently into younger accumulations.

The deterioration of the climate during the Late Wolstonian saw a return to periglacial climate with ‘rubble-chalk’ diamicton slope deposits resulting from freeze–thaw processes on exposed surfaces. The arrival of a glacier from NW–N resulted in a substantial ice lobe invading the basin, fanning out towards the margin. The advancing ice appears to have preferentially followed the Mesozoic clay outcrops towards the S, as well as towards the SW and SE, the ice jamming on the rising ground underlain by the more resistant Jurassic limestones and associated rocks in the west and the Chalk in the east. Initial blockage of the drainage system entering Fenland must have occurred but no evidence remains from this time apart from the lacustrine sands overlying the rubble-chalk diamicton at Warren Hill, Suffolk. During the advance the ice excavated materials to expand the lobate form of the basin, the material being largely removed by meltwater recycling. Diamicton from the glaciation is remarkably rare, but pre-existing diamicton inherited from the earlier Anglian glaciation was bulldozed and remobilized by the Tottenhill advance. Glaciotectonism resulted in a series of low, subparallel ridges being pushed up around the margins of the lobe on the western, southern and southeastern margins, whereas in the east the Skerchley Line limit is marked by the deposition of delta-fan and alluvial fan complexes, the former deposited into marginal lakes resulting from damming of the streams entering Fenland from the surrounding unglaciated terrain. This ponding of the rivers is seen not only in the east but also in the Great Ouse Valley west of Huntingdon, in the Nene Valley near Peterborough and in the Welland Valley. Drainage during this phase was potentially either over, under or around the margins of the ice lobe, until the lake in the Lark Valley was encountered. The latter, called Lake Paterson, progressively filled with water until the lake overspilled into the Waveney system, the water then draining into the southern North Sea. Once overspill had occurred, the breach between the Lark and Waveney valleys became progressively lowered, leaving lake sediments on the valley sides above the rivers.

The Fenland ice lobe associated with the Tottenhill advance was similar in form and distribution to ice lobes that occurred at the southern limits of the last Laurentide Ice Sheet. The low gradient of the Fenland ice lobe was facilitated by the underlying easily deformable Mesozoic clays at the glacier bed combined with impeded subglacial drainage associated with the ice-marginal lakes as the ice advanced into the Fenland. As with the ice lobes associated with the last Laurentide and the Valdaian Kara Sea ice sheets, the Fenland lobe would have been prone to surging behaviour.

Ice-front oscillation, probably arising from periodic surging, produced not only glaciotectonic push-moraines, but also equivalent erosional depressions on their up-glacier sides (e.g. the meres), which today are occupied by Holocene Fenland deposits. As the ice retreated, the ice front remained active so that suites of subparallel pushed ridges mark the basin margins. These ridges comprise predominantly diamicton materials but on the Chalk bedrock the stillstands comprise meltwater sands and gravels, indicating glaciofluvial discharge preferentially from a series of portals in the ice margin. Drainage towards the east was redirected northwards later to form kame-terrace deposits on the northern basin margin in the Downham Market–Nar Valley area, indicating that later in the deglaciation phase drainage ceased to flow southeastwards but was realigned northwards towards the North Sea. This change may have corresponded in part to fragmentation of the glacial lobe into two or three sublobes. Continued ice retreat saw the rivers re-establish their courses towards the centre of the Fenland Basin and exit via what is today the Wash embayment, possibly encouraged by isostatic downwarping of the basin and adjacent North Sea floor.

Despite unsubstantiated suggestions of older ages, the numerical dating available establishes beyond doubt that the Wolstonian Tottenhill ice-lobe glaciation occurred during the Drenthe Stadial of the Late Wolstonian (i.e. Late Saalian Substage; MIS 6; *ca* 160 ka). The advance is therefore broadly coeval with similar maximum ice-lobe advances in The Netherlands, western Germany and the intervening southern North Sea Basin.

As the ice retreat progressed and rivers reoccupied the basin, periglacial processes returned to modify further the freshly deglaciated landscape. Incision in the rivers was followed by accumulation of new post-glaciation terrace gravels and sands, which were deposited during the post-Drenthe, Warthe Stadial phase of the Late Wolstonian (Late Saalian; late MIS 6). Further downcutting saw the emplacement of low-level gravel and sand units in all the major tributary valleys and indeed their extensions in the Fenland itself.

The valley floor gravels in many valleys are overlain by fine-grained Ipswichian-age interglacial sediments at sites including Block Fen, Somersham, Pentney, etc. The rivers continued to the southern North Sea via the Wash. This initial freshwater alluviation ceased when marine transgression during Substage Ip II resulting from rising glacio-eustatic sea level resulted in drowning of the river valleys. This transgression is represented by intertidal sediments at Somersham. Temperate forests colonized the valleys and basin interfluve surfaces. Initially this was dominated by mixed oak tree taxa but in the second half of the period increasingly diversity of the non-tree taxa is accompanied by the establishment of *Carpinus* (hornbeam) in the woodland assemblage. Overbank flooding of the rivers resulted from the progressive infill of channels with inorganic alluviation. This resulted from increased fluvial discharge. Consequently, fluvial sedimentation replaced estuarine conditions once sea levels began to retreat during the latter half of the event, beginning in Substage Ip III. No evidence of human occupation is known from Fenland during this temperate period. Claims that the March Gravels represent littoral deposits laid down during this Ipswichian high sea-level event have been shown to be false, the occurrence of marine fossils in the gravels being a consequence of later reworking of interglacial marine sediments by fluvial processes.

The Devensian Stage in Fenland is marked by a return once again to predominant cold climates and periglacial weathering of exposed surfaces. The main river valleys all preserve remnants of terrace accumulations, especially in the Welland, Nene, Great Ouse, Cam and Nar valleys. A wealth of detail is available on the local environments and colonizing biota in these valleys, in particular at Wretton, Norfolk where there is evidence of interstadial conditions. As stated above, the occurrence of reworked marine fossils in the Devensian gravel units is common, e.g. at Somersham and the March area.

Throughout the Devensian Stage and in particular following the Middle Devensian during which wide spreads of fluvial gravels and sands were laid down, a series of fluvial realignments occurred, possibly reflecting local capture and degradation of low-level confining bedrock interfluves. The migration of the river Cam from its initial northerly to a progressively more easterly alignment is a good example, but the Great Ouse appears to have undergone a similar course readjustment towards the W or NW. The Devensian also saw a wide dispersal of aeolian cover sand over the landscape east of the Fenland Basin.

During the Late Devensian Substage the Wash was invaded at *ca* 20 ka by glacial ice of the North Sea ice lobe. The arrival of the ice blocked the Fenland drainage once more and a proglacial Lake Sparks was formed in Fenland at *ca* 18.5 a C14 BP, with an estimated water level of approximately 5–10 m. Although this lake probably only existed for a few decades, subsequent renewed incision by the rivers followed drainage of the lake once the ice lobe disintegrated under continuing cold, periglacial conditions of the late-glacial. Holocene Fenland deposits then accumulated on the buried valley and interfluves of the basin alike.

Finally, three general conclusions can be made. The first, that the Fenland Basin results from breakthrough of the Chalk at the site of the Wash, and the excavation along its strike of the Mesozoic clay bedrock. Secondly, that the periglacial thermal erosion of the Mesozoic claylands is central to the evolution of the basin landscape occurring throughout both the Wolstonian and the Devensian cold periods (over 300 ka). Thirdly, it is interesting to note that the human occupation of Fenland during the Holocene is paralleled by its occupation in Palaeolithic times.

## Data accessibility

Data are available from the references cited and the public information cited in the text. The latter from publications and website borehole archive of the British Geological Survey: www.bgs.ac.uk.

## Appendix A. Lithostratigraphical classification of the Fenland glacial deposits: the Feltwell Formation

Following the detailed investigations of the eastern Fenland margin sites, discussed in this article, the need to define formal divisions of the newly established glacial sequence was recognized. For purely practical purposes, such divisions are lithostratigraphical because they are dependent upon the lithology, sedimentary characteristics, structure, relationships, etc. of the individual units that represent the sequence as a whole. These divisions provide the physical basis for mapping and associated investigations at a range of scales from specific sites to regional compilation and correlation.

In order that the division is practical to apply and facilitates efficient communication, stratigraphical subdivision is hierarchical. Moreover, practicality in regional classification demands systematic lithostratigraphical subdivision [12,13,193–195]. Therefore, when new or modified divisions are proposed, they are defined at the appropriate scale and are consistently applied to ensure efficient communication, and, where appropriate, take note of historical precedence.

With these concepts in mind, Gibbard *et al*. ([15,16]) proposed a new unit to include ‘all the glaciofluvial deposits of the Fenland margin, but also the associated diamicton and other related sediments in the area arising from the same glacial event and therefore possessing the same basic lithological elements’. This unit, termed the Feltwell Formation, is analgous in concept to the previously established glacial, Lowestoft or North Sea Drift Formations of eastern England.

The Feltwell site in Norfolk was selected as the stratotype (NGR TL 740922) because the sequence here comprised a complex of coarse to fine stratified sediments associated with diamicton units, mass-flow deposits and solutional collapse features resting on Chalk bedrock, described in detail by Gibbard *et al*. [36]. This sequence was deemed representative of the glacial sequence of the eastern Fenland margin as a whole.

In view of the greater extent of the glacial materials associated with the Fenland glaciation established in this article, it is proposed to extend the term Feltwell Formation to include the total suite of glacial deposits, including the complex sequence of stratified and sorted gravel, sand and fine sediments, intimately associated with diamicton and locally disturbed by localized collapse and glaciotectonic structures arising from the Fenland (Tottenhill) lobe. To date, individual units included within the Feltwell Formation have not been assigned formal names, with the exception of Shouldham Thorpe Member, but it is intended that when this is necessary, they are likewise considered as member- or bed-status units, as appropriate (cf. [36]).

## Appendix B. Palaeolithic archaeology

Wymer [30] listed a large number of scattered sites where palaeoliths had been found in the Fenland, including those recorded by Baden-Powell [113,196]. Many are associated with the gravel pits of the ‘March Gravels’ and their exact provenance is uncertain. Few have been found *in situ*, but one is reported to have been found in ‘Gipping Boulder Clay’ at Ancaster Farm, Stonea, identified as a tortoise core, and of unpatinated black flint [196]. The association of the finds with gravels indicates that these are reworked in the course of river development.

On the margins of Fenland, however, the situation is very different. Many sites associated with the Skertchly Line of Feltwell Formation proglacial gravels of Late Wolstonian age [14] have yielded abundant palaeoliths, the result of their dispersal by glacial action from the Fenland Basin. These sites, on the southeast margin of the Fenland Basin, include Gravelhill (Brandon), Maids Cross (Lakenheath), High Lodge (Mildenhall) and Three Hills (Warren Hill, Mildenhall). On the southern margin there are sites associated with proglacial gravels at Kentford and Hare Park, and to the southwest there is the Travellers' Rest Pit, with gravels associated with ice-marginal conditions. All these sites are considered in detail by Wymer [30].

These are all sites where the palaeoliths are reworked. But there is one site, High Lodge (Mildenhall) on the Fenland margin where *in situ* artefacts have been recorded, associated with deposition of fine sediments in a doline in the Chalk, predating the deposition of the Late Wolstonian proglacial gravels [197]. This site gives clear evidence of Palaeolithic presence in the neighbourhood in preglacial Wolstonian times, a presence which is likely to represent occupation in the wider area of Fenland, but where signs of occupation have been swept away, to be deposited in the proglacial gravels at the margins of Fenland. The predominant occurrence of these artefact assemblages in the chalk-rich gravels and sands indicates that they were reworked, together with other clastic material, including vertebrate skeletal fragments, from local sources by glacial action and redeposited within the delta-fan sediments.

The frequent occurrence of reworked palaeoliths in proglacial gravels related to the Tottenhill glaciation prompts a further discussion of the relation of the archaeology to the glaciation. Such gravels occur at the eastern and southern margin of the glaciation, to the east along the Skertchly Line, to the south at Kentford and Hare Park, and to the southwest at the Travellers' Rest site at Girton, Cambridge. Such gravels are not recorded on the margin of the glaciation to the west and north of the last-named site, where tills, reaching a substantial thickness, characterize the margin of the glaciation. It is clear that as a result of the glaciation, till deposition predominated in the west, in the area of Jurassic clay bedrock, while to the east, in the area of Chalk bedrock, a different pattern of till deposition took place, exposed on deglaciation together with extensive sheets of meltwater gravel. The association of reworked palaeoliths in the eastern area must result from erosion of Early Wolstonian surfaces and river gravels by the glaciation in the eastern part of Fenland with redeposition and concentration of the artefacts in the marginal gravels, a process not seen in the western part. These comments illustrate the complex nature of the events during the Tottenhill glaciation.

## References

[1] Godwin H 1978 Fenland: its ancient past and uncertain future. Cambridge, UK: Cambridge University Press.

[2] Gallois RW 1988 Geology of the country around Ely. Memoir Sheet 173. London, UK: British Geological Survey, Her Majesty's Stationery Office.

[3] Waller M 1994 The Fenland project, 9: Flandrian environmental change in Fenland. East Anglian Archaeology and Cambridgeshire County Council.

[4] Wheeler AJ 1994 Stratigraphy and palaeoenvironments of Late Holocene sediments in north-central Fenland, UK. PhD thesis, University of Cambridge, Cambridge, UK.

[5] Skertchly SBJ 1877 The geology of the Fenland. Geological Survey of England and Wales London, UK: Her Majesty's Stationery Office.

[6] Mitchell GF, Penny LF, Shotton FW, West RG 1973 A correlation of the Quaternary deposits of the British Isles In Geological Society of London, Special Report 4, 99p.

[7] West R 2016 *T.T. Patersons's contribution to Breckland Pleistocene geology in the 1930s; a tribute and commentary*. issuu.com/suffoknaturalistssociety/docs/paterson

[8] Litt T, Behre K-E, Meyer K-D, Stephan H-J, Wansa S 2007 Stratigrafische Begriffe für das Quartär des norddeutschen Vereisungsgebietes. Eiszeitalter und Gegenwart 56, 7–65.

[9] Litt T, Schminke H-U, Frechen M, Schlüchter Ch 2008 Quaternary. In The geology of central Europe, 2: Mesozoic and Cenozoic (ed. McCann T), pp. 1287–1340, Ch. 20. London, UK: Geological Society.

[10] Lee JR, Rose J, Hamblin RJ, Moorlock BSP, Riding JB, Phillips E, Barendregt RW, Candy I 2011 The glacial history of the British Isles during the Early and Middle Pleistocene: implications for the long-term development of the British ice sheet. In Quaternary glaciations extent and chronology, a closer look, pp. 59–74. Developments in Quaternary Science Amsterdam, The Netherlands: Elsevier.

[11] Clark CE, Gibbard PL, Rose J 2004 Glacial limits in the British Isles. In Quaternary glaciations extent and chronology, part I: Europe, vol. 2a (eds Ehlers J, Gibbard PL), pp. 47–82. Developments in Quaternary Science, Amsterdam, The Netherlands: Elsevier.

[12] Gibbard PL, West RG, Andrew R, Pettit M 1991 Tottenhill. In Central East Anglia & the Fen basin field guide (eds Lewis SG, Whiteman CA, Bridgland DR), pp. 131–144. Cambridge, UK: Quaternary Research Association.

[13] Gibbard PL, West RG, Andrew R, Pettit M 1992 The margin of a Middle Pleistocene ice advance at Tottenhill, Norfolk, England. Geol. Mag. 129, 59–76. (doi:10.1017/S0016756800008128)

[14] Gibbard PL, Pasanen AH, West RG, Lunkka JP, Boreham S, Cohen KM, Rolfe C 2009 Late Middle Pleistocene glaciation in East Anglia, England. Boreas 38, 504–528. (doi:10.1111/j.1502-3885.2009.00087.x)

[15] Gibbard PL, West RG, Boreham S, Rolfe C 2012 Late Middle Pleistocene ice-marginal sedimentation in East Anglia, England. Boreas 41, 319–336. (doi:10.1111/j.1502-3885.2011.00236.x)

[16] Gibbard PL, West RG, Boreham S, Rolfe C 2012 Late Middle Pleistocene glaciofluvial sedimentation in Norfolk, England. Neth. J. Geosci. 91, 63–78. (doi:10.1017/S0016774600001505)

[17] West RG, Gibbard PL, Boreham S, Rolfe C 2014 Geology and geomorphology of the Palaeolithic site at High Lodge, Mildenhall, Suffolk, England. Proc. Yorkshire Geol. Soc. 60, 99–121. (doi:10.1144/pygs2014-347)

[18] Gibbard PL 1991 The Wolstonian Stage in East Anglia. In Central East Anglia & the Fen basin field guide (eds Lewis SG, Whiteman CA, Bridgland DR), pp. 7–13. Cambridge, UK: Quaternary Research Association.

[19] Lewis SG, Rose J 1991 Tottenhill. In Central East Anglia & the Fen basin field guide (eds Lewis SG, Whiteman CA, Bridgland DR), pp. 145–148. Cambridge, UK: Quaternary Research Association.

[20] Gibbard PL, Clark CD 2011 Pleistocene glaciation limits in Great Britain. In Quaternary glaciation extent and chronology: a closer look (eds Ehlers J, Gibbard PL, Hughes PD), pp. 75–94. Amsterdam, UK: Elsevier.

[21] West RG 1963 Problems of the British Quaternary. Proc. Geol. Assoc. 74, 147–186. (doi:10.1016/S0016-7878(63)80031-0)

[22] West RG 2015 Evolution of a Breckland landscape: chalkland under a cold climate in the area of Beachamwell, Norfolk. Ipswich, UK: Suffolk Naturalists' Society.

[23] Rose J 1994 Major river systems of central and southern Britain during the Early and Middle Pleistocene. Terra Nova 6, 435–443. (doi:10.1111/j.1365-3121.1994.tb00887.x)

[24] Rose J 2009 Early and Middle Pleistocene landscapes of eastern England. Proc. Geol. Assoc. 120, 3–33. (doi:10.1016/j.pgeola.2009.05.003)

[25] Lewis SG 1993 The status of the Wolstonian glaciation in the English Midlands and East Anglia. PhD thesis, University of London.

[26] Lee JR, Rose J, Hamblin RJO, Moorlock BSP 2004 Dating the earliest lowland glaciation of eastern England: the pre-Anglian early Middle Pleistocene Happisburgh Glaciation. Quat. Sci. Rev. 23, 1551–1566. (doi:10.1016/j.quascirev.2004.02.002)

[27] Gibbard PL, Turner C, West RG 2013 The Bytham river reconsidered. Quat. Int. 292, 15–32. (doi:10.1016/j.quaint.2012.08.2053)

[28] Miall AD 1978 Lithofacies types and vertical profile models in braided rivers: a summary. In Fluvial sedimentology (ed. AD Miall), pp. 597–604. Memoir 5. Canadian Society for Petroleum Geologists.

[29] Eyle N, Eyles CH, Miall AD 1983 Lithofacies types and vertical profile models: an alternative approach to the description and environmental interpretation of glacial diamict sequences. Sedimentology 30, 393–410.

[30] Wymer JJ 1985 Palaeolithic sites of East Anglia. Norwich, UK: Geobooks.

[31] Solomon JD 1933 The implementiferous gravels of Warren Hill. J. R. Anthropol. Inst. 63, 101–110.

[32] Paterson TT 1942 *Lower Palaeolithic man in the Cambridge district* PhD thesis, University of Cambridge, Cambridge, UK.

[33] Wymer JJ, Lewis SG, Bridgland DR 1991 Warren Hill, Mildenhall, Suffolk. Tottenhill. In Central East Anglia & the Fen basin field guide (eds Lewis SG, Whiteman CA Bridgland DR), pp. 50–58. Cambridge, UK: Quaternary Research Association.

[34] Lewis SG 1992 High Lodge—stratigraphy and depositional environments. In High Lodge: excavations by G. de G. Sieveking 1962–8 and J. Cook 1988 (eds Ashton NM, Cook J, Lewis SG, Rose J), pp. 51–85. London, UK: British Museum Press.

[35] Francis EA 1992 Structural studies in and around High Lodge. In High Lodge: excavations by G. de G. Sieveking, 1962–8 and J. Cook. 1988 (eds Ashton NM, Cook J, Lewis SG, Rose J), pp. 86–93. London, UK: British Museum Press.

[36] Nicholls GD 1947 Introduction to the geology of Bedfordshire. J. Bedfordshire Nat. Hist. Soc. Field Club 2, 9–16.

[37] Wells AK, Gossling F, Kirkaldy J, Oakley KP 1947 Studies of pebbles from the lower Cretaceous rocks (Weald Committee, Report No. 37). Proc. Geol. Assoc. 58, 194–258. (doi:10.1016/S0016-7878(47)80009-4)

[38] Hawkes L 1951 The erratics of the English Chalk. Proc. Geol. Assoc. 62, 257–268. (doi:10.1016/S0016-7878(51)80012-9)

[39] Chatwin CP 1961 British regional geology: East Anglia and adjoining areas, 4th edn London, UK: Her Majesty's Stationery Office.

[40] Clarke MR, Auton CA 1984 Ingham sand and gravel. In Field guide to the Gipping and Waveney valleys, Suffolk (ed. Allen P), pp. 71–72. Cambridge, UK: Quaternary Research Association.

[41] Hey RW, Auton CA 1988 Compositions of pebble-beds in the Neogene and pre-Anglian Pleistocene of East Anglia. In Pliocene-Middle Pleistocene of East Anglia, field guide (eds Gibbard PL, Zalasiewicz JA), pp. 35–41. Cambridge, UK: Quaternary Research Association.

[42] Rose J 1987 Status of the Wolstonian glaciation in the British Quaternary. Quat. Newsl. 53, 1–9.

[43] Lewis SG, Bridgland DR 1991 Ingham and Timworth, Suffolk. In Central East Anglia & the Fen basin field guide (eds Lewis SG, Whiteman CA, Bridgland DR), pp. 71–83. Cambridge, UK: Quaternary Research Association.

[44] Lewis SG, Rose J, Davies H 1999 Pre-Anglian fluvial and Anglian glaciogenic sediments, Knettishall, Suffolk, England. Proc. Geol. Assoc. 110, 17–32. (doi:10.1016/S0016-7878(99)80003-0)

[45] Rose J, Wymer JJ 1994 Record of a struck flake and the lithological composition of ‘preglacial’ river deposits, Hengrove, Suffolk, England. Proc. Suffolk Inst. Archaeol. His. 28, 119–125. (doi:10.1007/BF03377150)

[46] Prestwich J 1861 Notes on some further discoveries of flint implements in beds of post-Pliocene gravel and clay; with a few suggestions for search elsewhere. Q. J. Geol.Soc. Lond. 17, 362–368. (doi:10.1144/GSL.JGS.1861.017.01-02.30)

[47] Evans J 1879 *The ancient stone implements, weapons and ornaments of Great Britain*. London, UK: Longmans Green.

[48] Lewis SG 2012 Pleistocene stratigraphy and sedimentology. In Excavations at Lynford Quarry, Norfolk (eds Boismier WA, Gamble C, Coward F), pp. 17–32. Swindon, UK: English Heritage.

[49] Schwenninger J-L, Rhodes E 2012 Optically stimulated luminescence. In Neanderthals among mammoths (eds Boimeier WA et al.), pp. 67–69. London, UK: English Heritage.

[50] West RG 2009 From Brandon to Bun *gay: an exploration of the landscape history and geology of the Little Ouse and Waveney Rivers*, 106 pp Ipswich, UK: Suffolk Naturalists' Society.

[51] Watt AS 1940 Studies in the ecology of Breckland. IV. The grass-heath. J. Ecol. 28, 42–70.

[52] Watt AS 1936 Studies in the ecology of Breckland: I. Climate, soil and vegetation. J. Ecol. 24, 117–138. (doi:10.2307/2256271)

[53] West RG 2007 The Little Ouse River, the Waveney River and the Breckland: a joint history. Transactions of the Suffolk Naturalists' Society 43, 35–39.

[54] Gallois RW 1994 Geology of the country around King's Lynn and The Wash. Memoir Sheet 129 London, UK: British Geological Survey, Her Majesty's Stationery Office.

[55] Rawson PF 2006 Cretaceous: sea levels peak as the North Atlantic opens. In The geology of England and Wales (eds Brenchley PJ, Rawson PJ), pp. 365–393. Geological Society.

[56] Perrin RMS 1961 Soils. In Norwich and its regions (ed. F Briers), pp. 44–50. Norwich, UK: Jarrold.

[57] Worssam BC, Taylor JH 1969 Geology of the country around Cambridge. Memoir of the Geological Survey of Great Britain. London, UK: Her Majesty's Stationery Office.

[58] West RG 2017 Patterned ground and superficial deposits at Hare Park, Swaffham Bulbeck, Cambridgeshire. Proc. Yorkshire Geol. Soc. 62.

[59] Hodge CAH, Seale RS 1966 *The soils of the district around Cambridge* Memoirs of the Soil Survey of Great Britain: England and Wales Harpenden, UK: Agricultural Research Council.

[60] McKenny Hughes T 1916 The gravels of East Anglia. Cambridge, UK: Cambridge University Press.

[61] West RG, Gibbard PL 2017 The Observatory Gravels and the Travellers' Rest pit, Cambridge, England. Proc. Yorkshire Geological Society 61, 313 (doi:10.1144/pygs2017-392)

[62] Gao C, Boreham S 2011 Ipswichian (Eemian) floodplain deposits and terrace stratigraphy in the lower Great Ouse and Cam valleys, southern England, UK. Boreas 40, 303–319. (doi:10.1111/j.1502-3885.2010.00191.x)

[63] Marr JE, King WBR 1932 Further notes on the Huntingdon Road gravels, Cambridge. Geol.Mag. 69, 175–178. (doi:10.1017/S0016756800097090)

[64] Boreham S 2002 The Pleistocene stratigraphy and palaeoenvironments of the Cambridge district. PhD thesis, The Open University.

[65] Sparks BW, West RG 1959 The palaeoecology of the interglacial deposits at Histon Road, Cambridge. Eiszeitalter und Gegenwart 10, 123–143.

[66] Lambert CA, Pearson RG, Sparks BW, Dickson JH 1963 A flora and fauna from Late Pleistocene deposits at Sidgwick Avenue, Cambridge. Proc. Linnean Soc. Lond. 174, 13–29. (doi:10.1111/j.1095-8312.1963.tb00891.x)

[67] Marr JE, Gardner EW 1916 II. On some deposits containing an arctic flora in the Pleistocene Beds of Barnwell, Cambridge. Geol. Mag. 3, 339–343. (doi:10.1017/S0016756800205931)

[68] Godwin H, Willis EH 1964 Cambridge University natural radiocarbon measurements VI. Radiocarbon 6, 116–137. (doi:10.1017/S0033822200010602)

[69] Maddy DM 1999 English Midlands. In A revised correlation of the Quaternary deposits in the British Isles (ed. Bowen DQ), pp. 28–44. Geological Society Special Report no. 23.

[70] Harding P, Keen DH, Bridgland DR, Rogerson RJ 1991 A palaeolithic site rediscovered at Biddenham, Bedfordshire. Bedfordshire Archaeol. 19, 87–90.

[71] Preece RC, Ventris PA 1983 An Interglacial site at Galley Hill, near St. Ives, Cambridgeshire. Geol. Soc. Norfolk Bull. 33, 63–72.

[72] Gao C, Keen DH, Boreham S, Coope GR, Pettit ME, Stuart AJ, Gibbard PL 2000 Last Interglacial and Devensian deposits of the River Great Ouse at Woolpack Farm, Fenstanton, Cambridgeshire, UK. Quat. Sci. Rev. 19, 787–810. (doi:10.1016/S0277-3791(99)00028-1)

[73] Fisher O 1879 On a mammaliferous deposit at Barrington near Cambridge. Quarterly Journal of the Geological Society of London 35, 670.

[74] Hughes MC 1888 On the Mollusca of the Pleistocene Gravels in the neighbourhood of Cambridge. Geol. Magazine 5, 193–207.

[75] Marr JE 1920 The Pleistocene deposits around Cambridge. Quarterly Journal of the Geological Society of London 75, 204–242.

[76] Hollingworth SE, Allison J, Godwin H 1950 Interglacial deposits from the Histon Road, Cambridge. Quarterly Journal of the Geological Society of London 105, 495–509.

[77] Walker D 1953 The interglacial deposits at Histon Road, Cambridge. Quarterly Journal of the Geological Society of London 108, 273–282.

[78] Sparks BW 1964 A note on the Pleistocene deposit at Grantchester, Cambridgeshire. Geol. Magazine 101, 334–339.

[79] Gibbard PL, Stuart AJ 1974 Trace fossils from proglacial lake sediments. Boreas 3, 69–74.

[80] Gao C 1997 Sedimentology and stratigraphy of the River Great Ouse, southern England. PhD thesis, University of Cambridge, Cambridge, UK.

[81] Bridgland DR, Schreve DC 2001 River terrace formation in synchrony with long-term climatic fluctuation. In River basin sediment systems: archives of environmental change. London, UK: Taylor & Francis.

[82] West RG, Andrew R, Knudsen KL, Peglar SM, Pettit ME 1995 Late Pleistocene deposits at Chatteris, March and Wimblington, Cambridgeshire, UK. Proc. Geol. Assoc. 106, 195–210. (doi:10.1016/S0016-7878(08)80023-5)

[83] West RG, Burton RGO, Andrew R, Pettit ME 2002 Evolution of a periglacial landscape in the Late Devensian: environments and palaeobotany of the Mepal area, Cambridgeshire, England. J. Quat. Sci. 17, 31–50.

[84] Booth SJ 1982 The sand and gravel resources of the country around Whittlesey, Cambridgeshire. In Mineral Assessment Report No.93, Institute of Geological Sciences London, UK: Her Majesty's Stationery Office.

[85] Horton A 1989 Geology of the Peterborough district. British Geological Survey Memoir. Sheet 158 London, UK: Her Majesty's Stationery Office.

[86] Davey NDW 1991 A review of the Pleistocene geology of the Peterborough district. In Central East Anglia and the Fen basin, field guide, pp. 150–162. London, UK: Quaternary Research Association.

[87] Langford HE 1999 Sedimentological, palaeogeographical and stratigraphical aspects of the Middle Pleistocene geology of the Peterborough area, eastern England. PhD thesis, Anglia Polytechnic University.

[88] Langford HE 2012 Reply to Westaway *et al*. discussion of ‘A comment on the MIS 8 glaciation of the Peterborough area, eastern England’. Quat. Newsl. 127.

[89] Langford HE, Briant RM 2004 Nene Valley field guide. London, UK: Quaternary Research Association.

[90] Briant R 2002 Fluvial responses to rapid climate change in eastern England during the last glacial period. PhD thesis, University of Cambridge, Cambridge, UK.

[91] Horton A, Keen DH, Field MH, Robinson JE, Coope GR, Currant AP, Graham DK, Green CP, Phillips LM 1992 The Hoxnian interglacial deposits at Woodston, Peterborough. Phil. Trans. R. Soc. Lond. B 338, 131–164. (doi:10.1098/rstb.1992.0136)

[92] Keen DH, Robinson JE, West RG, Lowry F, Bridgland DR, Davey NDW 1990 The fauna and flora of the March Gravels at Northam Pit, Eye, Cambridgeshire, England. Geol, Mag. 127, 453–465. (doi:10.1017/S001675680001520X)

[93] Langford HE, Boreham S, Briant RM, Coope GR, Horne DJ, Schreve DC, Whittaker JE, Whitehouse NJ 2014 Middle to Late Pleistocene palaeoecological reconstructions and palaeotemperature estimates for cold/cool stage deposits at Whittlesey, eastern England. Quat. Int. 341, 6–26. (doi:10.1016/j.quaint.2014.01.037)

[94] Langford HE et al. 2014b Palaeoecology of a late MIS 7 interglacial deposit from eastern England. Quat. Int. 341, 27–45. (doi:10.1016/j.quaint.2013.05.046)

[95] Castleden R 1980 Fluvioglacial pedimentation. Catena 7, 135–152.

[96] Bridgland DR, Keen DH, Davey NDW 1991 The Pleistocene sequence in the Peterborough district: possible correlation with the deep sea oxygen isotope record. In Central East Anglia and the Fen Basin: field guide (eds SG Lewis, CA Whiteman, DR Bridgland), pp. 209–212. London, UK: Quaternary Research Association.

[97] Langford HE 2004 Middle Pleistocene deposits at Stanground (Peterborough) and March, eastern England: a discussion based on new evidence. Proc. Geol. Assoc. 115, 91–95. (doi:10.1016/S0016-7878(76)80051-X)

[98] West RG, Knudsen KL, Penney DN, Preece RC, Robinson JE 1994 Palaeontology and taphonomy of Late Quaternary fossil assemblages at Somersham, Cambridgeshire, England and the problem of reworking. J. Quater. Sci. 9, 357–366.

[99] West RG, Peglar SM, Pettit ME, Preece RC 1995 Late Pleistocene deposits at Block Fen, Cambridgeshire, England. J. Quat. Sci. 10, 285–310. (doi:10.1002/jqs.3390100307)

[100] Briant RM, Coope GR, Preece RC, Keen DH, Boreham S, Griffiths HI, Gibbard PL 2004 Fluvial system response to Late Devensian aridity, Baston, Lincolnshire, England. J. Quat. Sci. 19, 479–495. (doi:10.1002/jqs.851)

[101] Briant RM, Coope GR, Preece RC, Gibbard PL 2004 The Upper Pleistocene deposits at Deeping St James, Lincolnshire: evidence for Early Devensian fluvial sedimentation. Quaternaire 15, 5–15. (doi:10.3406/quate.2004.1750)

[102] Boreham S, White TS, Bridgland DR, Howard AJ, White MJ 2010 The Quaternary history of the Wash fluvial network UK. Proc. Geol. Assoc. 121, 393–409. (doi:10.1016/j.pgeola.2010.02.003)

[103] Langford HE et al. 2004 Funtham's Lane East. In Nene Valley, field guide (eds Langford HE, Briant RM), pp. 69–106. Cambridge, UK: Quaternary Research Association.

[104] Whitaker W (ed.), Skertchly SBJ, Jukes-Browne AJ 1893 The geology of south-western Norfolk and of northern Cambridgeshire. Memoirs of the Geological Survey, London, UK: England and Wales.

[105] Gallois RW 1978 Geological investigations for the Wash water storage scheme. Reports of the Institute of Geological Sciences, No. 78/19.

[106] Gallois RW 1978 The Pleistocene history of west Norfolk. Bull. Geol. Soc. Norfolk 30, 3–38.

[107] Ventris PA 1985 Pleistocene environmental history of the Nar Valley, Norfolk. PhD dissertation, University of Cambridge, Cambridge, UK.

[108] Ventris PA 1986 The Nar Valley. In The Nar Valley and North Norfolk, field guide (eds West RG, Whiteman CA), pp. 7–55. Coventry, UK: Quaternary Research Association.

[109] West RG, Dickson CA, Catt JA, Weir AH, Sparks BW 1974 Late Pleistocene deposits at Wretton, Norfolk. II. Devensian deposits. Phil. Trans. R. Soc. Lond. B 267, 337–420. (doi:10.1098/rstb.1974.0004) 10.1098/rstb.1970.0030 22408823

[110] Corser CE 1982 The sand and gravel resources of the country north of Newmarket. Description of 1:25 000 Sheet TL 67 and part of TL66. In Mineral Assessment Report No. 110, Institute of Geological Sciences.

[111] Clayton AR 1983 The sand and gravel resources between Mildenhall and Barrow, Suffolk. Mineral Assessment Report No. 123, Institute of Geological Sciences, London, UK: Her Majesty's Stationery Office.

[112] West RG et al. 1999 Late and Middle Pleistocene deposits at Somersham, Cambridgeshire, UK: a model for reconstructing fluvial/estuarine depositional environments. Quat. Sci. Rev. 18, 1247–1314. (doi:10.1016/S0277-3791(98)00067-5)

[113] Baden-Powell DFW 1934 On the marine gravels at March, Cambridgeshire. Geol. Mag. 71, 193–219. (doi:10.1017/S0016756800093146)

[114] West RG 1993 On the history of the Late Devensian Lake Sparks in southern Fenland, Cambridgeshire, England. J. Quat. Sci. 8, 217–234. (doi:10.1002/jqs.3390080304)

[115] Sedgwick A 1846 On the Geology of the neighbourhood of Cambridge, including the formations between the Chalk escarpment and the Great Bedford Level. Report of the British Association for 1845, Transactions of Sections, 40.

[116] Fisher O 1868 The Boulder-Clay at Witham and the Thames Valley. Geol. Mag. 5, 98–100. (doi:10.1017/S0016756800207413)

[117] Bonney TG 1875 Cambridgeshire geology: A sketch for the use of students. Deighton, Bell.

[118] Reed FRC 1897 Handbook to the geology of Cambridgeshire. Cambridge, UK: Cambridge University Press.

[119] Evans DJA, Rea BR 1999 Geomorphology and sedimentology of surging glaciers: a land-systems approach. Ann. Glaciol. 28, 75–82. (doi:10.3189/172756499781821823)

[120] Aber JS, Ber A. 2007 Glaciotectonism: developments in Quaternary Science, vol. 6, p. 246 Amsterdam, The Netherlands: Elsevier.

[121] Bluemle JP, Clayton LEE 1984 Large-scale glacial thrusting and related processes in North Dakota. Boreas 13, 279–299. (doi:10.1111/j.1502-3885.1984.tb01124.x)

[122] Gry H 1940 De istektoniske forhold i moleromraadet. Copenhagen, Denmark: F. Bagges kgl. hofbogtrykkeri.

[123] Maarleveld GC 1953 Standen van het landijs in Nederland. Boor en Spade 6, 95–105.

[124] van den Berg MW, Beets DJ 1987 Saalian glacial deposits and morphology in The Netherlands. In Tills and Glaciotectonics: Proc. INQUA Symp. on the Genesis and Lithology of Glacial Deposits (1986) (ed. Van der Meer JJM), pp. 235–251. Rotterdam, The Netherlands: Balkema.

[125] Benn DI, Evans DJA 2010 Glaciers and glaciation. London, UK: Hodder Education.

[126] Clark PU 1992 Surface form of the southern Laurentide ice sheet and its implications to ice-sheet dynamics. Geol. Soc. Am. Bull. 104, 595–605. (doi:10.1130/0016-7606(1992)104<0595:SFOTSL>2.3.CO;2)

[127] Benn DI, Hulton NRJ 2010 An Excel™ spreadsheet program for reconstructing the surface profile of former mountain glaciers and ice caps. Comput. Geosci. 36, 605–610. (doi:10.1016/j.cageo.2009.09.016)

[128] Shoemaker EM 1992 Subglacial floods and the origin of low-relief ice-sheet lobes. J. Glaciol. 38, 105–112. (doi:10.1017/S0022143000009643)

[129] Bell AE 2008 The role of subglacial water in ice-sheet mass balance. Nat. Geosci. 1, 297–304. (doi:10.1038/ngeo186)

[130] Copeland L, Sharp MJ, Dowdeswell JA 2003 The distribution and flow characteristics of surge-type glaciers in the Canadian High Arctic. Ann. Glaciol. 36, 73–81. (doi:10.3189/172756403781816301)

[131] Langford HE 2012 A comment on the MIS 8 glaciation of the Peterborough area, eastern England. Quat. Newsl. 127, 6–20.

[132] Horton A 1974 The Geology of Peterborough. British Geological Survey Memoir. Sheet 158 London, UK: Her Majesty's Stationery Office.

[133] Clayton L, Teller JT, Attig JW 1985 Surging of the southwestern part of the Laurentide Ice Sheet. Boreas 14, 235–241. (doi:10.1111/j.1502-3885.1985.tb00726.x)

[134] Tveranger J, Astakhov V, Mangerud J, Svendsen JI 1999 Surface form of the last Kara Ice Sheets as inferred from its southwestern marginal features. Boreas 28, 81–91. (doi:10.1111/j.1502-3885.1999.tb00207.x)

[135] Sharp MJ 1988 Surging glaciers: geomorphic effects. Prog. Phys. Geogr. 12, 533–559. (doi:10.1177/030913338801200403)

[136] Wingfield RTR, Evans CDR, Deegan SE, Floyd R 1978 Geological and geophysical survey of The Wash. Institute of Geological Sciences Report 78.18, 32 pp.

[137] Gibbard PL, West RG, Turner C 2013 The Bytham river reconsidered. Quat. Internat. 292, 15–32.

[138] Cameron TDJ, Crosby A., Balson PS, Jeffery DH, Lott GK, Bulat J, Harrison DJ 1992 United Kingdom offshore regional report: the geology of the southern North Sea. London, UK: Her Majesty's Stationery Office.

[139] Ansari MH 1992 *Stratigraphy and palaeobotany of Middle Pleistocene interglacial deposits in the North Sea*, pp. 303 PhD thesis, University of Wales.

[140] Ventris PA 1996 Hoxnian Interglacial freshwater and marine deposits in northwest Norfolk, England and their implications for sea-level reconstruction. Quat. Sci. Rev. 15, 437–450. (doi:10.1016/0277-3791(96)00020-0)

[141] Litt T, Turner C 1993 Arbeitsergebnisse der Subkommission für Europäische Quartärstratigraphie: Die Saalesequenz in der Typusregion (Berichte der SEQS 10). Eiszeitalter und Gegenwart 43, 125–128.

[142] Pawley SM, Bailey RM, Rose J, Moorlock BSP, Hamblin RJO, Booth SJ, Lee JR 2008 Age limits on Middle Pleistocene glacial sediments from OSL dating, north Norfolk, UK. Quat. Sci. Rev. 27, 1363–1377. (doi:10.1016/j.quascirev.2008.02.013)

[143] Pawley S, Busschers FS In press.

[144] Straw A 2000 Some observations on ‘Eastern England’ in ‘A Revised Correlation of Quaternary deposits in the British Isles’, (ed. DQ Bowen, 1999). Quat. Newsl. 91, 2–6.

[145] Straw A 2005 Glacial and pre-glacial deposits at Welton-le-Wold, Lincolnshire, 39 pp Exeter, UK: Studio Publishing.

[146] Straw A 2011 The Saale glaciation of eastern England. Quat. Newsl. 123, 28–35.

[147] Straw A 2015 The Quaternary sediments at Welton-le-Wold, Lincolnshire. Mercian Geol. 18, 228–233.

[148] White TS, Bridgland DR, Westaway R, Howard AJ, White MJ 2010 Evidence from the Trent terrace archive, Lincolnshire, UK, for lowland glaciation of Britain during the Middle and Late Pleistocene. Proc. Geol. Assoc. 121, 141–153. (doi:10.1016/j.pgeola.2010.05.001)

[149] White TS, Bridgland DR, Westaway R, Straw A 2016 Evidence for late Middle Pleistocene glaciation of the British margin of the southern North Sea. J. Quat. Sci. 32, 261–271. (doi:10.1002/jqs.2826)

[150] Schenninger J-L, Bridgland DR, Howard AJ, White TS 2007 Optically stimulated luminescence dating of the Trent Valley sediments: problems and preliminary results. In The Quaternary of the Trent Valley and adjoining regions field guide (eds White TS et al.), pp. 62–65. London, UK: Quaternary Research Association.

[151] Green C 2011 The origins of Louth, 177pp Louth, UK: Lindes.

[152] Busschers FS, van Balen RT, Cohen KM, Kasse C, Weerts HJT, Wallinga J, Bunnik FPM 2008 Response of the Rhine–Meuse fluvial system to Saalian ice-sheet dynamics. Boreas 37, 377–398. (doi:10.1111/j.1502-3885.2008.00025.x)

[153] Toucanne S et al. 2009 Timing of massive ‘Fleuve Manche’ discharges over the last 350 kyr: insights into the European ice-sheet oscillations and the European drainage network from MIS 10 to 2. Quat. Sci. Rev. 28, 1238–1256. (doi:10.1016/j.quascirev.2009.01.006)

[154] Ehlers J 2011 Das Eiszeitalter. Heidelberg, Germany: Spektrum Akademischer Verlag.

[155] Ehlers J, Grube A, Stephan H-J, Wansa S 2011 Pleistocene glaciations of North Germany—new results. In Quaternary glaciation extent and chronology: a closer look, Ch. 13 (eds Ehlers J, Gibbard PL, Hughes PD), pp. 149–162. Amsterdam, The Netherlands: Elsevier.

[156] Moreau J, Huuse M, Gibbard PL, Moscariello A 2009 3D seismic megasurvey geomorphology of the southern North Sea, tunnel valley record and associated ice-sheet dynamic In *71st EAGE Conference & Exhibition, Amsterdam, The Netherlands, 8–11 June 2009*.

[157] Moreau J 2010 The seismic analysis of the southern North Sea glaciogenic record. GRASP Report No. 1 Delft, The Netherlands.

[158] Rappol M 1987: Saalian till in The Netherlands: a review. In *Tills and glaciotectonics* (ed. JJM van der Meer), pp. 3–21. Rotterdam, The Netherlands: A. A. Balkema.

[159] Busschers FS et al. 2007 Late Pleistocene evolution of the Rhine in the southern North-Sea Basin: imprints of climate change, sea-level oscillations and glacio-isostasy. Quat. Sci. Rev. 26, 3216–3248. (doi:10.1016/j.quascirev.2007.07.013)

[160] Gibbard P 2007 Europe cut adrift. Nature 448, 259–260. (doi:10.1038/448259a) 17637645 10.1038/448259a

[161] Mathys M 2009 *The Quaternary geological evolution of the Belgian Continental Shelf, southern North Sea*, p. 454 PhD thesis, Ghent University, Ghent.

[162] Hijma MP, Cohen KM, Roebroeks W, Westerhoff WE, Busschers FS 2012 Pleistocene Rhine–Thames landscapes: geological background for hominin occupation of the southern North Sea region. J. Quat. Sci. 27, 17–39. (doi:10.1002/jqs.1549)

[163] Cohen KM, Gibbard PL, Weerts HJT 2014 North Sea palaeogeographical reconstructions for the last 1 Ma. Neth. J. Geosci. Geol. Mijnbouw 93, 7–29. (doi:10.1017/njg.2014.12)

[164] Head MJ, Gibbard PL 2015 Early–Middle Pleistocene transitions: linking terrestrial and marine realms. Quat. Int. 389, 7–46. (doi:10.1016/j.quaint.2015.09.042)

[165] Railsback LB et al. 2014 A stalagmite record of abrupt climate change and possible westerlies-derived atmospheric precipitation during the Penultimate Glacial Maximum in northern China. Palaeogeogr. Palaeoclimatol. Palaeoecol. 393, 30–44. (doi:10.1016/j.palaeo.2013.10.013)

[166] Railsback LB, Gibbard PL, Head MJ, Voarintsoa NRG, Toucanne S 2015 An optimized scheme of lettered marine isotope substages for the last 1.0 million years, and the climatostratigraphic nature of isotope stages and substages. Quat. Sci. Rev. 111, 94–106. (doi:10.1016/j.quascirev.2015.01.012)

[167] Turner C 2002 Formal status and vegetational development of the Eemian interglacial in northwestern and southern Europe. Quat. Res. 58, 41–44. (doi:10.1006/qres.2002.2365)

[168] West RG 1972 Relative land-sea level changes in south-eastern England during the Pleistocene. Phil. Trans. R. Soc. Lond. A 272, 87–98. (doi:10.1098/rsta.1972.0034)

[169] Rose J, Candy I, Lee JR 2000 Leet Hill (TM384926): pre-glacial and glaciofluvial river deposits – with possible evidence for a major glaciation prior to the deposition of the Lowestoft Till. In The Quaternary of Norfolk and Suffolk, field guide (eds Lewis SG, Whiteman CA, Preece RC), pp. 207–218. London, UK: Quaternary Research Association.

[170] Lewis SG 1999 Ch.2—Eastern England. In Bowen DQ. A revised correlation of the Quaternary deposits in the British Isles, Geological Society Special Report, 23, 10–27.

[171] Clayton KM 2000 The landform changes brought about by the Anglian glaciation. In The Quaternary of Norfolk and Suffolk: field guide (eds Lewis SG, Whiteman CA, Preece RC), pp. 55–60. London, UK: Quaternary Research Association.

[172] Belshaw RK, Gibbard PL, Murton JB, Murton DK 2014 Early Middle Pleistocene drainage in southern central England. Neth. J. Geosci. Geol. en Mijnbouw 93, 135–145. (doi:10.1017/njg.2014.25)

[173] Woodland AW 1970 The buried tunnel-valleys of East Anglia. Proc. Yorkshire Geol. Soc. 37, 521–578. (doi:10.1144/pygs.37.4.521)

[174] Cox FC 1985 The tunnel-valleys of Norfolk, East Anglia. Proc. Geol. Assoc. 96, 357–369. (doi:10.1016/S0016-7878(85)80024-9)

[175] Cox FC, Nickless EFP 1972 Some aspects of the glacial history of central Norfolk. London, UK: Her Majesty's Stationery Office.

[176] van der Vegt P, Janszen A, Moreau J, Gibbard P, Huuse M, Moscariello A 2009 Glacial sedimentary systems and tunnel valleys of East Anglia, England. GRASP Abstract. In *Glaciogenic Reservoirs and Hydrocarbons, Geological Society London, 1–2 December*.

[177] van der Vegt P, Janszen A, Moscariello A 2012 Tunnel valleys: current knowledge and future perspectives. Geol. Soc. Lond. Spec. Publ. 368, 75–97. (doi:10.1144/SP368.13)

[178] Ehlers J, Meyer KD, Stephan HJ 1984 The pre-Weichselian glaciations of North-West Europe. Quat. Sci. Rev. 3, 111–940. (doi:10.1016/0277-3791(84)90003-9)

[179] Horton A 1970 The drift sequence and subglacial topography in parts of the Ouse and Nene basin. Institute of Geological Sciences Report 70/9. London, UK: Her Majesty's Stationery Office.

[180] Stevens LA 1960 The interglacial of the Nar Valley, Norfolk. Quart. J. Geol. Soc. Lond. 115, 291–315. (doi:10.1144/GSL.JGS.1959.115.01.14)

[181] Horton A 1981 The Peterborough area—the glacial deposits. In Field guide to the East Midlands region (ed. Douglas TD), pp. 27–35. Leicester, UK: Quaternary Research Association.

[182] Lewis SG, Whiteman CA, Bridgland DR (eds). 1991 Central East Anglia and the Fen basin, field guide. London, UK: Quaternary Research Association.

[183] Bristow CR 1990 Geology of the country around Bury St Edmunds: memoir for 1: 50 000 geological sheet 189 (England and Wales). London, UK: Her Majesty's Stationery Office.

[184] Green CP et al. 1996 Pleistocene deposits at Stoke Goldington, in the valley of the Great Ouse, UK. J. Quat. Sci. 11, 59–87. (doi:10.1002/(SICI)1099-1417(199601/02)11:1<59::AID-JQS218>3.0.CO;2-7)

[185] Gibbard PL, Lewin J 2002 Climate and related controls on interglacial fluvial sedimentation in lowland Britain. Sediment. Geol. 151, 187–210. (doi:10.1016/S0037-0738(01)00253-6)

[186] Peeters J, Busschers FS, Stouthamer E, Bosch JHA, Van den Berg MW, Wallinga J, Versendaal AJ, Bunnik FPM, Middelkoop H 2016 Sedimentary architecture and chronostratigraphy of a late Quaternary incised-valley fill: a case study of the late Middle and Late Pleistocene Rhine system in The Netherlands. Quat. Sci. Rev. 131, 211–236. (doi:10.1016/j.quascirev.2015.10.015)

[187] Sparks BW, West RG 1970 Late Pleistocene deposits at Wretton, Norfolk. I. Ipswichian interglacial deposits. Phil. Trans. R. Soc. Lond. B 258, 1–30. (doi:10.1098/rstb.1970.0030) 22408823 10.1098/rstb.1970.0030

[188] Burton RGO, Hodgson JM (eds) 1987 Lowland peat in England and Wales. Harpenden, UK: Soil Survey of England and Wales.

[189] Flower JW 1869 On some recent discoveries of flint implements of the Drift in Norfolk and Suffolk, with observations on the theories accounting for their distribution. Quart. J.Geol. Soc. 25, 449–460. (doi:10.1144/GSL.JGS.1869.025.01-02.79)

[190] West RG 1987 A note on the March Gravels and Fenland sea levels. Geol. Soc. Norfolk, Bull. 37, 27–34.

[191] West RG 1999 A possible present day analogue for the origin of the marine fauna of the Late Pleistocene March Gravels of the Fenland. *Bulletin of the Geological Society of Norfolk* 46, 45–52.

[192] Shotton FW, Blundell DJ, Williams R 1969 Birmingham University radiocarbon dates III. Radiocarbon 11, 263–270. (doi:10.1017/S0033822200011218)

[193] Salvador A 1994 International stratigraphic guide: a guide to stratigraphic classification, terminology and procedures, 220 pp, 2nd edn Boulder, CO: Geological Society of America.

[194] Räsänen ME, Auri JM, Huitti JV, Klap AK, Virtasalo JJ, 2009 A shift from lithostratigraphic to allostratigraphic classification of Quaternary glacial deposits. Geol. Soc. Am. Today 19, 4–11.

[195] Hughes PD 2010 Geomorphology and Quaternary stratigraphy: the roles of morpho-, litho-, and allostratigraphy. Geomorphology 123, 189–199. (doi:10.1016/j.geomorph.2010.07.025)

[196] Baden-Powell DFW 1950 Palaeoliths from the Fen District. Proc. Prehistoric Soc. (New Series) 16, 29–41. (doi:10.1017/S0079497X00018892)

[197] West RG, Gibbard PL, Boreham S, Rolfe C 2014 Geology and geomorphology of the Palaeolithic site at High Lodge, Mildenhall, Suffolk, England. Proc. Yorkshire Geological Society. (doi:10.1144/pygs2014-347)

